# Characterization and comparative analysis of the complete plastid genomes of four *Astragalus* species

**DOI:** 10.1371/journal.pone.0286083

**Published:** 2023-05-23

**Authors:** Mahtab Moghaddam, Martin F. Wojciechowski, Shahrokh Kazempour-Osaloo

**Affiliations:** 1 Department of Plant Biology, Faculty of Biological Sciences, Tarbiat Modares University, Tehran, Iran; 2 School of Life Science, Arizona State University, Tempe, Arizona, United States of America; University of Naples Federico II, ITALY

## Abstract

*Astragalus* is the largest flowering plant genus. We assembled the plastid genomes of four *Astragalus* species (*Astragalus iranicus*, *A*. *macropelmatus*, *A*. *mesoleios*, *A*. *odoratus*) using next-generation sequencing and analyzed their plastomes including genome organization, codon usage, nucleotide diversity, prediction of RNA editing and etc. The total length of the newly sequenced *Astragalus* plastomes ranged from 121,050 bp to 123,622 bp, with 110 genes comprising 76 protein-coding genes, 30 transfer RNA (tRNA) genes and four ribosome RNA (rRNA) genes. Comparative analysis of the chloroplast genomes of *Astragalus* revealed several hypervariable regions comprising three non-coding sites (*trn*Q(UUG)–*acc*D, *rps*7 –*trn*V(GAC) and *trn*R(ACG)–*trn*N(GUU)) and four protein-coding genes (*ycf*1, *ycf*2, *acc*D and *clp*P), which have potential as molecular markers. Positive selection signatures were found in five genes in *Astragalus* species including *rps*11, *rps*15, *acc*D, *clp*P and *ycf*1. The newly sequenced species, *A*. *macropelmatus*, has an approximately 13-kb inversion in IR region. Phylogenetic analysis based on 75 protein-coding gene sequences confirmed that *Astragalus* form a monophyletic clade within the tribe Galegeae and *Oxytropis* is sister group to the Coluteoid clade. The results of this study may helpful in elucidating the chloroplast genome structure, understanding the evolutionary dynamics at genus *Astragalus* and IRLC levels and investigating the phylogenetic relationships. Moreover, the newly plastid genomes sequenced have been increased the plastome data resources on *Astragalus* that can be useful in further phylogenomic studies.

## Introduction

The chloroplast is a semi-autonomous organelle that plays important roles in photosynthesis, carbon fixation, and fatty acids, starch, and amino acids synthesis [1]. The chloroplast genomes (plastomes) of most angiosperms have a circular quadripartite structure composed of two identical copies of inverted repeat regions (IRa and IRb) that divide the rest of the plastome into LSC (large single-copy) and SSC (small single-copy) region [2]. There are about 80 protein-coding regions, four rRNA genes, and 30 tRNA genes in the plastome of land plants with the average size of 151 kb. Due to their slower evolution than nuclear genomes, absence of recombination, and prevalence of uniparental inheritance, plastid genome sequences have been shown to be useful molecular resources for phylogenetic analyses and elucidating the genetic relationships among taxa [3]. In terms of structural organization, gene/intron content, and gene order, the plastid genomes of photosynthetic green plants are generally highly conserved [2, 4, 5]. However, extensive rearrangements of plastomes such as IR lost have been reported in some lineages including Geraniaceae [6], Orobanchaceae [7–9] and Fabaceae [10, 11]. Fabaceae, the third-largest angiosperm family, includes a large clade of over 4000 species, 52 genera and nine tribes known as IRLC (Inverted Repeat Lacking Clade), which are distinguished by the absence of a single copy of the IR region [10, 12–14]. The plastomes of the IRLC have undergone many rearrangements, including numerous gene/intron losses [15–17], sequence inversions [18, 19], gene transfers to the nucleus [16, 20] and the second independent IR gain in some lineages [19, 21]. The two main explanations presented for genomic instability in the IRLC are the absence of the IR and repeat-mediated recombination [18, 21–23]. *Astragalus*, the largest genus in flowering plants and legumes, belongs to the tribe Galegeae in the IRLC. This genus contains approximately 2500–3000 species distributed on all continents except Australia, primarily in the northern hemisphere’s and South America’s cool and arid continental regions [24, 25]. In recent years, due to the rapid advancement of next-generation sequencing (NGS) technology, plastid genomes of about 38 *Astragalus* species (including 25 species of Neo-Astragalus (the New World aneuploid species) [24, 26] and the rest of species belong to other clades) have been deposited in NCBI (the National Center for Biotechnology Information). In the previous study, the connection between repeat structure and plastome variation among the New World *Astragalus* species was investigated [27]. Accordingly, *Astragalus* may be an appropriate group to investigate how the plastid genome structure and content have changed on a fine scale throughout evolution. On the other hand, other previous studies [28, 29] only sampled a small number of *Astragalus* species in comparative analyses.

In this study, we sequenced the chloroplast genomes of four *Astragalus* species (*Astragalus iranicus* Bunge, *A*. *macropelmatus* Bunge, *A*. *mesoleios* Boiss. et Hohen. and *A*. *odoratus* Lam.) and performed detailed comparative genomic analyses with previously reported plastid genomes of *Astragalus* as well as the rest of the IRLC plastomes. We aimed to 1) recognize the plastid genome structure, gene order and gene content of *Astragalus* species 2) investigate the origin, pattern, evolution and phylogenetic utility of plastid genome rearrangements in *Astragalus* 3) assess the effectiveness of complete chloroplast genome sequences in phylogenetic studies and 4) screen the highly informative regions of *Astragalus* plastomes for future Sanger based studies.

## Materials and methods

### Extraction and sequencing of plastid DNA

The young leaves of three *Astragalus* species (*A*. *iranicus*, *A*. *macropelmatus*, *A*. *mesoleios*) were collected from the southern and northern slopes of Alborz mountain chain in Tehran, Iran. *A*. *odoratus* was collected from an orchard, in Urmia, W. Azerbaijan, Iran. They were identified by Prof. S. Kazempour-Osaloo. These species were kept in the Tarbiat Modares University Herbarium (TMUH) (voucher code: 2016–2, 2016–3, 2016–4 and 2016–5, respectively). No permit was required to take the samples that were not on the list of national key protected plants. The fresh leaves were quickly dried with silica gel before DNA extraction. Our experimental research, composing the plant materials collection, follows international, national and institutional guidelines. Genomic DNA was extracted from dried leaves using a DNeasy Plant Kit (Qiagen) based on the manufacturer’s instructions. Illumina miSeq-550 platform was used for DNA sequencing at Arizona State University after its quality and quantity were assessed using 1% agarose gel electrophoresis. The paired-end libraries were constructed in accordance with the manufacturer’s instructions (Illumina Inc., San Diego, CA).

### Genome assembly and annotation

FastQC [30] was used to compare the quality of the generated short read data across species. The generated sequencing data was used to de novo assemble plastid genomes with Velvet v.1.2.10 [31] by generating contigs with different kmer values. To confirm the Velvet assemblies, NOVOPlasty [32], as another assembly method, was performed for each *Astragalus* species and *mat*K sequence of *Astragalus nakaianus* (KR296789) was used as seed. The newly assembled plastomes were annotated using GeSeq [33]. We improved the identification of tRNAs by using the on-line tRNAscan-SE service [34]. Raw read data were remapped to the assembled plastid genomes with Bowtie2 [35] (implemented in Geneious v.9.0.2 (https://www.geneious.com)) to specify the number of matched reads and to assess the depth of coverage. The entire plastome sequences of *A*. *iranicus*, *A*. *macropelmatus*, *A*. *mesoleios* and *A*. *odoratus* were deposited in GenBank.

The absence of IRa in the *Astragalus* species was confirmed using PCR and Sanger sequencing. A PCR method was used to determine whether or not the IRa region was present using diagnostic primer pairs. The primer pairs were designed to detect the presence or absence of the IRa region in either the conserved protein coding sequences *ndh*F and *psb*A or the *rps*19 and *rpl*2 protein coding regions that surround the IR region borders. In the present study, the following primer pairs were used: ndhF-F (5′-TATATGATTGGTCATATAATCG-3′) [36] and psbA-R (5′-GTTATGCATGAACGTAATGCTC-3′) [37]; rps19-F (5′-GTTCTGGACCAAGTTATT-3′) [36] and rpl2-R (5′-ATTTGATTCTTCGTCGAC-3′) [38]. The PCR amplification program implemented in this study was completely similar to the program used in the article by Moghaddam et al. (2022) [38].

In addition, the presence/absence of the inversion observed in *A*. *macropelmatus* was surveyed using PCR and Sanger sequencing in this species. In this regard, pairs of primers flanking the endpoints of inversion were designed in either *ycf*1 and *ndh*B or *trn*L(CAA) and *ndh*B conserved protein-coding sequences. The following primer pairs were used to assess the presence/absence of the inversion: ycf1-F (5’-CAATAGATAATGTGGTCAGA-3’) and ndhB-R (5’-ACCCAAACAAGTATGAAACG-3’); and trnL(CAA)-R (5’-ACCATTTCACCACCAAGGC-3’) and ndhB-F (5’-ACCCAAACAAGTATGAAACG-3’) (designed in this study). Amplification condition was performed in a 20 μl reaction volume and consisting of 8.5 μl deionized water, 10 μl Tag red master mix (Amplicon), 0,5 μl forward primer, 0,5 μl reverse primer and 1 μL template. Mixture solution was amplified by PCR machine (Biorad). Thermal cycle programmed for 3 min at 95°C as initial denaturation, followed by 37 cycles of 60 sec at 95°C for denaturation, 80 sec at 53°C (when using ycf1-F and ndhB-R primers) and 45 sec at 56°C (when using ndhB-F and trnL(CAA)-R primers) as annealing, 70 sec at 72°C for extension, and final extension at 72°C for 7 min. PCR products were examined by electrophoresis at 100 V for 30 minutes in a 1% (w/v) agarose gel in 1 x TAE buffer. Electrophoresis gel was soaked in ethidium bromide for 30 minutes then visualized in UV light.

### Codon usage analysis

Codon usage analysis was conducted using the Bioinformatics web server (https://www.bioinformatics.org/sms2/codon_usage.html). MEGA11 [39] was also used to determine the relative synonymous codon usage (RSCU) values, which were used to show the characteristics of the variation in synonymous codon usage.

### Determination of repeat sequences

REPuter [40] was used to recognize different types of repeat sequences (forward, reverse, complementary and palindromic sequences), (with a minimal size = 30 bp, hamming distance = 3 and greater than 90% identity). MISA, a microsatellite identification tool (available online: http://pgrc.ipk-gatersleben.de/misa/misa.html), was used to identify simple sequence repeats (SSRs). The minimum numbers of the SSR motifs were 10, 5, 4, 3, 3 and 3 for mono-, di-, tri-, tetra-, penta-, and hexanucleotide repeats, respectively.

### Identification of divergent hotspots and analysis of synonymous (Ks) and non-synonymous (Ka) substitution rates

The whole chloroplast genome sequences were aligned using MAFFT [41] on XSEDE v.7.402 in CIPRES Science Gateway [42] to determine nucleotide diversity (Pi) among the plastomes of the four newly sequenced *Astragalus* species as well as some *Astragalus* representative species. Using the DnaSP v.6.12 software [43], a sliding window analysis was performed to show the nucleotide diversity of the plastid genome. The window length was set to 800 bp and the step size was 200 bp. Moreover, the protein coding regions of the 20 plastomes were used to assess evolutionary rate differences within the *Astragalus*. Thus, we used MAFFT to align the 76 protein-coding regions separately, and then implemented DnaSP v.6.12 software to estimate the synonymous (Ks) and non-synonymous (Ka) substitution rates, as well as their ratio (Ka/ Ks).

### Genome comparison

To consider divergence in plastid genomes, identity across entire plastomes was visualized using the mVISTA viewer in the Shuffle-LAGAN mode [44] among 20 *Astragalus* accessions, with *Oxytropis bicolor* (accession number: MN255323) as the reference.

### Potential RNA editing sites prediction

The Predictive RNA Editor for Plants (PERP)-Cp web server (http://prep.unl.edu) [45] (with a cutoff value of 0.8), was used to predict potential RNA editing sites in Thirty-five protein coding genes of *Astragalus* species.

### Phylogenetic reconstruction

Seventy-five protein-coding genes were identified from 53 species within the IRLC and two outgroups [*Robinia pseudoacacia* L. and *Lotus japonicus* (Regel) K.Larsen]. This study obtained the whole plastid genomes of *A*. *iranicus*, *A*. *macropelmatus*, *A*. *mesoleios*, and *A*. *odoratus*, as well as other plastomes downloaded from GenBank (S1 Table). Maximum likelihood and Bayesian inference methods were used to analyze the concatenated data. Prior to performing maximum likelihood and Bayesian analyses, a general time reversible and gamma distribution (GTR + G) model was chosen using the MrModeltest2.2 [46] under Akaike Information Criteria (AIC) [47]. Maximum likelihood analyses were implemented with the online phylogenetic software W-IQ-TREE [48] available at http://iqtree.cibiv.univie.ac.at. Rapid bootstrap analyses (with 5000 replicates) were used to calculate node supports. MrBayes v.3.2 in the CIPRES [42] was used to perform Bayesian inference with the following parameters: Markov chain Monte Carlo (MCMC) simulations with four incrementally heated chains for 10,000,000 generations, starting from random trees and sampling one out of every 1,000 generations. The first 25% of the trees were considered to be burn-ins. The remaining trees were used to make a consensus tree with a 50% majority rule and to estimate posterior probabilities. Posterior probabilities (PP) greater than 0.95 were considered as main support for a clade.

## Results

### Characteristics of the newly sequenced *Astragalus* plastomes

**Fig 1:**
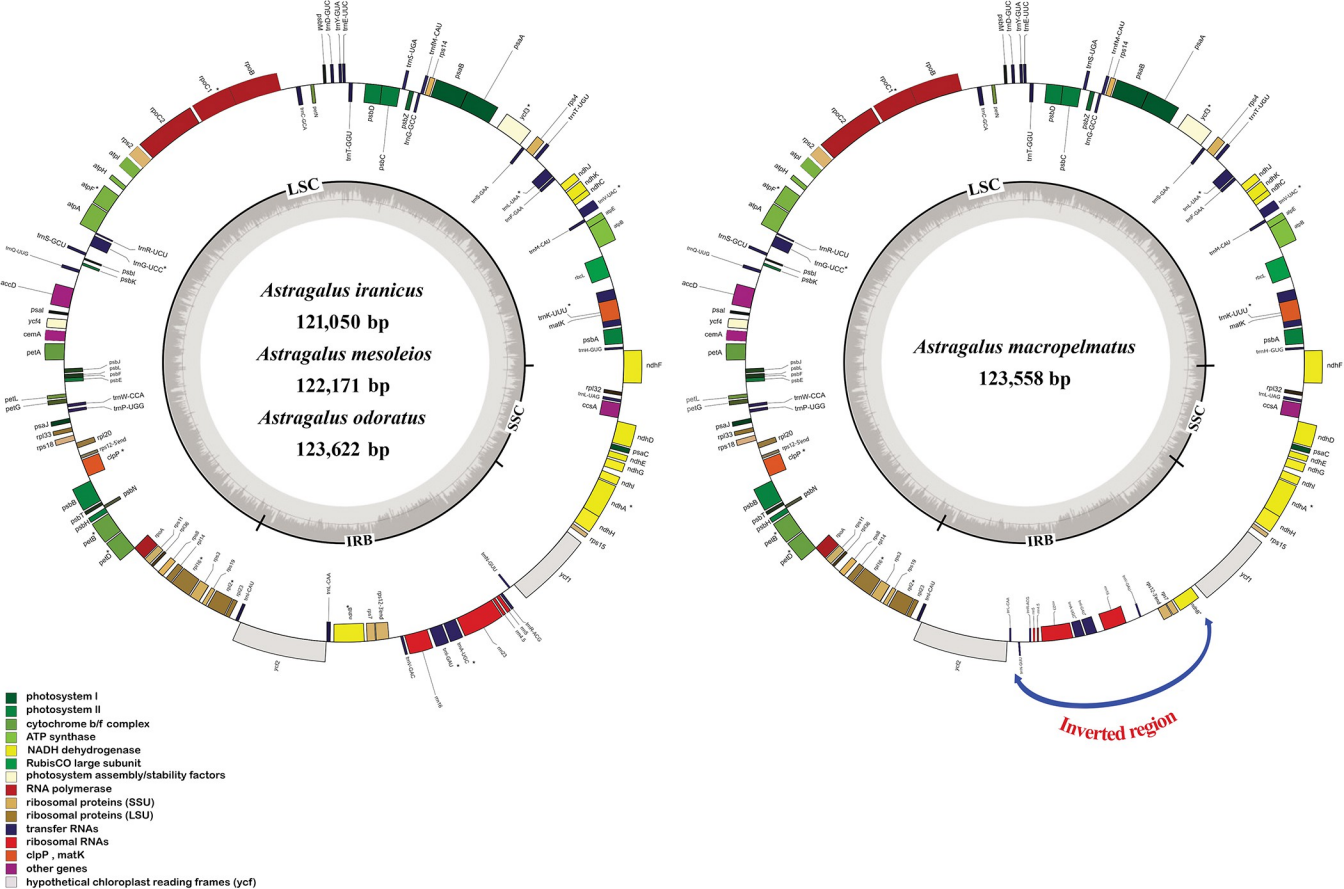
**A.** Circular gene map of three *Astragalus* species plastid genome. The genes draw inside the circle are transcribed in clockwise direction and genes on the outside the circle are transcribed in counterclockwise direction. Genes with different functions were demonstrated in different colors. The thick line inner circle shows the structure of the chloroplast: The large single copy (LSC), small single copy (SSC) and inverted repeat (IR) regions. Genes that have introns are marked with an asterisk. **B.** Annotated plastome of *Astragalus macropelmatus*. A 14-kb inversion is found in *A*. *macropelmatus* (*ndh*B ~ *trn*N(GUU)). Arrow indicates inverted location.

The Illumina miSeq-550 system produced 2,937,124 paired-end raw reads for *A*. *iranicus* species and 10,549,829 for *A*. *macropelmatus*. The plastid genomes assembled with Velvet and NOVOPlasty were the same. The lengths of the four newly sequenced whole chloroplast genomes ranged from 121050 to 123,622 bp (Table 1). All of the newly sequenced *Astragalus* chloroplast genomes showed the typical IRLC structure with having a single copy of the IR region. In this regard, the lack of *inf*A, *rps*16 and *rpl*22 genes and the first *clp*P intron in the plastid genomes of four *Astragalus* species are noted; these regions, found in other angiosperms, are absent from the plastomes of all the IRLC taxa [15, 20, 49]. There were 110 genes in the four *Astragalus* plastid genomes, including 76 protein-coding genes, 30 transfer RNA (tRNA) genes, and four ribosome RNA (rRNA) genes (Fig 1A, 1B, Table 2). The LSC (79,613–81,267 bp), SSC (12,614–13,758 bp), and IR (28,259–29,517 bp) regions, as well as 110 gene locations in the plastome are shown in Fig 1A, 1B. Similar genome structure and gene order were reported in newly sequenced *Astragalus* species, which is consistent with the plastomes of the *Astragalus* species studied so far. In four *Astragalus* species plastid genomes, 16 genes have one intron, whereas *ycf*3 has two introns (S2 Table). The *rps*12 gene is a trans-splicing gene with no introns at the 3’ end. The intron of *trn*K(UUU) is the largest (2,530–2,567 bp) encompassing the *mat*K gene, whereas the smallest intron (539–551 bp) belong to *trn*L(UAA). Although the overall gene content and arrangement within *Astragalus* plastid genomes is highly similar (Table 2), there are some structural differences in some species. *Astragalus macropelmatus* has an approximately 13-kb inversion (*ndh*B ~ *trn*N(GUU)) that placed *trn*N(GUU) next to *trn*L(CAA) and *ndh*B adjacent to *ycf*1 (Fig 1B). PCR and Sanger sequencing were used to confirm the presence of the inversion in the plasome of *A*. *macropelmatus*. Two diagnostic primer pairs were designed to confirm the presence of this inversion in *A*. *macropelmatus* and to screen other *Astragalus* species for the presence/absence of this 13-kb inversion. PCR amplification from primers in the *trn*L(CAA) and *ndh*B protein-coding regions was expected only in taxa without the inversion, whereas PCR amplification from primers in the *ycf*1 and *ndh*B genes was expected only in taxa with the inversion. In our study, Sanger sequencing results agree with the presence of 13-kb inversion in the *A*. *macropelmatus* chloroplast genome (accession numbers: LC764454 and LC764455). The overall guanine-cytosine (GC) content of the new sequenced *Astragalus* plastid genomes ranged from 34.1% to 34.4% (Table 2).

**Table 1:** Plastid genome information of selective *Astragalus* species and the four newly assembled *Astragalus* species.

Species	Size (bp)	LSC (bp)	GC (%) (LSC)	SSC (bp)	GC (%) (SSC)	IR (bp)	GC (%) (IR)	GC (%) Total
*A*. *arrectus*	122,721	80,493	33.4%	15,231	29.9%	26,997	38.7%	34.1%
*A*. *bhotanensis*	123,278	81,244	33.3%	13,671	30.0%	28,363	38.2%	34.0%
*A*. *calycosus*	122,244	80,001	33.6%	13,846	30.0%	28,397	38.3%	34.3%
*A*. *flexuosus*	123,578	80,920	33.3%	13,890	29.8%	28,768	37.9%	34.0%
*A*. *galactites*	126,117	83,955	33.1%	13,930	29.8%	28,232	38.5%	33.9%
*A*. *gypsodes*	122,194	80,329	33.5%	13,751	30.0%	28,114	38.5%	34.3%
*A*. *iranicus*	121,050	80,039	33.5%	12,626	31.1%	28,385	38.2%	34.4%
*A*. *laxmannii*	122,844	80,359	33.4%	13,949	29.8%	28,536	38.2%	34.1%
*A*. *macropelmatus*	123,558	80,283	33.5%	13,758	29.9%	29,517	38.1%	34.2%
*A*. *membranaceus*	123,623	81,045	33.3%	13,776	29.9%	28,802	38.1%	34.1%
*A*. *mesoleios*	122,171	80,247	33.4%	13,665	30.1%	28,259	38.3%	34.2%
*A*. *mollissimus*	122,511	80,318	33.5%	13,761	30.2%	28,432	38.4%	34.3%
*A*. *mongholicus*	123,582	80,986	33.4%	13,773	29.9%	28,823	38.1%	34.1%
*A*. *nakaianus*	123,633	81,053	33.3%	13,777	29.9%	28,803	38.1%	34.1%
*A*. *neglectus*	122,253	80,015	33.4%	14,806	29.8%	27,432	38.5%	34.1%
*A*. *nuttallianus*	122,840	80,659	33.6%	13,856	30.1%	28,325	38.5%	34.3%
*A*. *odoratus*	123,622	81,391	33.3%	13,685	30.0%	28,546	38.1%	34.1%
*A*. *pectinatus*	123,069	80,421	33.4%	13,867	29.8%	28,781	38.1%	34.1%
*A*. *scaberrimus*	123,492	80,856	33.3%	14,030	29.9%	28,606	38.2%	34.0%
*A*. *strictus*	122,796	80,760	33.4%	13,789	30.0%	28,247	38.5%	34.2%

LSC: Large Single Copy, SSC: Small Single Copy, IR: Inverted Repeat.

**Table 2:** Genes and functional genes classification in *A*. *iranicus*, *A*. *macropelmatus*, *A*. *mesoleios* and *A*. *odoratus* chloroplast genome.

Category of genes	Group of genes	Name of genes
Self-replication	Large subunit of ribosomal proteins	*rpl*14, *rpl*16*, *rpl*2*, *rpl*20, *rpl*23, *rpl*32, *rpl*33, *rpl*36
	Small subunit of ribosomal proteins	*rps*2, *rps*3, *rps*4, *rps*7, *rps*8, *rps*11, *rps*12*, *rps*14, *rps*15, *rps*18, *rps*19
	DNA-dependent RNA polymerase	*rpo*A, *rpo*B, *rpo*C1*, *rpo*C2
	Ribosomal RNA genes	*rrn*16S, *rrn*23S, *rrn* 4.5S, *rrn* 5S
	Transfer RNA genes	30 *trn* genes (5 contain an intron)
Genes for photosynthesis	Subunits of NADH-dehydrogenase	*ndh*A*, *ndh*B*, *ndh*C, *ndh*D, *ndh*E, *ndh*F, *ndh*G, *ndh*H, *ndh*I, *ndh*J, *ndh*K
	Subunits of photosystem I	*psa*A, *psa*B, *psa*C, *psa*I, *psa*J
	Subunits of photosystem II	*psb*A, *psb*B, *psb*C, *psb*D, *psb*E, *psb*F, *psb*H, *psb*I, *psb*J, *psb*K, *psb*L, *psb*M, *psb*N, *psb*T, *psb*Z
	Subunits of cytochrome b/f complex	*pet*A, *pet*B*, *pet*D*, *pet*G, *pet*L, *pet*N
	Subunits of ATP synthase	*atp*A, *atp*B, *atp*E, *atp*F*, *atp*H, *atp*I
	Subunit of rubisco	*rbc*L
Other genes	Maturase K	*mat*K
	Envelope membrane protein	*cem*A
	Subunit of Acetyl-CoA-carboxylase	*acc*D
	C-type cytochrome synthesis gene	*ccs*A
	Protease	*clp*P*
Genes of unkown function	Conserved hypothetical chloroplast open reading frames	*ycf*1, *ycf*2, *ycf*4, *ycf*3**

The number of asterisks after the gene names indicates the number of introns contained in the genes.

In the present study, other species of *Astragalus* whose plastomes have been sequenced to date were selected as representatives and their genome structure were compared (Table 1). Accordingly, all of the representative species of *Astragalus* have one copy of the IR. Some previous studies [28, 50, 51] have shown that *A*. *galactites* and *A*. *laxmannii* have two IR regions. In this study, two species were re-annotated and it was found that both species, like other taxa belonging to the IRLC, only have one IR region. There seems to be some rearrangements (i.e., increased repeat content) in the plastome of *A*. *galactites*. All plastid genomes of *Astragalus* species showed the typical structure of the IRLC composed of LSC (79,613 to 83,955), SSC (12,614 to 15,231) and one inverted repeat (26,997 to 29,517) regions (Table 1). The longest and the shortest plastome size belonged to the *A*. *galactites* (126,117 bp) and *A*. *iranicus* (121,050 bp), respectively (Table 1). Plastomes of the all *Astragalus* species are extremely conserved with respect to gene content and order. The GC contents of the LSC (33.1% to 33.6%) and SSC (29.8% to 31.1%) regions in studied species were lower than those of the IR (37.9% to 38.5%) regions. The highest guanine-cytosine (GC) content (34.4%) was found in the *A*. *iranicus* plastome, while the lowest (33.9%) was found in the *A*. *galactites* plastome (Table 1).

### Codon usage bias

We examined the codon distribution in the protein-coding regions of the four newly sequenced *Astragalus* species and compared them with other representative species of *Astragalus*. These protein-coding genes showed a total of 24,871 codons in *A*. *macropelmatus*, 24,736 codons in *A*. *odoratus*, 24,675 codons in *A*. *iranicus* and 24,718 codons in *A*. *mesoleios*. These codons belonged to 61 different types of codons, which encoded 20 amino acids. The most frequent amino acid was phenylalanine, and the most frequent codon was TTT. The codons of the amino acid Arginine were found to be the least abundant in the plastomes of four *Astragalus* species (S3 Table). Furthermore, only one codon was found to code the amino acids methionine (ATG) and tryptophan (TGC). The most prevalent start codon is the ATG codon. In four *Astragalus* species, however, we found ACG as the initiator codon in the *ndh*D gene.

The plastomes of *Astragalus* representative species were examined for codon usage frequency based on protein-coding gene sequences and relative synonymous codon usage (RSCU). The RSCU value, which represents non-uniformity in codon usage, was calculated by dividing the actual observed values of the codon by the theoretical expectations. A value larger than 1.0 indicates that a positive codon usage bias exists for a codon, while a value less than 1.0 indicates that a negative codon usage bias exists for a codon. When the RSCU value is 1.0, there is no codon usage bias [52]. The total number of codons in the *Astragalus* species varies from 21,470 codons in *A*. *arrectus* (as the smallest codon number) to 24,871 codons in *A*. *macropelmatus*. Methionine (AUG) and tryptophan (UGG) with RSCU = 1 had no bias. Meanwhile, the greatest RSCU value was recorded for UUA and AGA that encode leucine and serine amino acids respectively and the lowest belonged CUG that encode leucine (S4 Table). Therefore, UUA and AGA were positively biased while CUG was negatively biased. Furthermore, leucine exhibited A or T (U) bias in all synonymous codons (UUA, UUG, CUU, CUC, CUA, and CUG). Except for the UUG codon, all biased relative synonymous codons (RSCU > 1) ended with an A or U. Furthermore, the majority of codons ending in C or G have an RSCU value less than one (S4 Table). High A/U preference, a common phenomenon in higher plant plastomes [53], was observed in the third codon of *Astragalus* species. The RSCU values of the plastomes are a useful source of evolutionary traits resulting from selection and mutation which are essential for investigating organism evolution [54, 55].

### Repeat structure and simple sequence repeats

**Fig 2:**
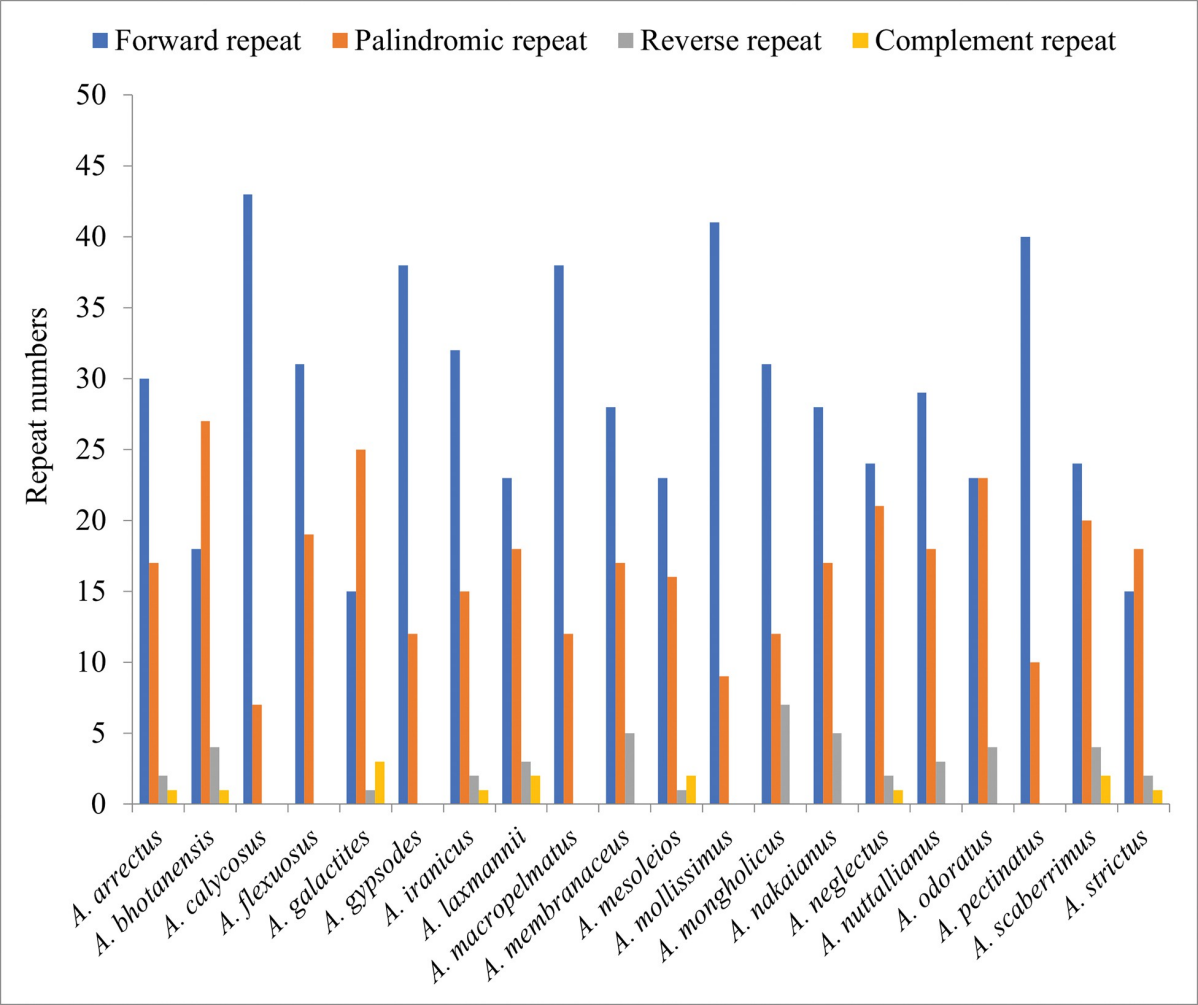
Repeated sequences analysis in the *Astragalus* plastomes.

Repetitive motifs play an important role in computing repeat, deletion, and rearrangement events in the chloroplast genome [55]. Repeat analysis of four newly sequenced *Astragalus* plastomes detected 42 (*A*. *mesoleios*) to 50 (*A*. *iranicus*, *A*. *macropelmatus*, *A*. *odratus*) repeat structures ranging in length from 30 to 434 bp. In this study, *A*. *iranicus* reported 50 long repeats comprised of 32 forward (F), 15 palindromic (P), two reverse (R) and one complement (C) repeats, *A*. *macropelmatus* recorded 50 long repeats composed of 38 F and 12 P repeats, *A*. *mesoleios* showed 42 long repeats included 23 F, 16 P, two R and one C repeats and *A*. *odoratus* demonstrated 50 long repeats consisted of equal numbers of F and P (23) and four R repeats (S5 Table). The most abundant type of repeats was the forward, with lengths ranging from 23 to 38 bp in all four species (Fig 2). These repeats could be useful in investigating the population genetic of these four *Astragalus* species.

In the plastomes of the representative species of *Astragalus*, we detected four different types of dispersed repeats (forward, reverse, complementary, and palindromic). The number of repetitive structures in the *Astragalus* plastomes ranges from 36 (*A*. *strictus*) to 50 (most species) pairs (Table 3). The most common repeats were found to be forward types, ranging from 15 (*A*. *galactites*) to 43 (*A*. *calycosus*), followed by palindromic repeats which range from 7 (*A*. *calycosus*) to 27 (*A*. *bhotanensis*) (Fig 2). Forward types were also the longest repeats, with the length of 434 bp found in the intergenic spacer (IGS) of the large single copy (LSC) region between *trn*Q(UUG) and *acc*D, was detected in the *A*. *iranicus* and the shortest repeat with lengths of 30 bp was also forward type and was detected in most *Astragalus* species (S5 Table).

**Table 3:** Statistical information of repeat types within *Astragalus* species.

Taxon	Forward	Palindromic	Reverse	Complement	Total number
*A*. *arrectus*	30	17	2	1	50
*A*. *bhotanensis*	18	27	4	1	50
*A*. *calycosus*	43	7	0	0	50
*A*. *flexuosus*	31	19	0	0	50
*A*. *galactites*	15	25	1	3	44
*A*. *gypsodes*	38	12	0	0	50
*A*. *iranicus*	32	15	2	1	50
*A*. *laxmannii*	23	18	3	2	46
*A*. *macropelmatus*	38	12	0	0	50
*A*. *membranaceus*	28	17	5	0	50
*A*. *mesoleios*	23	16	1	2	42
*A*. *mollissimus*	41	9	0	0	50
*A*. *mongholicus*	31	12	7	0	50
*A*. *nakaianus*	28	17	5	0	50
*A*. *neglectus*	24	21	2	1	48
*A*. *nuttallianus*	29	18	3	0	50
*A*. *odoratus*	23	23	4	0	50
*A*. *pectinatus*	40	10	0	0	50
*A*. *scaberrimus*	24	20	4	2	50
*A*. *strictus*	15	18	2	1	36

**Fig 3:**
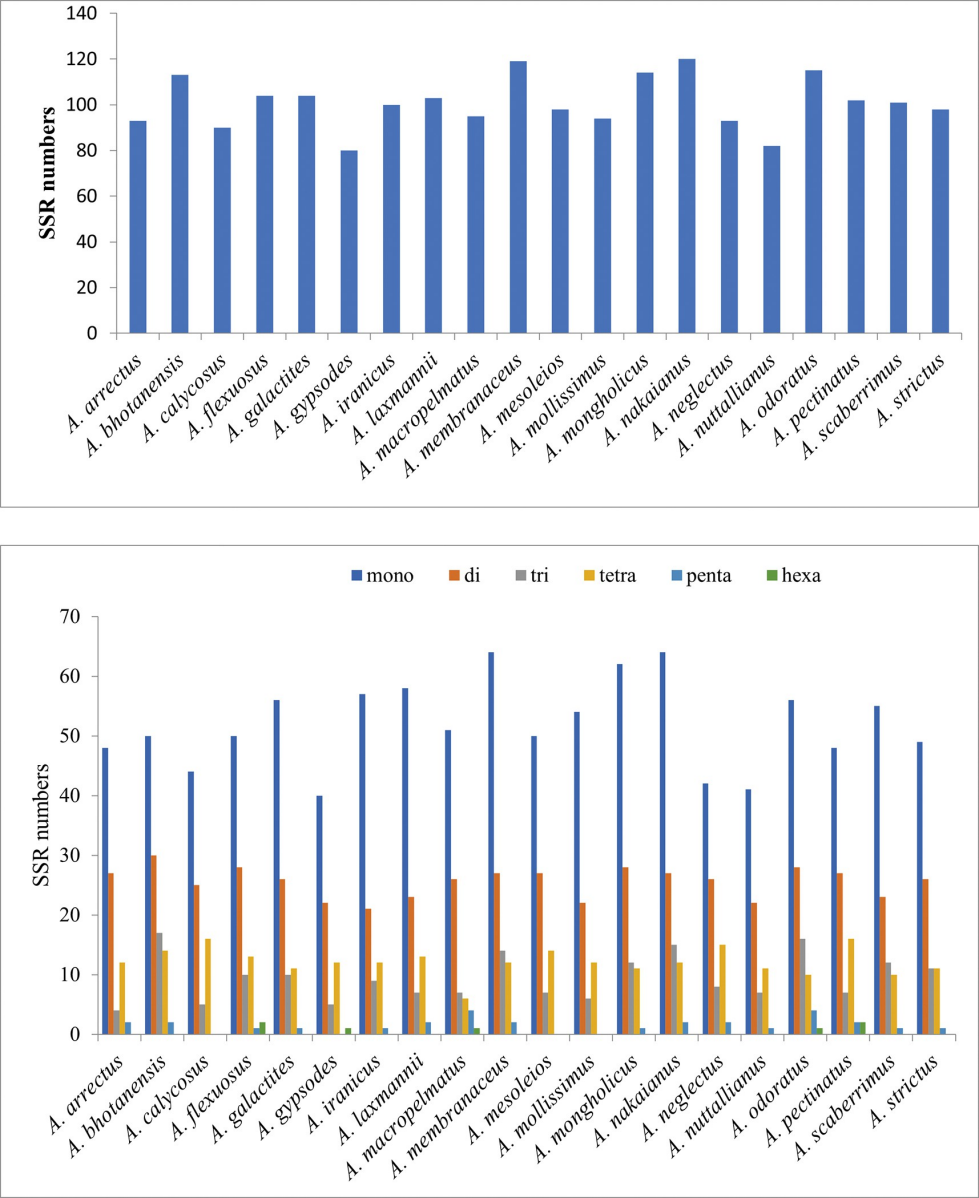
Analysis of simple sequence repeats (SSRs) in the *Astragalus* plastomes. (A) The number of SSRs found in the *Astragalus* plastomes; (B) The number of SSR types identified in the *Astragalus* plastomes.

SSRs (Simple Sequence Repeats), also known as microsatellite sequences, are uniparentally inherited, short, tandemly repeated DNA motifs of 1–6 nucleotides that are widely distributed throughout the plastid genome [55, 56]. Using MISA, we identified the occurrence and types of cpSSRs in the plastomes of four *Astragalus* species. Five kinds of SSRs were found: mononucleotides, dinucleotides, trinucleotides, tetranucleotides, pentanucleotides and hexanucleotides. A total of 100 SSRs were identified in *A*. *iranicus* composed of 57 (57%) mono-repeats, 21 (21%) di-repeats, nine (9%) tri-repeats, 12 (12%) tetra-repeats and one (1%) penta-repeats (Fig 3A, S6 Table). No hexanucleotide SSRs existed in the *A*. *iranicus* species (Fig 3B, S6 Table). *A*. *macropelmatus* cp genome had 95 SSRs composed of 51 (53.68%) mono-repeats, 26 (27.36%) di-repeats, seven (7.36%) tri-repeats, six (6.31%) tetra-repeats, four (4.21%) penta-repeats and one (1.05%) hexa-repeats (Fig 3B, S6 Table). *A*. *mesoleios* with 98 SSRs was included 50 (51.02%) mono-repeats, 27 (27.55%) di-repeats, seven (7.14%) tri-repeats, 14 (14.28%) tetra-repeats. No penta- or hexa-nucleotide SSRs were found in *A*. *mesoleios*. The number of cpSSRs in *A*. *odoratus* was 115 consisted of 56 (48.69%) mono-repeats, 28 (24.34%) di-repeats, 16 (13.91%) tri-repeats, 10 (8.69%) tetra-repeats, four (3.47%) penta-repeats and one (0.86%) hexa-repeats (Fig 3B, S6 Table).

The SSR distribution patterns in plastid genome of the *Astragalus* species included in this study were all similar. In these *Astragalus* species, the total number of SSRs ranged from 120 (*A*. *nakaianus*) to 80 (*A*. *gypsodes*) (Fig 3A, Table 4). In each of the 20 representative species, mono-, di-, tri-, tetra-, penta-, and hexanucleotide SSRs were observed, with the average percentages of mono-, di-, tri-, tetranucleotide SSRs were 51.48%, 25.32%, 9.36%, and 12.04%, respectively. In all cp genomes, we found penta- and hexanucleotide motifs to be very rare (Fig 3B, Table 4). The majority of SSRs were AT-rich and rarely contain CG. These results are consistent with the observed Leguminosae species [28, 29, 57]. These SSR loci are located primarily in the LSC region as compared to the SSC and the IR regions.

**Table 4:** Statistical data of simple sequences repeats (SSRs) within *Astragalus* species.

Taxon	mono-	di-	tri-	tetra-	penta-	hexa-	Total number
*A*. *arrectus*	48	27	4	12	2	0	93
*A*. *bhotanensis*	50	30	17	14	2	0	113
*A*. *calycosus*	44	25	5	16	0	0	90
*A*. *flexuosus*	50	28	10	13	1	2	104
*A*. *galactites*	56	26	10	11	1	0	104
*A*. *gypsodes*	40	22	5	12	0	1	80
*A*. *iranicus*	57	21	9	12	1	0	100
*A*. *laxmannii*	58	23	7	13	2	0	103
*A*. *macropelmatus*	51	26	7	6	4	1	95
*A*. *membranaceus*	64	27	14	12	2	0	119
*A*. *mesoleios*	50	27	7	14	0	0	98
*A*. *mollissimus*	54	22	6	12	0	0	94
*A*. *mongholicus*	62	28	12	11	1	0	114
*A*. *nakaianus*	64	27	15	12	2	0	120
*A*. *neglectus*	42	26	8	15	2	0	93
*A*. *nuttallianus*	41	22	7	11	1	0	82
*A*. *odoratus*	56	28	16	10	4	1	115
*A*. *pectinatus*	48	27	7	16	2	2	102
*A*. *scaberrimus*	55	23	12	10	1	0	101
*A*. *strictus*	49	26	11	11	1	0	98

### Nucleotide substitution rates

Using DnaSP v.6.12, we compared the non-synonymous (Ka) to synonymous (Ks) substitution ratio (denoted as *ω*) for 76 protein-coding genes between the newly sequenced taxa and other selective *Astragalus* species (S7 Table). The Ka/Ks ratio is frequently used to assess the natural selection pressure and evolution rate of nucleotides, which is an important marker in investigation of species evolution. Accordingly, *ω* is an indicator of adaptive evolution or positive selection. Neutral evolution is signified by a *ω* value of 1, *ω <* 1 indicates purifying (negative) selection, and *ω >* 1 indicates that the gene is under the positive (adaptive) selection [58]. Most of the 75 protein-coding genes had a low *ω* value (less than 0.9), inferring that most of these genes were affected by purifying selection during the evolution. The highest nonsynonymous and synonymous rates were found in the *clp*P gene, which encodes a caseinolytic peptidase participating throughout plastid protein metabolism (Ka = 0.107069, Ks = 0.078252). In this study, the *ω* value was estimated to be 0 for seven genes in the LSC/IR region (*rps*12, *psb*L, *psb*T, *psb*N, *psb*H, *pet*N, *pet*G) (S7 Table). This occurred as a result of the Ka or Ks being 0 or extremely low, thus *ω* could not be calculated [57]. The most rapidly evolving genes in *Astragalus* species which indicates positive selection, were *rps*11, *rps*15, *acc*D, *clp*P and *ycf*1. These results suggested that the chloroplast genes in different Astragalus species may have been underwent to different selection pressures.

### Genomic divergence

To assess genomic divergence, mVISTA sequence identity analysis [44] was performed on the 15 *Astragalus* species, *Oxytropis bicolor* was used as a reference. We observed lower divergence in the IR region and protein-coding sequences than in non-coding regions which also occurred in almost higher plants (S1 Fig). High nucleotide variations were found among *Astragalus* species for the protein-coding genes *ycf*1, *ycf*2, *acc*D and *clp*P as well as intergenic regions such as *trn*Q(UUG)–*acc*D, *rps*7 –*trn*V(GAC) and *trn*R(ACG)–*trn*N(GUU). These divergence hotspot regions provided valuable data for the development of molecular markers for *Astragalus* species identification, population genetics and phylogenetic analyses.

**Fig 4:**
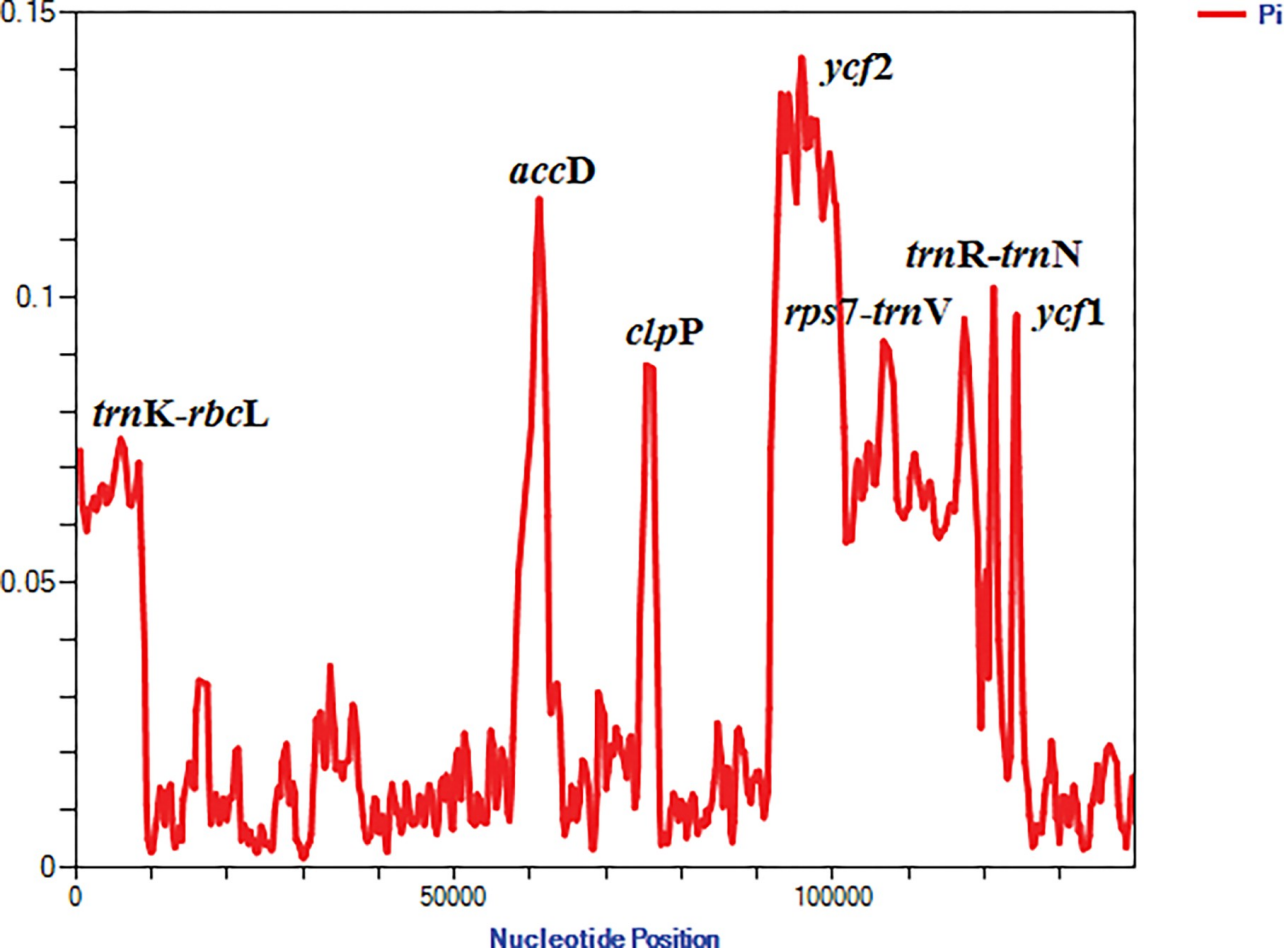
Nucleotide variability (%) values among the *Astragalus* species (using for coding regions). Window length: 800 bp; step size: 200 bp. X-axis: Position of the midpoint of a window. Y-axis: Nucleotide diversity of each window.

The same regions were found in the plastid genomes of the *Astragalus* species using sliding window analysis. Nucleotide variability (Pi) was calculated using the DnaSP software to estimate the sequence divergence level. The average value of Pi among the 20 chloroplast genomes of *Astragalus* species was calculated to be 0.02933 (Fig 4). Seven regions (*acc*D, *clp*P, *ycf*1 and *ycf*2 as protein-coding regions and *trn*K(UUU)—*rbc*L, *rps*7—*trn*V(GAC) and *trn*R(ACG)—*trn*N(GUU) as intergenic regions) demonstrated high nucleotide variability, with Pi values > 0.05 (Fig 4) and were located in the LSC and IR regions. mVISTA also obtained similar results. These are rapid evolutionary change regions in the chloroplast genomes that may be useful for the population genetics, phylogenetic reconstruction and development of molecular markers.

### Prediction of RNA editing sites

RNA editing is a post-transcriptional modification process in higher plant chloroplasts that converts cytidine (C) to uridine (U) or U to C at peculiar sites within RNA. Prep-CP prediction tool was used to predict RNA editing sites of plastid genes in 20 species of *Astragalus* (S8 Table). In total, an average of 40 editing sites were exist in 15–17 plastid protein-coding genes for each species, all of which were C-to-U (cytidine to uridine) conversions (S8 Table). *ndh* genes had the most editing sites, with a total of 18 for each species, followed by the *rpo*B gene, which had seven editing sites. Three editing sites were detected in *acc*D gene. There were also two editing sites in *ccs*A, *mat*K and *rpo*C1 genes. There was only one editing site found for each of the remaining genes. Our study showed that in *Astragalus* species, the probability of editing *ndh* genes is higher than other genes at mRNA level.

### Phylogenetic relationship analysis

**Fig 5:**
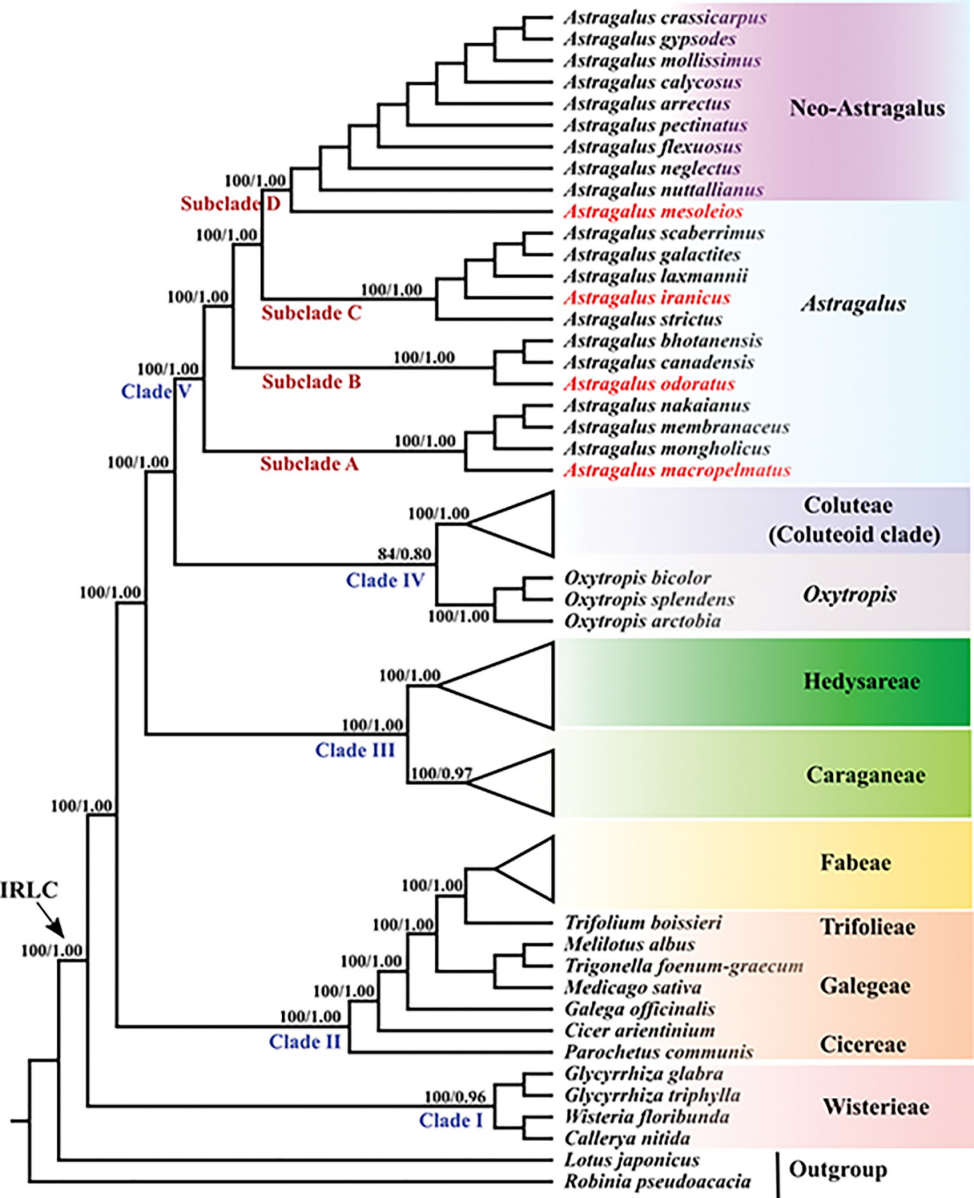
Maximum-likelihood phylogenetic tree inferred from 53 chloroplast genomes of the IRLC. The position of *A*. *iranicus*, *A*. *macropelmatus*, *A*. *mesoleios* and *A*. *odoratus* is shown in red. Numbers above branches are likelihood values and posterior probabilities, respectively.

To identify the phylogenetic positions and relationships of the newly sequenced *Astragalus* species (*A*. *iranicus*, *A*. *macropelmatus*, *A*. *mesoleios* and *A*. *odoratus*), Bayesian inference (BI) and maximum likelihood (ML) methods of phylogenetic analysis were implemented based on 75 protein-coding region datasets from 49 plant taxa from different tribes of the IRLC, with *Lotus japonicus* and *Robinia peudoacacia* used as outgroups. The phylogenetic topologies of the ML and BI trees were similar, with high support values (Fig 5). Therefore, only the ML tree is shown in Fig 5. The phylogenetic tree can be defined into five clades: clade I comprises tribes Wisterieae and Glycyrrhizeae which were sister to the entire of the IRLC, clade II contains tribes Cicereae, Trifolieae and Fabeae and genus *Galega* (from paraphyletic tribe Galegeae), clade III consists of monophyletic tribes Caraganeae and Hedysareae, clade IV includes tribe Coluteae and genus *Oxytropis* and clade V comprises monophyletic genus *Astragalus*. 22 species of *Astragalus* form a well-supported clade that include four subclades. All the *Astragalus* subclades were fully supported in the phylogenetic tree. *A*. *macropelmatus* as basal branch along with *A*. *mongholicus*, *A*. *membranaceus* and *A*. *nakaianus* formed subclade A. *A*. *odoratus*, *A*. *canadensis* and *A*. *bhotanensis* constituted the subclade B. Next subclade (C) comprised *A*. *iranicus*, *A*. *strictus*, *A*. *laxmannii*, *A*. *galactites* and *A*. *scaberrimus*. In subclade D, *A*. *mesoleios* was the first diverging lineage and was sister to the remaining species, which are Neo-Astragalus species.

## Discussion

### Plastid genome organization and gene content

In this study, we used the Illumina platform to sequence four *Astragalus* chloroplast genomes (*A*. *iranicus*, *A*. *macropelmatus*, *A*. *mesoleios*, and *A*. *odoratus*) and compared them to the other published plastomes of the same genus available on GenBank. Our assembly results showed that the plastome structures of the four species were similar to the other *Astragalus* species and the lengths of their plastid genomes ranged from 121,050 bp to 123,622 bp (Fig 1A). All the plastomes, like other members of the IRLC, have lost single copy of the IR [10, 59]. Although the overall genome structure and gene content within the four newly sequenced *Astragalus* is highly similar and conserved, a structural change is detected in *A*. *macropelmatus* plastome. The cp genome of *A*. *macropelmatus* has experienced a distinctive 13-Kbp inversion in the IR region that has not been observed in any of the legume plastomes sequenced to date (Fig 1B). In all the legumes, *ndh*B gene is located next to the *trn*L(CAA) gene, while in *A*. *macropelmatus*, *ndh*B gene has been rearranged, inverted and located next to the *ycf*1 gene. The structure of the plastid genome is highly conserved in most photosynthetic angiosperm lineages, except Campanulaceae, Fabaceae and Geraniaceae that represent extensive rearrangements [20, 60]. The most significant rearrangements within the Fabaceae is inversion that has been occurred in several lineages such as in most of the papilionoids with 50-kb inversion [12, 15], in *Tylosema esculentum*, one of the basal most legumes, with a unique inverted region of six genes *rbc*L, *acc*D, *psa*I, *ycf*4, *cem*A and *pet*A [61], in plastome of *Onobrychis viciifolia* with *ycf*2/*trn*I(CAU)/*trn*L(CAA) inverted genes [38] and in plastid genome of *Gueldenstaedtia verna* with four inverted regions (*trn*K–*psb*K, *acc*D–*rpl*23, *rps*15 –*trn*L, *trn*L–*trn*I) [62]. Furthermore, in the recent study of Charboneau et al. (2021) [27], several inversions were shown in the plastome of four species of the New World *Astragalus*. *Astragalus calycosus* has ~7-kb inversion between *rbc*L and *trn*H(GUG); there is a ~40-kb inversion between *trn*Q(UUG) and *trn*T(UGU) in *A*. *mollissimus;* and *A*. *flexuosus* and *A*. *neglectus* have experienced ~7-kb inversion between *trn*L(CAA) and *trn*I(CAU). Various mechanisms are involved in the occurrence of inversion in the genome, one of the most significant of which is the existence of the short-inverted repeats adjacent the endpoints of these regions [27, 63]. High level of repeat content and count (specially near the ends of inversions) has been found in taxa with high levels of genomic rearrangements [6, 18, 27] but there may or may not be a causative connection between the two correlated variables. Due to relative rarity and ease of determination of inversions, these regions are extremely valuable and useful in phylogenetic studies [59]. Other mechanism for the occurrence of inversions is intramolecular recombination between repeat elements and tRNA genes [27, 38].

Repetitive elements can also cause other genomic rearrangements such as gene/intron loss, pseudogenization and a second independent IR region gain (for the IRLC taxa). IR reemergence has been reported in some IRLC taxa in the recent studies (e.g. *Medicago minima* [19, 64] and *Melilotus dentata* [21]). In this regard, IR reemergence in two *Astragalus* species (*A*. *galactites* and *A*. *laxmannii*) has been erroneously reported [28, 50, 51]. In this study, the complete plastid genomes of the both species were re-annotated and it was found that, like other members of the IRLC, they have only one IR region.

In addition to the genus *Astragalus*, the plastome of some other genera of the IRLC have also undergone different genomic rearrangements. For example, the lack of *acc*D gene in some species of *Trifolium* [18, 20, 65], the absence of the second *clp*P intron (except intron 1 of *clp*P gene which is absent at the base of the IRLC) [15]) in *Glycyrrhiza glabra*, *G*. *lepidota*, *Tibetia liangshanensis* and seven species of Neo-Astragalus [20, 27, 66] and loss of *rpl*23, *rpl*33 and *ycf*4 genes in some *Lathyrus*, *Pisum* and *Vicia* species [16, 17, 29]. The occurrence of numerous rearrangements in the plastid genome of the IRLC taxa has made it a great model for studying plastome evolution. The occurrence of different rearrangements in the IRLC might be consequence of presence of many tandemly repeated sequences, the lack of IR and variability in IR region size [19, 66, 67].

The plastomes among *Astragalus* species were identical in guanine-cytosine content, but the GC% in the LSC and SSC regions were remarkably lower than IR region because of the presence of rRNA genes (*rrn*23, *rrn*16, *rrn*5, *rrn*4.5) with high GC content (50%-56.4%) [38, 57, 68]. GC content may be the most significant factor related to the phenomenon of codon usage bias among different organisms. Relative synonymous codon usage (RSCU) patterns are similar among *Astragalus* species and could provide useful reference for phylogenetic relationship analysis [53, 54].

Repeat sequences play important roles in the evolution and rearrangements of the plastome and can be used to develop genetic markers for population and phylogenetic studies [53]. Four types of repeats were found in plastid genome of *Astragalus* species using REPuter software. In the majority of the studied *Astragalus* species, forward dispersed repeats were found to be the most abundant, followed by palindromic and reverse repeats, and the least complement. Moreover, repeat sequences were mostly dispersed in non-coding regions of the *Astragalus* species. The presence of these repeat sequences indicates the loci which could be significant hotspots for plastid genome reconfiguration [38, 66]. In the studied *Astragalus* species, the most abundant observed motifs were mononucleotides and A/T repeats were the most frequent but no G/C motif found in their cp genomes. Strong A/T preference in SSR loci has been observed in many legume [38, 57, 68, 69] and non-legume [70, 71] species which may contribute to the bias in base composition [68]. SSRs were distributed across the plastome, with the highest frequency in the LSC region which may be related to the lack of single copy of the IR region in the IRLC taxa. SSRs can exhibit high genetic polymorphism and mutation rates and are frequently used for the development of molecular markers and play a crucial role in the recombination and rearrangement of genome, population genetics, gene mapping and identification of species [72].

Highly variable DNA markers are useful for identifying closely related species and provide abundant information for broad-scale phylogenetic analyses. In this study based on mVISTA and sliding window analysis, *acc*D, *clp*P, *ycf*1 and *ycf*2 as protein-coding regions and *trn*K(UUU)- *rbc*L, *rps*7- *trn*V(GAC) and *trn*R (ACG)- *trn*N(GUU) as intergenic regions, which showed some extent divergence, were detected with higher Pi values (Fig 4) and have potential to be used as DNA markers. Some of these regions have been used as molecular markers in previous *Astragalus* phylogenetics analyses [73–75]. The *ycf*1 is a more variable gene than *mat*K and is suitable for lower taxonomic levels in DNA barcoding and molecular systematics [3, 76], as well as, the *clp*P gene codes a caseinolytic peptidase located in the LSC region and demonstrated accelerated mutation in the IRLC [75]. Further studies are needed to assess whether these variable regions can be served in *Astragalus* phylogenetic analyses or use as great candidate markers for species authentication and population genetic.

### Plastid RNA editing prediction and positive selection analysis

RNA editing is a type of post transcriptional modification, which involves the insertion, deletion or conversion of cytidine (C) to uridine (U) nucleic acid bases in the chloroplasts of higher plants [77]. The present study found that the *ndh* genes had the most editing sites in the plastid genomes of *Astragalus* species. Also, the *ndh* group genes have shown the most chloroplast editing sites in flowering plants [77, 78]. The plastid *ndh* genes, which encode components of the thylakoid NDH complex, have either been lost or pseudogenized in various species of algae, bryophytes, pteridophytes, gymnosperms, angiosperms [78–80]. Some studies have mentioned that the products of the *ndh* genes might be unessential for plants growth under normal conditions [77]. RNA editing is crucial for the function of the NDH protein complex as well as for improving plant photosynthesis under adverse conditions [77].

We estimated the Ka/Ks for each gene in DnaSP v.6.12 to assess the selective pressure on protein-coding sequences on *Astragalus* plastomes. The Ka/Ks ratio is a common method for studying adaptive evolution or positive selection in plant species. In our evaluation, the Ka/Ks ratio reveals positive selection for *rps*11, *rps*15, *acc*D, *clp*P and *ycf*1 genes in the plastid genomes of the studied *Astragalus* species. The *rps* gene family are involved in self-replication, *acc*D gene encodes the beta-carboxyl transferase subunit of acetyl-CoA carboxylase which is necessary for plant development, *clp*P gene, as mentioned earlier, codes a caseinolytic peptidase and contains two introns (Intron 1 of *clp*P gene has been lost across the IRLC and also the second *clp*P intron was absent in some Neo-Astragalus, *Glycyrrhiza* and *Tibetia* species [27, 66]) and *ycf*1 gene encodes a protein with approximately 1,800 amino acids and is essential for plant viability [76]. In a study [28], it was, however, shown that *cem*A and *rpl*33 were underwent positive selection in *Astragalus* species. There are some regions with accelerated mutation rates in the plastome of legumes and in particular IRLC taxa which have been undergone adaptive evolution, including *rps*16-*acc*D-*psa*I-*ycf*4-*cem*A region. In this region, *rps*16 gene was lost across the IRLC [16, 59], *ycf*4 gene shows positive selection in some taxa of the tribe Fabeae (*Lathyrus*, *Pisum* and *Vavilovia*) [16, 17] and *acc*D gene absent in the *Trifolium* subgen. *Trifolium* [18, 20, 65].

### Phylogenetic implications

In our study, the chloroplast-based *Astragalus* phylogenomics was strongly supported and consistent with previous studies [27, 28, 81, 82]. In accordance with previous studies, IRLC is monophyletic and consists of several tribes/lineages including Wisterieae, Glycyrrhizeae, *Galega* (Galegeae), Cicereae, Trifolieae, Fabeae, Caraganeae, Hedysareae, Coluteoid clade and genera *Astragalus* and *Oxytropis* [12, 28, 81, 82]. In the present study, four newly sequenced *Astragalus* species (*A*. *iranicus*, *A*. *macropelmatus*, *A*. *mesoleios* and *A*. *odoratus*) along with other selected species of *Astragalus* from GenBank, form a monophyletic clade. In many previous studies [12, 83], the genus *Oxytropis* was retrieved as the sister to *Astragalus*, but in recent plastid DNA-based phylogenetic analyses [28, 38, 81, 82] in agreement with the present study, *Oxytropis* united with Coluteoid clade (tribe Coluteae) which, in turn, closely related to *Astragalus*. Our results showed that each of the four newly sequenced species of *Astragalus* (*A*. *iranicus*, *A*. *macropelmatus*, *A*. *mesoleios* and *A*. *odoratus*) were placed in their respective clade, as in previous studies [83, 84].

The results of our phylogenetic analysis for *Astragalus* species imply that complete plastid genome database can be powerful resource to construct relationships among species of this genus. The rapid development of plastome sequencing technologies has the potential to provide useful genomic information for reconstructing phylogenetic relationships at lower and higher taxonomic levels.

## Conclusions

We sequenced, assembled and compared the plastid genomes of four *Astragalus* species in this study (*A*. *iranicus*, *A*. *macropelmatus*, *A*. *mesoleios* and *A*. *odoratus*). All these species belong to the IRLC and tribe Galegeae. The organization and gene contents of the *Astragalus* plastomes were detected to be well conserved, however, the *A*. *macropelmatus* plastome showed a unique inversion (13-kb) in the IR region. We also obtained such comprehensive molecular information as codon usage, distribution of SSRs and repeat sequences, prediction of RNA editing, detection of hotspot regions and phylogenomic analysis. In addition, seven hypervariable regions (*acc*D, *clp*P, *ycf*1 and *ycf*2 as protein-coding regions and *trn*K(UUU)- *rbc*L, *rps*7- *trn*V(GAC) and *trn*R(ACG)- *trn*N(GUU) as intergenic regions) were detected, which might be used as molecular markers for genus/species identification. Our findings increase the data on the plastomes of *Astragalus* and provide useful resource for future research on population genetics, molecular phylogeny and evolution of *Astragalus*.

## Supporting information

S1 Table Accession number and sampled chloroplast genomes obtained from GenBank. (DOCX)

S2 Table Genes with intron in the *Astragalus* plastid genomes, including the exon and intron length. (DOCX)

S3 Table Codon usage in the *Astragalus* plastid genomes. (DOCX)

S4 Table Putative preferred codons in the *Astragalus* plastid genomes. (XLSX)

S5 Table Forward, reverse and palindromic repeat sequences in the *Astragalus* plastid genomes. (DOCX)

S6 Table SSRs showing in the plastomes of *Astragalus* species. (DOCX)

S7 Table The Ka, Ks and Ka/Ks ratio of *Astragalus* species chloroplast genomes for individual genes and regions. (DOCX)

S8 Table Prediction of RNA editing sites in chloroplast genes of *Astragalus* species. (DOCX)

S1 Fig Sequence alignment plot comparing 15 plastid genomes of *Astragalus* species with *O*. *bicolor* as a reference. Genome regions are color coded as protein coding, rRNA coding, tRNA coding, or conserved noncoding sequences. The vertical scale indicates the percentage identity, ranging from 50% to 100%.
(PDF)
