# Supplementary material for: Characterization and comparative analysis of the complete plastid genomes of four Astragalus species
Source: PLoS One. 2023 May 23;18(5):e0286083. doi: 10.1371/journal.pone.0286083 (PMC10204964; doi:10.1371/journal.pone.0286083)
Supplement: S1 Table — (DOCX) [file pone.0286083.s001.docx]

**S1 Table**. Accession number and sampled chloroplast genomes obtained from GenBank.

| **Species** | **Accession** | **Genome Size** |
| --- | --- | --- |
| **IRLC** | | |
| *Alhagi sparsifolia* | MT571455 | 123,233 bp |
| *Astragalus arrectus* | MZ923740 | 122,721 bp |
| *Astragalus mongholicus* | KU666554 | 123,582 bp |
| *Astragalus bhotanensis* | MN709865 | 123,278 bp |
| *Astragalus calycosus* | MZ923743 | 122,244 bp |
| *Astragalus canadensis* | MT579962 | 123,544 bp |
| *Astragalus crassicarpus* | KP126865 | 61000 bp |
| *Astragalus flexuosus* | MZ901207 | 123,578 bp |
| *Astragalus galactites* | MZ504977 | 126,117 bp |
| *Astragalus gypsodes* | MZ923745 | 122,194 bp |
| *Astragalus iranicus* | LC764834 | 121,050 bp |
| *Astragalus laxmannii* | MT786136 | 122,844 bp |
| *Astragalus macropelmatus* | LC764835 | 123,558 bp |
| *Astragalus membranaceus* | KX255662 | 123,623 bp |
| *Astragalus mesoleios* | LC764836 | 122,171 bp |
| *Astragalus mollissimus* | MZ901208 | 122,511 bp |
| *Astragalus nakaianus* | KR296789 | 123,633 bp |
| *Astragalus neglectus* | MZ923749 | 122,253 bp |
| *Astragalus nuttallianus* | MZ923750 | 122,840 bp |
| *Astragalus odoratus* | LC764837 | 123,622 bp |
| *Astragalus pectinatus* | MZ923753 | 123,069 bp |
| *Astragalus scaberrimus* | MW654102 | 123,492 bp |
| *Astragalus strictus* | MT120746 | 122,796 bp |
| *Callerya nitida* | MT120748 | 132,319 bp |
| *Caragana korshinskii* | KX289923 | 129,331 bp |
| *Caragana microphylla* | KX289922 | 130,029 bp |
| *Carmichaelia australis* | MF597719 | 122,805 bp |
| *Cicer arietinum* | EU835853 | 125,319 bp |
| *Galega officinalis* | MT506239 | 125,086 bp |
| *Glycyrrhiza glabra* | KF201590 | 127,943 bp |
| *Glycyrrhiza triphylla* | MT120806 | 127,735 bp |
| *Halimodendron halodendron* | MW349012 | 129,342 bp |
| *Hedysarum semenovii* | MN709827 | 123,407 bp |
| *Hedysarum taipeicum* | MK426698 | 126,699 bp |
| *Lathyrus sativus* | HM029371 | 121,020 bp |
| *Lens culinaris* | KF186232 | 122,967 bp |
| *Lessertia frutescens* | MF286764 | 122,700 bp |
| *Medicago sativa* | MK460489 | 125,330 bp |
| *Melilotus albus* | MH191352 | 127,205 bp |
| *Onobrychis gaubae* | LC647182 | 122,688 bp |
| *Onobrychis viciifolia* | MW007721 | 121,932 bp |
| *Oxytropis arctobia* | MT409175 | 125,271 bp |
| *Oxytropis bicolor* | MN255323 | 122,461 bp |
| *Oxytropis splendens* | MT409174 | 122,318 bp |
| *Parochetus communis* | MN966654 | 123,055 bp |
| *Pisum sativum* | HM029370 | 122,169 bp |
| *Phyllolobium chinense* | MZ127832 | 124,131 bp |
| *Sphaerophysa salsula* | MW122834 | 123,300 bp |
| *Tibetia liangshanensis* | MF193597 | 123,372 bp |
| *Trifolium boissieri* | KJ788284 | 125,740 bp |
| *Trigonella foenum-graecum* | MK460508 | 125,645 bp |
| *Vicia sativa* | KJ850242 | 122,467 bp |
| *Wisteria floribunda* | MT120817 | 130,561 bp |
|  | **Outgroup** |  |
| *Lotus japonicus* | AP002983 | 150,519 bp |
| *Robinia pseudoacacia* | KJ468102 | 154,835 bp |
