# Supplementary material for: Characterization and comparative analysis of the complete plastid genomes of four Astragalus species
Source: PLoS One. 2023 May 23;18(5):e0286083. doi: 10.1371/journal.pone.0286083 (PMC10204964; doi:10.1371/journal.pone.0286083)
Supplement: S2 Table — (DOCX) [file pone.0286083.s002.docx]

**S2 Table**. Genes with intron in the *Astragalus* plastid genomes, including the exon and intron length.

Genes with intron in the *A. iranicus* chloroplast genome, including the exon and intron length.

| **Gene name** | **Location** | **Exon I**  **(bp)** | **Intron I**  **(bp)** | **Exon II**  **(bp)** | **Intron II**  **(bp)** | **Exon III**  **(bp)** |
| --- | --- | --- | --- | --- | --- | --- |
| *ycf*3 | LSC | 117 | 693 | 228 | 748 | 153 |
| *atpF* | LSC | 168 | 679 | 411 | - | - |
| *rpoC1* | LSC | 432 | 720 | 1,623 | - | - |
| *petB* | LSC | 6 | 807 | 642 | - | - |
| *petD* | LSC | 8 | 703 | 475 | - | - |
| *rpl16* | LSC | 411 | 983 | 9 | - | - |
| *rpl2* | IR | 435 | 708 | 393 | - | - |
| *ndhB* | IR | 762 | 689 | 723 | - | - |
| *ndhA* | SSC | 552 | 1,238 | 540 | - | - |
| *clpP* | LSC | 219 | 572 | 363 | - | - |
| *rps*12 -5’ end | LSC | 114 | - | - | - | - |
| *rps*12 -3’ end | LSC | - | - | 258 | - | - |
| *trnK-UUU* | LSC | 37 | 2,480 | 29 | - | - |
| *trn*V-UAC | LSC | 39 | 601 | 37 |  |  |
| *trnL-UAA* | LSC | 37 | 551 | 50 | - | - |
| *trn*G-UCC | LSC | 23 | 700 | 48 |  |  |
| *trnA-UGC* | IR | 38 | 829 | 38 | - | - |
| *trnI-GAU* | IR | 42 | 780 | 35 | - | - |

Genes with intron in the *A. macropelmatus* chloroplast genome, including the exon and intron length.

| **Gene name** | **Location** | **Exon I**  **(bp)** | **Intron I**  **(bp)** | **Exon II**  **(bp)** | **Intron II**  **(bp)** | **Exon III**  **(bp)** |
| --- | --- | --- | --- | --- | --- | --- |
| *ycf*3 | LSC | 117 | 704 | 226 | 726 | 153 |
| *atpF* | LSC | 167 | 700 | 412 | - | - |
| *rpoC1* | LSC | 430 | 654 | 1,625 | - | - |
| *petB* | LSC | 6 | 823 | 642 | - | - |
| *petD* | LSC | 8 | 708 | 475 | - | - |
| *rpl16* | LSC | 399 | 1,001 | 9 | - | - |
| *rpl2* | IR | 435 | 705 | 387 | - | - |
| *ndhB* | IR | 723 | 685 | 762 | - | - |
| *ndhA* | SSC | 553 | 1,245 | 539 | - | - |
| *clpP* | LSC | 225 | 650 | 366 | - | - |
| *rps*12 -5’ end | LSC | 114 | - | - | - | - |
| *rps*12 -3’ end | LSC | - | - | 258 | - | - |
| *trnK-UUU* | LSC | 37 | 2,501 | 29 | - | - |
| *trn*V-UAC | LSC | 39 | 578 | 37 |  |  |
| *trnL-UAA* | LSC | 37 | 541 | 50 | - | - |
| *trn*G-UCC | LSC | 23 | 696 | 48 |  |  |
| *trnA-UGC* | IR | 38 | 799 | 35 | - | - |
| *trnI-GAU* | IR | 42 | 793 | 35 | - | - |

Genes with intron in the *A. mesoleios* chloroplast genome, including the exon and intron length.

| **Gene name** | **Location** | **Exon I**  **(bp)** | **Intron I**  **(bp)** | **Exon II**  **(bp)** | **Intron II**  **(bp)** | **Exon III**  **(bp)** |
| --- | --- | --- | --- | --- | --- | --- |
| *ycf*3 | LSC | 117 | 701 | 228 | 751 | 153 |
| *atpF* | LSC | 168 | 678 | 411 | - | - |
| *rpoC1* | LSC | 432 | 717 | 1,623 | - | - |
| *petB* | LSC | 6 | 802 | 642 | - | - |
| *petD* | LSC | 8 | 712 | 475 | - | - |
| *rpl16* | LSC | 411 | 982 | 9 | - | - |
| *rpl2* | IR | 435 | 707 | 393 | - | - |
| *ndhB* | IR | 762 | 689 | 723 | - | - |
| *ndhA* | SSC | 552 | 1,231 | 540 | - | - |
| *clpP* | LSC | 225 | 608 | 363 | - | - |
| *rps*12 -5’ end | LSC | 114 | - | - | - | - |
| *rps*12 -3’ end | LSC | - | - | 258 | - | - |
| *trnK-UUU* | LSC | 37 | 2,487 | 29 | - | - |
| *trn*V-UAC | LSC | 73 | 572 | 37 |  |  |
| *trnL-UAA* | LSC | 37 | 543 | 50 | - | - |
| *trn*G-UCC | LSC | 23 | 702 | 48 |  |  |
| *trnA-UGC* | IR | 38 | 796 | 35 | - | - |
| *trnI-GAU* | IR | 42 | 783 | 35 | - | - |

Genes with intron in the *A. odoratus* chloroplast genome, including the exon and intron length.

| **Gene name** | **Location** | **Exon I**  **(bp)** | **Intron I**  **(bp)** | **Exon II**  **(bp)** | **Intron II**  **(bp)** | **Exon III**  **(bp)** |
| --- | --- | --- | --- | --- | --- | --- |
| *ycf*3 | LSC | 117 | 704 | 228 | 751 | 153 |
| *atpF* | LSC | 168 | 679 | 411 | - | - |
| *rpoC1* | LSC | 432 | 721 | 1,623 | - | - |
| *pet*B | LSC | 6 | 814 | 642 | - | - |
| *pet*D | LSC | 8 | 705 | 475 | - | - |
| *rpl*16 | LSC | 411 | 971 | 9 | - | - |
| *rpl*2 | IR | 435 | 705 | 393 | - | - |
| *ndhB* | IR | 762 | 689 | 723 | - | - |
| *ndhA* | SSC | 552 | 1,227 | 540 | - | - |
| *clpP* | LSC | 225 | 618 | 363 | - | - |
| *rps*12 -5’ end | LSC | 114 | - | - | - | - |
| *rps*12 -3’ end | LSC | - | - | 258 | - | - |
| *trnK-UUU* | LSC | 37 | 2,464 | 29 | - | - |
| *trn*V-UAC | LSC | 39 | 599 | 37 |  |  |
| *trnL-UAA* | LSC | 37 | 539 | 50 | - | - |
| *trn*G-UCC | LSC | 24 | 702 | 48 |  |  |
| *trnA-UGC* | IR | 38 | 796 | 35 | - | - |
| *trnI-GAU* | IR | 42 | 783 | 35 | - | - |
