# Supplementary material for: Characterization and comparative analysis of the complete plastid genomes of four Astragalus species
Source: PLoS One. 2023 May 23;18(5):e0286083. doi: 10.1371/journal.pone.0286083 (PMC10204964; doi:10.1371/journal.pone.0286083)
Supplement: S3 Table — (DOCX) [file pone.0286083.s003.docx]

**S3 Table**. Codon usage in the *Astragalus* plastid genomes.

Codon usage for *A*. *iranicus* chloroplast genome.

| Amino Acid | Codon | Number | Fraction | Amino Acid | Codon | Number | Fraction |
| --- | --- | --- | --- | --- | --- | --- | --- |
| Ala | GCG  GCA  GCT  GCC | 108  192  228  109 | 0.17  0.30 0.36  0.17 | Asn | AAT  AAC | 844  426 | 0.66  0.34 |
| Cys | TGT  TGC | 336  315 | 0.52  0.48 | Pro | CCG  CCA  CCT  CCC | 150  282  225  139 | 0.19  0.35  0.28  0.17 |
| Asp | GAT  GAC | 532  173 | 0.75  0.25 | Gln | CAG  CAA | 221  498 | 0.31  0.69 |
| Glu | GAG  GAA | 256  691 | 0.27 0.73 | Arg | AGG  AGA  CGG  CGA  CGT  CGC | 339  631  160  267  188  103 | 0.20  0.37  0.09  0.16  0.11  0.06 |
| Phe | TTT  TTC | 1158  576 | 0.67 0.33 | Ser | AGT  AGC  TCG  TCA  TCT  TCC | 326  251  262  520  505  335 | 0.15  0.11  0.12  0.24  0.23  0.15 |
| Gly | GGG  GGA  GGT  GGC | 287  399  300  164 | 0.25  0.35  0.26  0.14 | Thr | ACG  ACA  ACT  ACC | 163  356  317  227 | 0.15  0.33  0.30  0.21 |
| His | CAT  CAC | 350  161 | 0.68  0.32 | Val | GTG  GTA  GTT  GTC | 157  346  403  173 | 0.15  0.32  0.37  0.16 |
| Ile | ATA  ATT  ATC | 544  922  453 | 0.28  0.48  0.24 | Trp | TGG | 542 | 1.00 |
| Lys | AAG  AAA | 464  1097 | 0.30  0.70 | Tyr | TAT  TAC | 839  408 | 0.67  0.33 |
| Leu | TTG  TTA  CTG  CTA  CTT  CTC | 558  582  183  363  494  197 | 0.23  0.24  0.08  0.15  0.21  0.08 | End | TGA  TAG  TAA | 485  381  564 | 0.34  0.27  0.39 |
| Met | ATG | 450 | 1.00 |  |  |  |  |

Codon usage for *A*. *macropelmatus* chloroplast genome.

| Amino Acid | Codon | Number | Fraction | Amino Acid | Codon | Number | Fraction |
| --- | --- | --- | --- | --- | --- | --- | --- |
| Ala | GCG  GCA  GCT  GCC | 124  254  351  135 | 0.14  0.29 0.41  0.16 | Asn | AAT  AAC | 833  366 | 0.69  0.31 |
| Cys | TGT  TGC | 238  116 | 0.67  0.33 | Pro | CCG  CCA  CCT  CCC | 178  286  290  174 | 0.19  0.31  0.31  0.19 |
| Asp | GAT  GAC | 593  205 | 0.74  0.26 | Gln | CAG  CAA | 245  580 | 0.30  0.70 |
| Glu | GAG  GAA | 333  856 | 0.28 0.72 | Arg | AGG  AGA  CGG  CGA  CGT  CGC | 213  406  151  238  221  88 | 0.16  0.31  0.11  0.18  0.17  0.07 |
| Phe | TTT  TTC | 983  567 | 0.63 0.37 | Ser | AGT  AGC  TCG  TCA  TCT  TCC | 281  110  194  345  467  264 | 0.17  0.07  0.12  0.21  0.28  0.16 |
| Gly | GGG  GGA  GGT  GGC | 243  458  409  143 | 0.19  0.37  0.33  0.11 | Thr | ACG  ACA  ACT  ACC | 138  342  358  196 | 0.13  0.33  0.35  0.19 |
| His | CAT  CAC | 326  177 | 0.65  0.35 | Val | GTG  GTA  GTT  GTC | 243  468  474  193 | 0.18  0.34  0.34  0.14 |
| Ile | ATA  ATT  ATC | 807  965  517 | 0.35  0.42  0.23 | Trp | TGG | 400 | 1.00 |
| Lys | AAG  AAA | 531  1105 | 0.32  0.68 | Tyr | TAT  TAC | 719  280 | 0.72  0.28 |
| Leu | TTG  TTA  CTG  CTA  CTT  CTC | 689  776  261  450  568  275 | 0.23  0.26  0.09  0.15  0.19  0.09 | End | TGA  TAG  TAA | 285  364  366 | 0.28  0.36  0.36 |
| Met | ATG | 660 | 1.00 |  |  |  |  |

Codon usage for *A*. *mesoleios* chloroplast genome.

| Amino Acid | Codon | Number | Fraction | Amino Acid | Codon | Number | Fraction |
| --- | --- | --- | --- | --- | --- | --- | --- |
| Ala | GCG  GCA  GCT  GCC | 108  297  474  146 | 0.1  0.29 0.46  0.14 | Asn | AAT  AAC | 970  353 | 0.73  0.27 |
| Cys | TGT  TGC | 257  149 | 0.63  0.37 | Pro | CCG  CCA  CCT  CCC | 138  267  309  158 | 0.16  0.31  0.35  0.18 |
| Asp | GAT  GAC | 682  185 | 0.79  0.21 | Gln | CAG  CAA | 199  612 | 0.25  0.75 |
| Glu | GAG  GAA | 272  918 | 0.23 0.77 | Arg | AGG  AGA  CGG  CGA  CGT  CGC | 220  498  119  292  260  101 | 0.15  0.33  0.08  0.20  0.17  0.07 |
| Phe | TTT  TTC | 1145  503 | 0.69 0.31 | Ser | AGT  AGC  TCG  TCA  TCT  TCC | 367  184  208  417  525  277 | 0.19  0.09  0.11  0.21  0.27  0.14 |
| Gly | GGG  GGA  GGT  GGC | 246  560  479  148 | 0.17  0.39  0.33  0.10 | Thr | ACG  ACA  ACT  ACC | 142  348  434  248 | 0.12  0.30  0.37  0.21 |
| His | CAT  CAC | 413  148 | 0.74  0.26 | Val | GTG  GTA  GTT  GTC | 170  443  450  162 | 0.14  0.36  0.37  0.13 |
| Ile | ATA  ATT  ATC | 591  1037  440 | 0.29  0.50  0.21 | Trp | TGG | 442 | 1.00 |
| Lys | AAG  AAA | 375  1113 | 0.25  0.75 | Tyr | TAT  TAC | 812  276 | 0.75  0.25 |
| Leu | TTG  TTA  CTG  CTA  CTT  CTC | 517  739  146  330  490  180 | 0.22  0.31  0.06  0.14  0.20  0.07 | End | TGA  TAG  TAA | 272  182  302 | 0.36  0.24  0.40 |
| Met | ATG | 473 | 1.00 |  |  |  |  |

Codon usage for *A*. *odoratus* chloroplast genome.

| Amino Acid | Codon | Number | Fraction | Amino Acid | Codon | Number | Fraction |
| --- | --- | --- | --- | --- | --- | --- | --- |
| Ala | GCG  GCA  GCT  GCC | 112  256  341  134 | 0.13  0.30 0.40  0.16 | Asn | AAT  AAC | 919  418 | 0.69  0.31 |
| Cys | TGT  TGC | 308  225 | 0.58  0.42 | Pro | CCG  CCA  CCT  CCC | 166  266  266  134 | 0.20  0.32  0.32  0.16 |
| Asp | GAT  GAC | 517  176 | 0.75  0.25 | Gln | CAG  CAA | 239  497 | 0.32  0.68 |
| Glu | GAG  GAA | 261  705 | 0.27 0.73 | Arg | AGG  AGA  CGG  CGA  CGT  CGC | 285  555  156  269  200  87 | 0.18  0.36  0.10  0.17  0.13  0.06 |
| Phe | TTT  TTC | 1173  583 | 0.69 0.33 | Ser | AGT  AGC  TCG  TCA  TCT  TCC | 348  203  235  441  500  310 | 0.17  0.10  0.12  0.22  0.25  0.15 |
| Gly | GGG  GGA  GGT  GGC | 272  496  373  157 | 0.21  0.38  0.29  0.12 | Thr | ACG  ACA  ACT  ACC | 137  324  336  239 | 0.13  0.31  0.32  0.23 |
| His | CAT  CAC | 406  176 | 0.70  0.30 | Val | GTG  GTA  GTT  GTC | 190  400  397  177 | 0.16  0.34  0.34  0.15 |
| Ile | ATA  ATT  ATC | 611  918  515 | 0.30  0.45  0.25 | Trp | TGG | 491 | 1.00 |
| Lys | AAG  AAA | 459  1135 | 0.29  0.71 | Tyr | TAT  TAC | 821  329 | 0.71  0.29 |
| Leu | TTG  TTA  CTG  CTA  CTT  CTC | 541  648  192  377  443  202 | 0.23  0.27  0.08  0.16  0.18  0.08 | End | TGA  TAG  TAA | 432  294  478 | 0.36  0.24  0.40 |
| Met | ATG | 485 | 1.00 |  |  |  |  |
