# Supplementary material for: Characterization and comparative analysis of the complete plastid genomes of four Astragalus species
Source: PLoS One. 2023 May 23;18(5):e0286083. doi: 10.1371/journal.pone.0286083 (PMC10204964; doi:10.1371/journal.pone.0286083)
Supplement: S5 Table — (DOCX) [file pone.0286083.s005.docx]

**S5 Table**. Forward, Reverse and Palindromic repeat sequences in the *Astragalus* plastid genomes.

Forward, Reverse and Palindromic repeat sequences in the *A. iranicus* chloroplast genome.

| No. | Type | Location | Size  (in bp) | Location | Region |
| --- | --- | --- | --- | --- | --- |
| 1 | Forward | 52987 | 434 | IGS (*trn*Q-UUG – *acc*D) | LSC |
| 2 | Forward | 53442 | 239 | IGS (*trn*Q-UUG – *acc*D) | LSC |
| 3 | Forward | 102501 | 92 | IGS (*trn*N-GUU – *ycf*1) | IRB |
| 4 | Forward | 19354 | 81 | *psa*A | LSC |
| 5 | Forward | 102432 | 65 | IGS (*trn*N-GUU – *ycf*1) | IRB |
| 6 | Forward | 102601 | 53 | IGS (*trn*N-GUU – *ycf*1) | IRB |
| 7 | Palindrome | 14080 | 49 | IGS (*trn*L-UAA – *trn*T-UGU) | LSC |
| 8 | Palindrome | 77381 | 49 | *rpl*16 | LSC |
| 9 | Forward | 102447 | 48 | IGS (*trn*R-ACG – *trn*N-GUU) | IRB |
| 10 | Forward | 102648 | 48 | IGS (*trn*R-ACG – *trn*N-GUU) | IRB |
| 11 | Forward | 102604 | 48 | IGS (*trn*R-ACG – *trn*N-GUU) | IRB |
| 12 | Forward | 56041 | 51 | IGS (*acc*D - *psa*I) | LSC |
| 13 | Forward | 51983 | 54 | IGS (*psb*K – *trn*Q-UUG) | LSC |
| 14 | Forward | 102447 | 44 | IGS (*trn*R-ACG – *trn*N-GUU) | IRB |
| 15 | Forward | 102702 | 44 | IGS (*trn*N-GUU – *ycf*1) | IRB |
| 16 | Forward | 102604 | 44 | IGS (*trn*R-ACG – *trn*N-GUU) | IRB |
| 17 | Forward | 102648 | 44 | IGS (*trn*R-ACG – *trn*N-GUU) | IRB |
| 18 | Palindrome | 67502 | 52 | IGS (*clp*P – *psb*B) | LSC |
| 19 | Forward | 77390 | 41 | *rpl*16 (intron) | LSC |
| 20 | Palindrome | 92504 | 40 | IGS (*rps*12 – *trn*V-GAC) | IRB |
| 21 | Forward | 105298 | 40 | *ycf*1 | IRB |
| 22 | Forward | 81785 | 48 | IGS (*rpl*23 – *trn*I-CAU) | IRB |
| 23 | Palindrome | 14084 | 37 | IGS (*trn*L-UAA – *trn*T-UGU) | LSC |
| 24 | Forward | 14171 | 37 | IGS (*trn*L-UAA – *trn*T-UGU) | LSC |
| 25 | Palindrome | 49555 | 45 | IGS (*trn*R-UCU – *trn*G-UCC) | LSC |
| 26 | Forward | 102447 | 45 | IGS (*trn*R-ACG – *trn*N-GUU) | IRB |
| 27 | Forward | 102548 | 45 | IGS (*trn*N-GUU – *ycf*1) | IRB |
| 28 | Forward | 102604 | 45 | IGS (*trn*N-GUU – *ycf*1) | IRB |
| 29 | Palindrome | 119052 | 44 | *ndh*F | SSC |
| 30 | Forward | 102702 | 44 | IGS (*trn*N-GUU – *ycf*1) | IRB |
| 31 | Forward | 81940 | 34 | IGS (*rpl*23 – *trn*I-CAU) | IRB |
| 32 | Forward | 21661 | 40 | *psa*B | LSC |
| 33 | Palindrome | 16576 | 39 | *ycf*3 (intron) | LSC |
| 34 | Palindrome | 92506 | 39 | IGS (*rps*12 – *trn*V-GAC) | IRB |
| 35 | Forward | 111571 | 38 | *ndh*A (intron) | SSC |
| 36 | Forward | 81795 | 38 | IGS (*rpl*23 – *trn*I-CAU) | IRB |
| 37 | Palindrome | 294 | 40 | IGS (*trn*H-GUG – *psb*A) | LSC |
| 38 | Palindrome | 52721 | 36 | IGS (*trn*Q-UUG – *acc*D) | LSC |
| 39 | Reverse | 107987 | 36 | *ycf*1 | IRB |
| 40 | Palindrome | 15501 | 34 | IGS (*rps*4 – *trn*S-GGA) | SSC |
| 41 | Forward | 14025 | 30 | IGS (*trn*L-UAA – *trn*T-UGU) | LSC |
| 42 | Forward | 102394 | 30 | IGS (*trn*N-GUU – *ycf*1) | IRB |
| 43 | Forward | 19413 | 35 | *psa*A | LSC |
| 44 | Palindrome | 46246 | 35 | *rpo*C2 | LSC |
| 45 | Palindrome | 108042 | 35 | IGS (*ycf*1 – *rps*15) | IRB |
| 46 | Forward | 24871 | 32 | *trn*S-UGA | LSC |
| 47 | Forward | 86153 | 34 | *ycf2* | IRB |
| 48 | Reverse | 76399 | 31 | IGS (*rps*8 – *rpl*14) | LSC |
| 49 | Palindrome | 90029 | 33 | *ndh*B (intron) | IRB |
| 50 | Complement | 44000 | 30 | IGS (*rps*2 – *atp*I) | LSC |

P: means palindromic, F: means forward, R: means reverse and IGS: means intergenic spacer.

Forward, Reverse and Palindromic repeat sequences in the *A. macropelmatus* chloroplast genome.

| No. | Type | Location | Size  (in bp) | Location | Region |
| --- | --- | --- | --- | --- | --- |
| 1 | Forward | 82965 | 171 | IGS (*trn*I-CAU – *ycf*2) | IRB |
| 2 | Forward | 83087 | 108 | IGS (*trn*I-CAU – *ycf*2) | IRB |
| 3 | Forward | 19611 | 81 | *psa*A | LSC |
| 4 | Forward | 108372 | 69 | *ycf*1 | IRB |
| 5 | Forward | 109220 | 76 | *ycf*1 | IRB |
| 6 | Forward | 106011 | 63 | *ycf*1 | IRB |
| 7 | Forward | 109565 | 67 | *ycf*1 | IRB |
| 8 | Forward | 109297 | 63 | *ycf*1 | IRB |
| 9 | Palindrome | 62705 | 66 | IGS (*trn*W-CCA-*trn*P-UGG) | LSC |
| 10 | Forward | 108396 | 57 | *ycf*1 | IRB |
| 11 | Forward | 83135 | 62 | IGS (*trn*I-CAU – *ycf*2) | IRB |
| 12 | Forward | 83013 | 60 | IGS (*trn*I-CAU – *ycf*2) | IRB |
| 13 | Forward | 56491 | 63 | IGS (*acc*D – *psa*I) | LSC |
| 14 | Palindrome | 66381 | 52 | IGS (*rps*12 – *clp*P) | LSC |
| 15 | Palindrome | 120198 | 52 | IGS (*trn*L-UAG – *rpl*32) | SSC |
| 16 | Forward | 85403 | 60 | *ycf*2 | IRB |
| 17 | Forward | 56506 | 48 | IGS (*acc*D – *psa*I) | LSC |
| 18 | Forward | 82450 | 48 | IGS (*rpl*23 – *trn*I-CAU) | IRB |
| 19 | Palindrome | 120281 | 51 | IGS (*trn*L-UAG – *rpl*32) | SSC |
| 20 | Forward | 108408 | 45 | *ycf*1 | IRB |
| 21 | Palindrome | 66463 | 44 | IGS (*rps*12 – *clp*P) | LSC |
| 22 | Forward | 98481 | 44 | IGS (*rrn*16 – *trn*V-GAC) | IRB |
| 23 | Forward | 109249 | 47 | *ycf*1 | IRB |
| 24 | Palindrome | 190 | 53 | IGS (*trn*H-GUG *- psb*A) | LSC |
| 25 | Forward | 79435 | 52 | IGS (*rpl*16 – *rps*3) | LSC |
| 26 | Palindrome | 78012 | 49 | *rpl*16 (intron) | LSC |
| 27 | Forward | 104073 | 42 | IGS (*ndh*B – *ycf*1) | IRB |
| 28 | Forward | 106053 | 42 | *ycf*1 | IRB |
| 29 | Palindrome | 100466 | 37 | *rps*12 | IRB |
| 30 | Forward | 109409 | 50 | *ycf*1 | IRB |
| 31 | Forward | 83092 | 44 | IGS (*trn*I-CAU – *ycf*2) | IRB |
| 32 | Forward | 83148 | 47 | IGS (*trn*I-CAU – *ycf*2) | IRB |
| 33 | Forward | 109399 | 47 | *ycf*1 | IRB |
| 34 | Palindrome | 104171 | 39 | IGS (*ndh*B – *ycf*1) | IRB |
| 35 | Forward | 109296 | 39 | *ycf*1 | IRB |
| 36 | Forward | 83031 | 42 | IGS (*trn*I-CAU – *ycf*2) | IRB |
| 37 | Forward | 109594 | 38 | *ycf*1 | IRB |
| 38 | Forward | 98470 | 47 | IGS (*rrn*16 – *trn*V-GAC) | IRB |
| 39 | Forward | 85500 | 41 | *ycf*2 | IRB |
| 40 | Forward | 23351 | 37 | IGS (*psa*B – *rps*14) | SSC |
| 41 | Palindrome | 50780 | 45 | *trn*R-UCU | LSC |
| 42 | Forward | 79499 | 39 | *rps*3 | LSC |
| 43 | Forward | 106782 | 39 | *ycf*1 | IRB |
| 44 | Forward | 83101 | 35 | IGS (*trn*I-CAU – *ycf*2) | IRB |
| 45 | Palindrome | 56250 | 40 | IGS (*acc*D – *psa*I) | LSC |
| 46 | Forward | 113505 | 40 | ndhA (intron) | SSC |
| 47 | Forward | 82979 | 33 | IGS (*trn*I-CAU – *ycf*2) | IRB |
| 48 | Forward | 83040 | 33 | IGS (*trn*I-CAU – *ycf*2) | IRB |
| 49 | Forward | 108372 | 33 | *ycf*1 | IRB |
| 50 | Palindrome | 16852 | 39 | *ycf3* (intron) | LSC |

P: means palindromic, F: means forward, R: means reverse and IGS: means intergenic spacer.

Forward, Reverse and Palindromic repeat sequences in the *A. mesoleios* chloroplast genome.

| No. | Type | Location | Size  (in bp) | Location | Region |
| --- | --- | --- | --- | --- | --- |
| 1 | Forward | 102914 | 114 | IGS (*trn*N-GUU – *ycf*1) | IRB |
| 2 | Palindrome | 67950 | 80 | IGS (*clp*P – *psb*B) | LSC |
| 3 | Palindrome | 120539 | 72 | *ndh*F | SSC |
| 4 | Forward | 19890 | 81 | *psa*A | LSC |
| 5 | Forward | 56304 | 66 | IGS (*acc*D – *psa*I) | LSC |
| 6 | Forward | 56717 | 48 | IGS (*psa*I – *ycf*4) | LSC |
| 7 | Palindrome | 77964 | 49 | *rpl*16 (intron) | LSC |
| 8 | Forward | 107274 | 42 | *ycf*1 | IRB |
| 9 | Forward | 93133 | 41 | IGS (*rps*12 – *trn*V-GAC) | IRB |
| 10 | Palindrome | 112105 | 40 | *ndh*A (intron) | SSC |
| 11 | Palindrome | 50470 | 45 | *trn*R-UCU | LSC |
| 12 | Forward | 102897 | 34 | IGS (*trn*N-GUU – *ycf*1) | IRB |
| 13 | Forward | 103108 | 33 | IGS (*trn*N-GUU – *ycf*1) | IRB |
| 14 | Palindrome | 17123 | 39 | *ycf*3 (intron) | LSC |
| 15 | Palindrome | 93135 | 39 | IGS (*rps*12 – *trn*V-GAC) | IRB |
| 16 | Forward | 82358 | 35 | IGS (*rpl*23 – *trn*I-CAU) | IRB |
| 17 | Forward | 19973 | 40 | *psa*A | LSC |
| 18 | Forward | 107298 | 30 | *ycf1* | IRB |
| 19 | Forward | 112105 | 38 | *ndh*A (intron) | SSC |
| 20 | Reverse | 82431 | 35 | IGS (*rpl*23 – *trn*I-CAU) | IRB |
| 21 | Palindrome | 16036 | 34 | *trn*S-GGA | LSC |
| 22 | Forward | 103026 | 36 | IGS (*trn*N-GUU – *ycf*1) | IRB |
| 23 | Forward | 103088 | 36 | IGS (*trn*N-GUU – *ycf*1) | IRB |
| 24 | Forward | 82595 | 30 | IGS (*rpl*23 – *trn*I-CAU) | IRB |
| 25 | Palindrome | 314 | 35 | IGS (*trn*H-GUG - *psb*B) | LSC |
| 26 | Forward | 22173 | 35 | *psa*B | LSC |
| 27 | Palindrome | 47149 | 35 | IGS (*atp*H - *atp*F) | LSC |
| 28 | Palindrome | 108482 | 35 | IGS (*ycf*1 – *rps*15) | IRB |
| 29 | Forward | 25414 | 32 | *trn*S-UGA | LSC |
| 30 | Palindrome | 62904 | 32 | IGS (*trn*W-CCA – *trn*P-UGG) | LSC |
| 31 | Palindrome | 82454 | 32 | IGS (*rpl*23 – *trn*I-CAU) | IRB |
| 32 | Forward | 102979 | 32 | IGS (*trn*N-GUU – *ycf*1) | IRB |
| 33 | Forward | 102917 | 32 | IGS (*trn*N-GUU – *ycf*1) | IRB |
| 34 | Palindrome | 71588 | 33 | *pet*B (intron) | LSC |
| 35 | Palindrome | 116966 | 30 | IGS (*ndh*D – *ccs*A) | SSC |
| 36 | Forward | 82625 | 32 | IGS (*rpl*23 – *trn*I-CAU) | IRB |
| 37 | Palindrome | 91111 | 31 | *ndh*B (intron) | IRB |
| 38 | Complement | 106689 | 31 | *ycf*1 | IRB |
| 39 | Complement | 41915 | 30 | *rpo*C2 | LSC |
| 40 | Forward | 53025 | 30 | IGS (*psb*K – *trn*Q-UUG) | LSC |
| 41 | Forward | 56382 | 30 | *psa*I | LSC |
| 42 | Forward | 63223 | 30 | IGS (*trn*P-UGG – *psa*J) | LSC |

P: means palindromic, F: means forward, R: means reverse and IGS: means intergenic spacer.

Forward, Reverse and Palindromic repeat sequences in the *A. odoratus* chloroplast genome.

| No. | Type | Location | Size  (in bp) | Location | Region |
| --- | --- | --- | --- | --- | --- |
| 1 | Forward | 20073 | 81 | *psa*A | LSC |
| 2 | Palindrome | 69037 | 67 | IGS (*clp*P – *psb*B) | LSC |
| 3 | Forward | 107072 | 61 | *ycf*1 | IRB |
| 4 | Forward | 83587 | 60 | IGS (*rpl*23 – *trn*I-CAU) | LSC |
| 5 | Forward | 57349 | 66 | IGS (*acc*D – *psa*I) | LSC |
| 6 | Forward | 104361 | 55 | IGS (*trn*N-GUU – *ycf*1) | IRB |
| 7 | Forward | 107101 | 53 | *ycf*1 | IRB |
| 8 | Palindrome | 309 | 63 | IGS (*trn*H-GUG – *psb*A) | LSC |
| 9 | Palindrome | 121937 | 52 | *ndh*F | SSC |
| 10 | Forward | 57367 | 48 | IGS (*acc*D – *psa*I) | LSC |
| 11 | Palindrome | 79069 | 49 | *rpl*16 (intron) | LSC |
| 12 | Forward | 94260 | 39 | IGS (*rps*12 – *trn*V-GAC) | IRB |
| 13 | Forward | 84525 | 48 | *ycf*2 | IRB |
| 14 | Palindrome | 120157 | 38 | IGS (*trn*L-UAG – *rpl*32) | SSC |
| 15 | Palindrome | 113530 | 40 | *ndh*A (intron) | SSC |
| 16 | Forward | 107072 | 40 | *ycf*1 | IRB |
| 17 | Palindrome | 51268 | 45 | *trn*R-UCU | LSC |
| 18 | Palindrome | 17281 | 42 | *ycf*3 (intron) | LSC |
| 19 | Palindrome | 113530 | 38 | *ndh*A (intron) | SSC |
| 20 | Palindrome | 94260 | 39 | IGS (*rps*12 – *trn*V-GAC) | IRB |
| 21 | Forward | 104346 | 32 | IGS (*trn*N-GUU – *ycf*1) | IRB |
| 22 | Forward | 107122 | 32 | *ycf*1 | IRB |
| 23 | Forward | 20156 | 40 | *psa*A | LSC |
| 24 | Forward | 104290 | 37 | IGS (*trn*N-GUU – *ycf*1) | IRB |
| 25 | Forward | 14516 | 30 | IGS (*trn*L-UAA – *trn*T-UGU) | LSC |
| 26 | Palindrome | 63900 | 30 | IGS (*trn*W-CCA – *trn*P-UGG) | LSC |
| 27 | Palindrome | 109891 | 30 | IGS (*ycf*1 – *rps*15) | IRB |
| 28 | Forward | 104449 | 33 | IGS (*trn*N-GUU – *ycf*1) | IRB |
| 29 | Forward | 17282 | 38 | *ycf*3 (intron) | LSC |
| 30 | Forward | 83781 | 32 | IGS (*rpl*23 – *trn*I-CAU) | IRB |
| 31 | Palindrome | 64518 | 37 | IGS (*pet*L – *pet*G) | LSC |
| 32 | Palindrome | 16191 | 34 | *trn*S-GGA | LSC |
| 33 | Forward | 14500 | 31 | IGS (*trn*L-UAA – *trn*T-UGU) | LSC |
| 34 | Palindrome | 18526 | 31 | IGS (*ycf*3 – *psa*A) | LSC |
| 35 | Forward | 108660 | 30 | *ycf*1 | IRB |
| 36 | Palindrome | 4626 | 35 | IGS (*trn*K-UUU – *rbc*L) | LSC |
| 37 | Forward | 20132 | 35 | *psa*A | LSC |
| 38 | Palindrome | 47966 | 35 | IGS (*atp*H – *atp*F) | LSC |
| 39 | Palindrome | 109862 | 35 | IGS (*ycf*1 – *rps*15) | IRB |
| 40 | Forward | 25632 | 32 | *trn*S-UGA | LSC |
| 41 | Reverse | 81362 | 31 | IGS (*rps*19 – *rpl*2) | IRB |
| 42 | Palindrome | 79070 | 33 | *rpl*16 (intron) | LSC |
| 43 | Palindrome | 72674 | 33 | *pet*B (intron) | LSC |
| 44 | Forward | 14504 | 30 | IGS (*trn*L-UAA – *trn*T-UGU) | LSC |
| 45 | Palindrome | 48032 | 30 | IGS (*atp*H – *atp*F) | LSC |
| 46 | Reverse | 54493 | 30 | IGS (*trn*Q-UUG – *acc*D) | LSC |
| 47 | Reverse | 78058 | 30 | IGS (*rps*8 – *rpl*14) | LSC |
| 48 | Palindrome | 118575 | 30 | IGS (*ndh*D – *ccs*A) | SSC |
| 49 | Palindrome | 114807 | 31 | IGS (*ndh*I – *ndh*G) | SSC |
| 50 | Reverse | 64189 | 31 | IGS (*trn*P-UGG – *psa*J) | LSC |

P: means palindromic, F: means forward, R: means reverse and IGS: means intergenic spacer.
