# Supplementary material for: Characterization and comparative analysis of the complete plastid genomes of four Astragalus species
Source: PLoS One. 2023 May 23;18(5):e0286083. doi: 10.1371/journal.pone.0286083 (PMC10204964; doi:10.1371/journal.pone.0286083)
Supplement: S6 Table — (DOCX) [file pone.0286083.s006.docx]

**S6 Table**. SSRs showing in the plastomes of Astragalus species.

Distribution of simple sequence repeat (SSR) in the A. iranicus chloroplast genome.

| No. SSR | SSR type | SSR | Size | Start | End | Location |
| --- | --- | --- | --- | --- | --- | --- |
| 1 | p1 | (T)10 | 10 | 211 | 220 | LSC |
| 2 | c | (T)10acctcatttaaatttaatcattcattcaaaaaaaagatactaaaggttttctatagaatctatct(ATAG)3 | 87 | 1624 | 1710 | LSC |
| 3 | p1 | (T)11 | 11 | 2281 | 2291 | LSC |
| 4 | p1 | (A)13 | 13 | 4336 | 4348 | LSC |
| 5 | c | (ATTA)3atataattaat(ATA)5 | 38 | 4454 | 4491 | LSC |
| 6 | p1 | (A)13 | 13 | 7010 | 7022 | LSC |
| 7 | p1 | (A)11 | 11 | 7367 | 7377 | LSC |
| 8 | c | (AT)5tactta(AT)6 | 28 | 9528 | 9555 | LSC |
| 9 | p4 | (TTTA)3 | 12 | 9995 | 10006 | LSC |
| 10 | p4 | (AATA)3 | 12 | 12784 | 12795 | LSC |
| 11 | p1 | (A)12 | 12 | 12986 | 12997 | LSC |
| 12 | p2 | (AT)5 | 10 | 13350 | 13359 | LSC |
| 13 | p1 | (A)11 | 11 | 13758 | 13768 | LSC |
| 14 | c | (AT)12aaaaaaaactaaaagcaaaaatccattta(AT)5agataaatataataatagatatagataaatataataatagaagagatataataaataagaattatgtgttagatgtaa(AT)5 | 151 | 14026 | 14176 | LSC |
| 15 | p1 | (T)14 | 14 | 14469 | 14482 | LSC |
| 16 | p1 | (T)11 | 11 | 15975 | 15985 | LSC |
| 17 | c | (TA)5tttctttctttttctctatttagaatatatattaattattaatataaaataaattcttaatctatattga(AT)7 | 94 | 17870 | 17963 | LSC |
| 18 | p2 | (GA)5 | 10 | 23125 | 23134 | LSC |
| 19 | p1 | (T)10 | 10 | 24004 | 24013 | LSC |
| 20 | p4 | (TTTA)3 | 12 | 24922 | 24933 | LSC |
| 21 | c | (A)11tagggctatcttacaattataggagatatcacattctaaaaaaagaatatatattaaaga(AT)5 | 81 | 28235 | 28315 | LSC |
| 22 | p2 | (TA)5 | 10 | 28731 | 28740 | LSC |
| 23 | c | (A)12gactc(T)11 | 28 | 29489 | 29516 | LSC |
| 24 | p1 | (T)10 | 10 | 29878 | 29887 | LSC |
| 25 | p3 | (ATA)4 | 12 | 31058 | 31069 | LSC |
| 26 | p4 | (GATT)3 | 12 | 32158 | 32169 | LSC |
| 27 | p2 | (AT)5 | 10 | 35092 | 35101 | LSC |
| 28 | p1 | (T)10 | 10 | 36591 | 36600 | LSC |
| 29 | p1 | (T)10 | 10 | 36791 | 36800 | LSC |
| 30 | p2 | (AT)5 | 10 | 39637 | 39646 | LSC |
| 31 | c | (A)13tggaattaaaaatattttctggaaatatctattttcccggagagatggataagatatcccgacccagtgccatgttgataccacccagaacggt(A)12 | 119 | 41010 | 41128 | LSC |
| 32 | p1 | (A)10 | 10 | 42889 | 44289 | LSC |
| 33 | p1 | (A)10 | 10 | 45116 | 45125 | LSC |
| 34 | p1 | (A)15 | 15 | 46462 | 46476 | LSC |
| 35 | p1 | (A)10 | 10 | 47120 | 47129 | LSC |
| 36 | p3 | (AAT)4 | 12 | 49637 | 49648 | LSC |
| 37 | p1 | (T)13 | 13 | 50735 | 50747 | LSC |
| 38 | p1 | (A)10 | 10 | 51436 | 51445 | LSC |
| 39 | p1 | (T)11 | 11 | 51974 | 51984 | LSC |
| 40 | p3 | (TAT)4 | 12 | 52725 | 52736 | LSC |
| 41 | p1 | (A)10 | 10 | 53230 | 53239 | LSC |
| 42 | p1 | (A)10 | 10 | 53924 | 53933 | LSC |
| 43 | p1 | (A)11 | 11 | 56043 | 56053 | LSC |
| 44 | p1 | (A)11 | 11 | 56355 | 56365 | LSC |
| 45 | p1 | (A)10 | 10 | 59480 | 59489 | LSC |
| 46 | p1 | (T)11 | 11 | 61085 | 61095 | LSC |
| 47 | p1 | (A)10 | 10 | 61470 | 61479 | LSC |
| 48 | p1 | (T)10 | 10 | 61675 | 61684 | LSC |
| 49 | c | (T)11cataaa(T)10 | 27 | 62372 | 62398 | LSC |
| 50 | p1 | (T)10 | 10 | 62696 | 62705 | LSC |
| 51 | p4 | (ATTA)3 | 12 | 63034 | 63045 | LSC |
| 52 | c* | (ATT)3(AT<T>A)(TA)5 | 23 | 64182 | 64204 | LSC |
| 53 | c | (A)11tttgaaaaaagcgagtcgaccactagaactactacgactgttctt(A)15 | 71 | 64588 | 64658 | LSC |
| 54 | p1 | (T)12 | 12 | 64800 | 64811 | LSC |
| 55 | p2 | (TA)6 | 12 | 66067 | 66078 | LSC |
| 56 | p1 | (A)11 | 11 | 66909 | 66919 | LSC |
| 57 | p3 | (ATA)4 | 12 | 76207 | 76218 | LSC |
| 58 | c | (T)11atctttcagtcaaaaagttgaagtaaaaaaaatattctgtgttc(A)14 | 69 | 76831 | 76899 | LSC |
| 59 | p1 | (T)10 | 10 | 77745 | 77754 | LSC |
| 60 | p4 | (TTTC)3 | 12 | 78083 | 78094 | LSC |
| 61 | p1 | (T)15 | 15 | 79698 | 79712 | IRB |
| 62 | p2 | (TA)5 | 10 | 80357 | 80366 | IRB |
| 63 | p2 | (TA)6 | 12 | 81683 | 81694 | IRB |
| 64 | c* | (AAT)5ctaattaaagtaatt(A)10(AAT)5* | 53 | 81973 | 82025 | IRB |
| 65 | c* | (AT)6gtaatatgtaataatatata(AT)5a(ATAAT)3(ATA)4* | 65 | 92967 | 93031 | IRB |
| 66 | p4 | (AGGT)3 | 12 | 99590 | 99601 | IRB |
| 67 | p1 | (T)10 | 10 | 104298 | 104307 | IRB |
| 68 | p1 | (A)10 | 10 | 104418 | 104427 | IRB |
| 69 | c | (A)11gatctactgc(A)11 | 32 | 105309 | 105340 | IRB |
| 70 | p4 | (CAAA)3 | 12 | 106696 | 106707 | IRB |
| 71 | c | (AT)5ttataaatgcgcacacgcatcattgatactaattcaatgattttagatagaaaaatgaatcgaaaaaatcgtcttc(T)12 | 98 | 108077 | 108174 | SSC |
| 72 | p1 | (T)10 | 10 | 108632 | 108641 | SSC |
| 73 | p1 | (A)10 | 10 | 111124 | 111133 | SSC |
| 74 | p1 | (T)11 | 11 | 113660 | 113670 | SSC |
| 75 | p4 | (ATCA)3 | 12 | 114062 | 114073 | SSC |
| 76 | p4 | (TATT)3 | 12 | 116058 | 116069 | SSC |
| 77 | p1 | (A)10 | 10 | 116358 | 116367 | SSC |
| 78 | p2 | (AT)5 | 10 | 117500 | 117509 | SSC |
| 79 | p1 | (T)11 | 11 | 118015 | 118025 | SSC |
| 80 | p1 | (A)10 | 10 | 118170 | 118179 | SSC |

Distribution of simple sequence repeat (SSR) in the A. macropelmatus chloroplast genome.

| No. SSR | SSR type | SSR | Size | Start | End | Location |
| --- | --- | --- | --- | --- | --- | --- |
| 1 | p1 | (T)11 | 11 | 2173 | 2183 | LSC |
| 2 | p1 | (A)10 | 10 | 3989 | 3998 | LSC |
| 3 | c | (A)12taggatttattatagatacaaatcaagaatagaaacttttcatttttttggcttaa(T)14 | 82 | 4808 | 4889 | LSC |
| 4 | c | (TA)5tttattat(TA)5 | 28 | 6786 | 6813 | LSC |
| 5 | p1 | (A)12 | 12 | 7098 | 7109 | LSC |
| 6 | p1 | (A)11 | 11 | 7359 | 7369 | LSC |
| 7 | p3 | (TAA)4 | 12 | 9300 | 9311 | LSC |
| 8 | p1 | (T)10 | 10 | 12469 | 12478 | LSC |
| 9 | p2 | (AT)5 | 10 | 12998 | 13007 | LSC |
| 10 | p2 | (AT)5 | 10 | 13545 | 13554 | LSC |
| 11 | c | (AT)6 (TA)6 | 24 | 14494 | 14517 | LSC |
| 12 | p1 | (T)10 | 10 | 14752 | 14761 | LSC |
| 13 | p1 | (T)10 | 10 | 16240 | 16249 | LSC |
| 14 | p2 | (TA)6 | 12 | 18146 | 18157 | LSC |
| 15 | p2 | (GA)5 | 10 | 23492 | 23501 | LSC |
| 16 | p1 | (T)10 | 10 | 24394 | 24403 | LSC |
| 17 | p1 | (G)10 | 10 | 24737 | 24746 | LSC |
| 18 | p1 | (T)11 | 11 | 25147 | 25157 | LSC |
| 19 | c | (A)11gtc(T)14 | 28 | 29960 | 29987 | LSC |
| 20 | p3 | (ATA)4 | 12 | 31881 | 31892 | LSC |
| 21 | p1 | (A)13 | 13 | 32009 | 32021 | LSC |
| 22 | c | (AT)5gagaatacccctgccctcaaaaaaccaaacaaaaaggggacggggttctaaacttc(T)10 | 76 | 32789 | 32864 | LSC |
| 23 | p2 | (AT)5 | 10 | 36354 | 36363 | LSC |
| 24 | p1 | (A)10 | 10 | 37840 | 37849 | LSC |
| 25 | c | (A)13tggaattaaaaatattttctggaaatatccattttcccggagagatggataagatatcccgacccagtgccatcttgataccacccagaacggt(A)11 | 118 | 42210 | 42327 | LSC |
| 26 | p1 | (A)13 | 13 | 44101 | 44113 | LSC |
| 27 | p1 | (T)10 | 10 | 44405 | 44414 | LSC |
| 28 | p1 | (A)10 | 10 | 44667 | 44676 | LSC |
| 29 | p1 | (T)11 | 11 | 47228 | 47238 | LSC |
| 30 | p4 | (CTTA)3 | 12 | 47538 | 47549 | LSC |
| 31 | c | (T)12cataattgaaattcaaaaaaatat(TA)5 | 46 | 50638 | 50683 | LSC |
| 32 | p2 | (TA)5 | 10 | 51160 | 51169 | LSC |
| 33 | p1 | (T)11 | 11 | 52073 | 52083 | LSC |
| 34 | p1 | (T)11 | 11 | 52287 | 52297 | LSC |
| 35 | p1 | (A)11 | 11 | 52771 | 52781 | LSC |
| 36 | p4 | (TTTA)3 | 12 | 53328 | 53339 | LSC |
| 37 | p2 | (TA)5 | 10 | 53486 | 53495 | LSC |
| 38 | p4 | (ATAA)3 | 12 | 53623 | 53634 | LSC |
| 39 | p5 | (ATAAT)3 | 15 | 53758 | 53772 | LSC |
| 40 | p1 | (A)11 | 11 | 54327 | 54337 | LSC |
| 41 | p1 | (T)10 | 10 | 58390 | 58399 | LSC |
| 42 | p1 | (A)10 | 10 | 59972 | 59981 | LSC |
| 43 | p2 | (AT)5 | 10 | 60091 | 60100 | LSC |
| 44 | p5 | (AATAA)3 | 15 | 63426 | 63440 | LSC |
| 45 | p3 | (TAT)4 | 12 | 64443 | 64454 | LSC |
| 46 | c | (A)11tttgaaaaaagcaagtcgaccactagaactactacgacttttatt(A)15 | 71 | 64879 | 64949 | LSC |
| 47 | p1 | (T)11 | 11 | 65091 | 65101 | LSC |
| 48 | p5 | (TTCGA)3 | 15 | 69866 | 69880 | LSC |
| 49 | p1 | (A)10 | 10 | 71381 | 71390 | LSC |
| 50 | p1 | (T)10 | 10 | 76121 | 76130 | LSC |
| 51 | p1 | (A)13 | 13 | 76848 | 76860 | LSC |
| 52 | p1 | (A)10 | 10 | 77521 | 77530 | LSC |
| 53 | c | (T)13atcatatcggattggcaaaaattttgaaat(A)11 | 54 | 78198 | 78251 | LSC |
| 54 | p1 | (A)10 | 10 | 78406 | 78415 | LSC |
| 55 | p1 | (T)10 | 10 | 80386 | 80395 | IRB |
| 56 | p2 | (TA)5 | 10 | 81034 | 81043 | IRB |
| 57 | p2 | (AT)5 | 10 | 82353 | 82362 | IRB |
| 58 | c | (TA)6ataatctaattgaagttaaagtaatt(TA)5ataatctaattgaagtttagtaattaaattcaaaaataatataataataattgaagtaaagtaataataaatttct(TA)7ataataaat(TA)5 | 157 | 82582 | 82738 | IRB |
| 59 | p3 | (TGA)4 | 12 | 87080 | 87091 | IRB |
| 60 | p1 | (A)10 | 10 | 91307 | 91316 | IRB |
| 61 | p4 | (CTAC)3 | 12 | 92891 | 92902 | IRB |
| 62 | p5 | (AGCTC)3 | 15 | 96795 | 96809 | IRB |
| 63 | c | (AT)5taatatattatataatattatatat(TA)5 | 45 | 99622 | 99666 | IRB |
| 64 | p6 | (TGAAAA)6 | 36 | 105519 | 105554 | IRB |
| 65 | p1 | (T)10 | 10 | 105699 | 105708 | IRB |
| 66 | p1 | (A)10 | 10 | 105819 | 105828 | IRB |
| 67 | p1 | (A)10 | 10 | 106430 | 106439 | IRB |
| 68 | c | (A)10ttaaaaaacaatatagatatgatcttttgtcatataaatatctaaattacgcagataagaaaaattcatatatttatggatatagatcaccattc(CAAA)3 | 117 | 108082 | 108198 | IRB |
| 69 | p1 | (T)12 | 12 | 109973 | 109984 | SSC |
| 70 | p1 | (A)11 | 11 | 113048 | 113058 | SSC |
| 71 | p4 | (TATT)3 | 12 | 118126 | 118137 | SSC |
| 72 | p1 | (A)10 | 10 | 118427 | 118436 | SSC |
| 73 | p2 | (AT)6 | 12 | 119570 | 119581 | SSC |
| 74 | c | (TAT)6ttcacactacattctaaaaaatttagattatatattcagcatatattcta(AT)5tagaatatatgctgaatatatggtcaatattaaatattcttatattactaaatattctttatattct(TTA)4 | 157 | 120152 | 120308 | SSC |
| 75 | p1 | (T)11 | 11 | 120481 | 120491 | SSC |
| 76 | p2 | (AT)5 | 10 | 121019 | 121028 | SSC |
| 77 | p2 | (TA)6 | 12 | 123490 | 123501 | SSC |

Distribution of simple sequence repeat (SSR) in the A. mesoleios chloroplast genome.

| No. SSR | SSR type | SSR | Size | Start | End | Location |
| --- | --- | --- | --- | --- | --- | --- |
| 1 | p1 | (T)11 | 11 | 220 | 230 | LSC |
| 2 | p1 | (T)13 | 13 | 1631 | 1643 | LSC |
| 3 | p1 | (T)11 | 11 | 2288 | 2298 | LSC |
| 4 | p1 | (A)15 | 15 | 4355 | 4369 | LSC |
| 5 | p2 | (AT)6 | 12 | 4504 | 4515 | LSC |
| 6 | p2 | (TA)5 | 10 | 6819 | 6828 | LSC |
| 7 | p1 | (A)16 | 16 | 6997 | 7012 | LSC |
| 8 | p1 | (A)10 | 10 | 7396 | 7405 | LSC |
| 9 | p2 | (AT)7 | 14 | 9560 | 9573 | LSC |
| 10 | p4 | (TTTA)3 | 12 | 10018 | 10029 | LSC |
| 11 | p2 | (AT)5 | 10 | 10423 | 10432 | LSC |
| 12 | p2 | (AT)6 | 12 | 13112 | 13123 | LSC |
| 13 | p1 | (A)12 | 12 | 13305 | 13316 | LSC |
| 14 | p1 | (A)14 | 14 | 14067 | 14080 | LSC |
| 15 | p4 | (ATCT)3 | 12 | 14331 | 14342 | LSC |
| 16 | p2 | (AT)5 | 10 | 14752 | 14761 | LSC |
| 17 | p2 | (TA)5 | 10 | 18436 | 18445 | LSC |
| 18 | p2 | (GA)5 | 10 | 23661 | 23670 | LSC |
| 19 | p1 | (T)11 | 11 | 24518 | 24528 | LSC |
| 20 | c | (T)11agaatttttcccatttttttgattggaat(A)10taggactatctgacaattataggagatatcacattctaaaaaaagataagaatatatattatagaaataattataat(TA)5 | 137 | 28746 | 28882 | LSC |
| 21 | c | (A)12gagtc(T)11cattgattaattaatttttcaattgatttggcaataacgaacggattactcgtaatccgtgtcgatctgcgctaaagtgcagaccc(AT)5 | 124 | 30020 | 30143 | LSC |
| 22 | p1 | (A)10 | 10 | 32423 | 32432 | LSC |
| 23 | p2 | (AT)5 | 10 | 35994 | 36003 | LSC |
| 24 | p2 | (AT)5 | 10 | 40536 | 40545 | LSC |
| 25 | p1 | (A)13 | 13 | 41915 | 41927 | LSC |
| 26 | p1 | (A)10 | 10 | 43806 | 43815 | LSC |
| 27 | p1 | (T)10 | 10 | 44108 | 44117 | LSC |
| 28 | p1 | (A)10 | 10 | 44373 | 44382 | LSC |
| 29 | p3 | (TAT)4 | 12 | 44937 | 44948 | LSC |
| 30 | c | (CTTA)3tatgaatattcttactaatcgtaattattagtaagaatattcatattaagtacgaaatgaaaattttattattttttattataatttctttatt(ATA)4 | 118 | 47213 | 47330 | LSC |
| 31 | p1 | (A)11 | 11 | 48033 | 48043 | LSC |
| 32 | p2 | (TA)6 | 12 | 50362 | 50373 | LSC |
| 33 | p1 | (T)10 | 10 | 52217 | 52226 | LSC |
| 34 | p1 | (A)12 | 12 | 52481 | 52492 | LSC |
| 35 | p1 | (T)10 | 10 | 53012 | 53021 | LSC |
| 36 | p3 | (ATA)4 | 12 | 53286 | 53297 | LSC |
| 37 | p3 | (TAT)4 | 12 | 53778 | 53789 | LSC |
| 38 | p1 | (A)11 | 11 | 54241 | 54251 | LSC |
| 39 | p4 | (TATC)3 | 12 | 56199 | 56210 | LSC |
| 40 | p1 | (A)11 | 11 | 61937 | 61947 | LSC |
| 41 | p1 | (T)13 | 13 | 62848 | 62860 | LSC |
| 42 | p1 | (T)13 | 13 | 63175 | 63187 | LSC |
| 43 | p2 | (AT)5 | 10 | 63338 | 63347 | LSC |
| 44 | c | (AT)5aacaaacattaacaaacatatagaaaaaaataagaat(ATAA)3 | 59 | 64675 | 64733 | LSC |
| 45 | c | (A)11tttgaaaaaagcgagtcgaccactagaactactacgactgttctt(A)15 | 71 | 65037 | 65107 | LSC |
| 46 | p1 | (T)15 | 15 | 65249 | 65263 | LSC |
| 47 | p1 | (T)15 | 15 | 65898 | 65912 | LSC |
| 48 | p2 | (TA)6 | 12 | 66509 | 66520 | LSC |
| 49 | p1 | (T)10 | 10 | 72382 | 72391 | LSC |
| 50 | c | (AT)5ttcattccttattttttctatctataatat(TA)5 | 50 | 76914 | 76963 | LSC |
| 51 | p1 | (A)10 | 10 | 77474 | 77483 | LSC |
| 52 | c | (T)12ac(T)10at(A)11 | 37 | 78323 | 78359 | LSC |
| 53 | p4 | (TTTC)4 | 16 | 78667 | 78682 | LSC |
| 54 | p2 | (TA)5 | 10 | 80928 | 80937 | IRB |
| 55 | c* | (AT)5t(TA)6aataatttaaataaattatttatataataatctaattgaagtttagtaattag(TAA)4ttgaagtaaagtaataataaattgaaatttatttaaattatttatttaattaaaaaaa(TAAT)3(TAA)4*tctaattgaagtttagtaatt(A)10 | 197 | 82438 | 82634 | IRB |
| 56 | p2 | (AT)5 | 10 | 93757 | 93766 | IRB |
| 57 | p4 | (AGGT)3 | 12 | 100081 | 100092 | IRB |
| 58 | p2 | (AT)5 | 10 | 102951 | 102960 | IRB |
| 59 | p2 | (AT)5 | 10 | 103064 | 103073 | IRB |
| 60 | p1 | (T)10 | 10 | 104695 | 104704 | IRB |
| 61 | p1 | (A)10 | 10 | 104815 | 104824 | IRB |
| 62 | p1 | (A)10 | 10 | 105363 | 105372 | IRB |
| 63 | c | (A)12gatctactg(A)10cgtt(A)11 | 46 | 105723 | 105768 | IRB |
| 64 | p4 | (CAAA)3 | 12 | 107087 | 107098 | IRB |
| 65 | c | (T)11aagtatacaatagaattttttattttgtgaatccataaatatagtatagtatttatatttgtaattaattttatttg(A)15gaaattcataattattaagtaaattaagataat(TTAA)3 | 148 | 108602 | 108749 | SSC |
| 66 | p1 | (T)10 | 10 | 109173 | 109182 | SSC |
| 67 | p1 | (A)12 | 12 | 111657 | 111668 | SSC |
| 68 | p1 | (T)10 | 10 | 114168 | 114177 | SSC |
| 69 | p4 | (ATCA)3 | 12 | 114569 | 114580 | SSC |
| 70 | p4 | (TATT)3 | 12 | 116559 | 116570 | SSC |
| 71 | p2 | (TA)5 | 10 | 116976 | 116985 | SSC |
| 72 | p4 | (AAAT)3 | 12 | 118015 | 118026 | SSC |
| 73 | c | A)10tagaacgtttcgattttaatacgaagttaagttgtattcttg(T)10 | 62 | 118427 | 118488 | SSC |
| 74 | c | (AT)7tataatatatgctaaatatatggtcaatattaaatattcttatattagtaaatattctttattttattattatac(TAA)4 | 101 | 118783 | 118883 | SSC |
| 75 | p1 | (T)11 | 11 | 119053 | 119063 | SSC |
| 76 | p2 | (AT)8 | 16 | 119584 | 119599 | SSC |
| 77 | p4 | (AAAT)3 | 12 | 122081 | 122092 | SSC |

Distribution of simple sequence repeat (SSR) in the A. odoratus chloroplast genome.

| No. SSR | SSR type | SSR | Size | Start | End | Location |
| --- | --- | --- | --- | --- | --- | --- |
| 1 | p1 | (T)16 | 16 | 225 | 240 | LSC |
| 2 | c | (T)11acctcatttaaattgaatcattcattcaaaaaaaagatactaaaggttttctatagaatctatct(ATAG)3 | 88 | 1645 | 1732 | LSC |
| 3 | p1 | (T)11 | 11 | 2303 | 2313 | LSC |
| 4 | p5 | (ATATT)3 | 15 | 4627 | 4641 | LSC |
| 5 | p5 | (TTTGT)3 | 15 | 5127 | 5141 | LSC |
| 6 | p2 | (TA)5 | 10 | 6898 | 6907 | LSC |
| 7 | p1 | (A)11 | 11 | 7077 | 7087 | LSC |
| 8 | p1 | (A)11 | 11 | 7426 | 7436 | LSC |
| 9 | p2 | (AT)7 | 14 | 9593 | 9606 | LSC |
| 10 | p4 | (TTTA)3 | 12 | 10045 | 10056 | LSC |
| 11 | p2 | (AT)6 | 12 | 10449 | 10460 | LSC |
| 12 | p1 | (A)10 | 10 | 13345 | 13354 | LSC |
| 13 | p1 | (A)11 | 11 | 14104 | 14114 | LSC |
| 14 | c | (AT)5ttaaaatagaaggtattctagttctagaattcttattatattaattatatta(AT)6ta(AT)5 | 86 | 14727 | 14812 | LSC |
| 15 | p1 | (T)11 | 11 | 15186 | 15196 | LSC |
| 16 | p1 | (T)10 | 10 | 16669 | 16678 | LSC |
| 17 | c | (TA)6tttctttctatttctctattttagttagaatttttatttatataaaa(TAAT)3tattaatataaaaaaaatcgttaatctatatctatattga(AT)5 | 121 | 18593 | 18713 | LSC |
| 18 | p2 | (GA)5 | 10 | 23844 | 23853 | LSC |
| 19 | p1 | (T)11 | 11 | 24698 | 24708 | LSC |
| 20 | c | (AT)5tatagaaataat(TA)5 | 32 | 29083 | 29114 | LSC |
| 21 | p2 | (TA)6 | 12 | 29509 | 29520 | LSC |
| 22 | c | (A)15gactc(T)11 | 31 | 30247 | 30277 | LSC |
| 23 | p2 | (AT)6 | 12 | 30384 | 30395 | LSC |
| 24 | p1 | (T)10 | 10 | 30656 | 30665 | LSC |
| 25 | p1 | (A)10 | 10 | 31347 | 31356 | LSC |
| 26 | p3 | (ATA)4 | 12 | 32190 | 32201 | LSC |
| 27 | p2 | (AT)5 | 10 | 36722 | 36731 | LSC |
| 28 | p1 | (A)10 | 10 | 38208 | 38217 | LSC |
| 29 | p2 | (AT)5 | 10 | 41268 | 41277 | LSC |
| 30 | c | (A)13tggaattaaaaatattttctggaaatatccattttcccggagagatggataagatatcccgacccagtgccatcttgataccacccagaacggt(A)14 | 121 | 42647 | 42767 | LSC |
| 31 | p1 | (A)10 | 10 | 44541 | 44550 | LSC |
| 32 | p1 | (A)11 | 11 | 44867 | 44877 | LSC |
| 33 | p1 | (A)10 | 10 | 45116 | 45125 | LSC |
| 34 | p3 | (TAT)4 | 12 | 45680 | 45691 | LSC |
| 35 | c | (AT)5aatattctt(CTTA)3 | 31 | 48013 | 48043 | LSC |
| 36 | p1 | (A)11 | 11 | 48846 | 48856 | LSC |
| 37 | p2 | (TA)5 | 10 | 51674 | 51683 | LSC |
| 38 | p1 | (A)10 | 10 | 52464 | 52473 | LSC |
| 39 | p1 | (T)11 | 11 | 52584 | 52594 | LSC |
| 40 | p1 | (T)13 | 13 | 52796 | 52808 | LSC |
| 41 | p1 | (A)11 | 11 | 53278 | 53288 | LSC |
| 42 | c* | (T)10attgtattttattattttaattta(TAT)4(TATTA)3*gatatat(TA)5 | 72 | 53809 | 53880 | LSC |
| 43 | p2 | (AT)5 | 10 | 53995 | 54004 | LSC |
| 44 | p1 | (A)14 | 14 | 54999 | 55012 | LSC |
| 45 | p4 | (ATAG)3 | 12 | 55409 | 55420 | LSC |
| 46 | p3 | (TAA)4 | 12 | 55818 | 55829 | LSC |
| 47 | p3 | (ATT)4 | 12 | 57063 | 57074 | LSC |
| 48 | p3 | (ATA)5 | 15 | 57330 | 57344 | LSC |
| 49 | p1 | (A)12 | 12 | 57600 | 57611 | LSC |
| 50 | p1 | (A)10 | 10 | 61150 | 61159 | LSC |
| 51 | p1 | (A)11 | 11 | 62965 | 62975 | LSC |
| 52 | p1 | (T)12 | 12 | 63151 | 63162 | LSC |
| 53 | p1 | (T)10 | 10 | 63846 | 63855 | LSC |
| 54 | c | (ATA)5actaaagaatctaaaaaaaaga(AT)6 | 49 | 64337 | 64385 | LSC |
| 55 | p5 | (AATAA)3 | 15 | 64643 | 64657 | LSC |
| 56 | c | (A)11tttgaaaaaagcgagtcgaccactagaactactacgactgttctt(A)15 | 71 | 66092 | 66162 | LSC |
| 57 | p1 | (T)16 | 16 | 66304 | 66319 | LSC |
| 58 | p1 | (A)10 | 10 | 68460 | 68469 | LSC |
| 59 | p3 | (ATA)4 | 12 | 77816 | 77827 | LSC |
| 60 | c | (AT)5ttcattccttattttttctatctataatat(TA)5 | 50 | 78018 | 78067 | LSC |
| 61 | c | (T)10atctttcagtcaaaaagttgaagtaaaaaaaatattctgtgttc(A)12 | 66 | 78524 | 78589 | LSC |
| 62 | c | (T)11actttttttttat(A)10 | 34 | 79422 | 79455 | LSC |
| 63 | p4 | (TTTC)4 | 16 | 79761 | 79776 | LSC |
| 64 | p1 | (T)17 | 17 | 81370 | 81386 | IRB |
| 65 | c* | (AT)5gtaat(ATA)5(AT)6* | 39 | 95074 | 95112 | IRB |
| 66 | p4 | (AGGT)3 | 12 | 101470 | 101481 | IRB |
| 67 | p1 | (T)10 | 10 | 102993 | 103002 | IRB |
| 68 | p1 | (T)10 | 10 | 106045 | 106054 | IRB |
| 69 | p1 | (A10 | 10 | 106165 | 106174 | IRB |
| 70 | p1 | (A)10 | 10 | 106722 | 106731 | IRB |
| 71 | c | (A)12gatctactg(A)12gatctactg(A)12gatctactg(A)10 | 73 | 107082 | 107154 | IRB |
| 72 | p4 | (CAAA)3 | 12 | 108473 | 108484 | IRB |
| 73 | p6 | (TTCGAC)3 | 18 | 108857 | 108874 | IRB |
| 74 | p1 | (T)11 | 11 | 110009 | 110019 | SSC |
| 75 | p1 | (A)13 | 13 | 110126 | 110138 | SSC |
| 76 | p1 | (T)10 | 10 | 110602 | 110611 | SSC |
| 77 | c | (AT)5gtatagatagaataaataatataataataattata (TAT)4 | 57 | 114765 | 114821 | SSC |
| 78 | p1 | (T)10 | 10 | 115770 | 115779 | SSC |
| 79 | p4 | (ATCA)3 | 12 | 116171 | 116182 | SSC |
| 80 | p4 | (TATT)3 | 12 | 118167 | 118178 | SSC |
| 81 | p1 | (A)10 | 10 | 118467 | 118476 | SSC |
| 82 | p2 | (TA)5 | 10 | 118585 | 118594 | SSC |
| 83 | p2 | (AT)6 | 12 | 119611 | 119622 | SSC |
| 84 | p3 | (TAT)4 | 12 | 120113 | 120124 | SSC |
| 85 | p3 | (TAA) 4 | 12 | 120245 | 120256 | SSC |
| 86 | p1 | (T)11 | 11 | 120420 | 120430 | SSC |
| 87 | p1 | (T)10 | 10 | 121201 | 121210 | SSC |
