# Supplementary material for: Characterization and comparative analysis of the complete plastid genomes of four Astragalus species
Source: PLoS One. 2023 May 23;18(5):e0286083. doi: 10.1371/journal.pone.0286083 (PMC10204964; doi:10.1371/journal.pone.0286083)
Supplement: S7 Table — (DOCX) [file pone.0286083.s007.docx]

**S7 Table.** The Ka, Ks and Ka/Ks ratio of Astragalus species chloroplast genomes for individual genes and regions.

| Gene groups | gene | Ka | Ks | Ka/Ks | Region |
| --- | --- | --- | --- | --- | --- |
| Large subunit of ribosomal proteins | *rpl*33 | 0.010759 | 0.025973 | 0.414237 | LSC |
|  | *rpl*20 | 0.021498 | 0.048439 | 0.443815 | LSC |
|  | *rpl*36 | 0.004697 | 0.048702 | 0.096443 | LSC |
|  | *rpl*14 | 0.003967 | 0.016456 | 0.241067 | LSC |
|  | *rpl*16 | 0.010384 | 0.010667 | 0.973469 | LSC |
|  | *rpl*2 | 0.006626 | 0.032228 | 0.205597 | IR |
|  | *rpl*23 | 0.014739 | 0.025215 | 0.584533 | IR |
|  | *rpl*32 | 0.013513 | 0.042951 | 0.314614 | SSC |
| Small subunit of ribosomal proteins | *rps*2 | 0.010109 | 0.017306 | 0.584132 | LSC |
|  | *rps*14 | 0.009631 | 0.044573 | 0.021607 | LSC |
|  | *rps*4 | 0.007017 | 0.014413 | 0.486852 | LSC |
|  | *rps*18 | 0.016624 | 0.023027 | 0.721935 | LSC |
|  | *rps*11 | 0.015361 | 0.010091 | 1.522247 | LSC |
|  | *rps*8 | 0.002997 | 0.028188 | 0.106321 | LSC |
|  | *rps*3 | 0.008152 | 0.020211 | 0.403344 | LSC |
|  | *rps*19 | 0.009189 | 0.027946 | 0.328812 | LSC |
|  | *rps*7 | 0.014445 | 0.020902 | 0.691082 | IR |
|  | *rps*12 | 0.000000 | 0.014604 | 0.000000 | LSC/IR |
|  | *rps*15 | 0.010119 | 0.009781 | 1.034556 | SSC |
| DNA-dependent RNA polymerase | *rpo*C2 | 0.00573 | 0.021514 | 0.266338 | LSC |
|  | *rpo*C1 | 0.003595 | 0.021849 | 0.164538 | LSC |
|  | *rpo*B | 0.002939 | 0.023055 | 0.127477 | LSC |
|  | *rpo*A | 0.003673 | 0.018977 | 0.19355 | LSC |
| Photosystem I | *psa*B | 0.000862 | 0.015859 | 0.054353 | LSC |
|  | *psa*A | 0.000658 | 0.019072 | 0.0345 | LSC |
|  | *psa*I | 0.008073 | 0.015818 | 0.510367 | LSC |
|  | *psa*J | 0.006021 | 0.042731 | 0.140904 | LSC |
|  | *psa*C | 0.00054 | 0.018089 | 0.029852 | SSC |
| Photosystem II | *psb*A | 0.00012 | 0.015028 | 0.007985 | LSC |
|  | *psb*K | 0.00142 | 0.011354 | 0.125066 | LSC |
|  | *psb*I | 0.001239 | 0.022694 | 0.054595 | LSC |
|  | *psb*M | 0.007881 | 0.016955 | 0.464818 | LSC |
|  | *psb*D | 0.000227 | 0.013609 | 0.01668 | LSC |
|  | *psb*C | 0.001114 | 0.011427 | 0.097488 | LSC |
|  | *psb*Z | 0.005204 | 0.065565 | 0.079371 | LSC |
|  | *psb*J | 0.00336 | 0.017166 | 0.195735 | LSC |
|  | *psb*L | 0.000000 | 0.014803 | 0.000000 | LSC |
|  | *psb*F | 0.00115 | 0.000000 | indeterm | LSC |
|  | *psb*E | 0.00052 | 0.003322 | 0.156532 | LSC |
|  | *psb*B | 0.000979 | 0.017056 | 0.057399 | LSC |
|  | *psb*T | 0.000000 | 0.015843 | 0.000000 | LSC |
|  | *psb*N | 0.000000 | 0.012563 | 0.000000 | LSC |
|  | *psb*H | 0.000000 | 0.005226 | 0.000000 | LSC |
| NADH dehydrogenase | *ndh*J | 0.000541 | 0.019945 | 0.027124 | LSC |
|  | *ndh*K | 0.003429 | 0.011264 | 0.304421 | LSC |
|  | *ndh*C | 0.003413 | 0.018596 | 0.183534 | LSC |
|  | *ndh*B | 0.002629 | 0.011746 | 0.22382 | IR |
|  | *ndh*F | 0.005297 | 0.018292 | 0.28958 | SSC |
|  | *ndh*D | 0.002142 | 0.012216 | 0.175343 | SSC |
|  | *ndh*E | 0.004284 | 0.030642 | 0.139808 | SSC |
|  | *ndh*G | 0.0025 | 0.012724 | 0.196479 | SSC |
|  | *ndh*I | 0.001265 | 0.007867 | 0.160798 | SSC |
|  | *ndh*A | 0.002465 | 0.0269 | 0.091635 | SSC |
|  | *ndh*H | 0.001204 | 0.019136 | 0.062918 | SSC |
| Cytochrome b/f complex | *pet*N | 0.000000 | 0.015886 | 0.000000 | LSC |
|  | *pet*A | 0.002799 | 0.034993 | 0.079987 | LSC |
|  | *pet*L | 0.007712 | 0.000000 | indeterm | LSC |
|  | *pet*G | 0.000000 | 0.036984 | 0.000000 | LSC |
|  | *pet*B | 0.000591 | 0.019389 | 0.030481 | LSC |
|  | *pet*D | 0.001518 | 0.023124 | 0.065646 | LSC |
| ATP synthase | *atp*A | 0.005231 | 0.031279 | 0.167236 | LSC |
|  | *atp*F | 0.004717 | 0.008258 | 0.571203 | LSC |
|  | *atp*H | 0.00252 | 0.037258 | 0.067636 | LSC |
|  | *atp*I | 0.002946 | 0.022184 | 0.132798 | LSC |
|  | *atp*E | 0.006764 | 0.007092 | 0.95375 | LSC |
|  | *atp*B | 0.004149 | 0.011828 | 0.350777 | LSC |
| RubisCo large subunit | *rbc*L | 0.008372 | 0.028378 | 0.295017 | LSC |
| Maturase K | *mat*K | 0.00804 | 0.022542 | 0.356667 | LSC |
| Envelope membrane protein | *cem*A | 0.006812 | 0.016107 | 0.422921 | LSC |
| Subunit of acetyl-CoAcarboxylase | *acc*D | 0.070763 | 0.06515 | 1.086155 | LSC |
| C-type cytochrome synthesis gene | *ccs*A | 0.007896 | 0.032338 | 0.24417 | SSC |
| Protease | *clp*P | 0.107069 | 0.078252 | 1.368258 | LSC |
| Conserved hypothetical chloroplast open reading frames | *ycf*3 | 0.00026 | 0.030057 | 0.00865 | LSC |
|  | *ycf*4 | 0.002069 | 0.026855 | 0.077043 | LSC |
|  | *ycf*2 | 0.028331 | 0.033692 | 0.840882 | IR |
|  | *ycf*1 | 0.050006 | 0.035881 | 1.393662 | SSC |

Ka non-synonymous substitution, Ks synonymous substitution, LSC Large Single Copy, SSC Small Single Copy, IR Inverted Repeat.
