# Supplementary material for: Characterization and comparative analysis of the complete plastid genomes of four Astragalus species
Source: PLoS One. 2023 May 23;18(5):e0286083. doi: 10.1371/journal.pone.0286083 (PMC10204964; doi:10.1371/journal.pone.0286083)
Supplement: S8 Table — (DOCX) [file pone.0286083.s008.docx]

**S8 Table**. Prediction of RNA editing sites in chloroplast genes of *Astragalus* species.

Prediction of RNA editing sites in chloroplast genes of *A. arrectus*

| Gene | Nucleotide position | Amino acid position | RNA editing effect | Score |
| --- | --- | --- | --- | --- |
| *acc*D | 251  322  1171 | 84  108  391 | CCG (P) => CTG (L)  CAT (H) => TAT (Y)  CCG (P) => TCG (S) | 1.00  1.00  1.00 |
| *atp*I | 76 | 26 | CTC (L) => TTC (F) | 0.86 |
| *ccs*A | 137  514 | 46  172 | ACA (T) => ATA (I)  CTT (L) => TTT (F) | 1.00  0.86 |
| *clp*P | 128 | 43 | ACA (T) => ATA (I) | 0.86 |
| *mat*K | 979  1178 | 327  393 | CTT (L) => TTT (F)  TCA (S) => TTA (L) | 0.86  0.86 |
| *ndh*A | 137  341  1073 | 46  114  358 | GCA (A) => GTA (V)  TCA (S) => TTA (L)  TCT (S) => TTT (F) | 1.00  1.00  1.00 |
| *ndh*B | 28  746  836  1487 | 10  249  279  496 | CTC (L) => TTC (F)  TCT (S) => TTT (F)  TCA (S) => TTA (L)  CCA (P) => CTA (L) | 1.00  1.00  1.00  1.00 |
| *ndh*D | 2  674  878  1405 | 1  225  293  469 | ACG (T) => ATG (M)  TCG (S) => TTG (L)  TCA (S) => TTA (L)  CTT (L) => TTT (F) | 1.00  1.00  1.00  0.80 |
| *ndh*F | 13  290  586  1172 | 5  97  196  391 | CAT (H) => TAT (Y)  TCA (S) => TTA (L)  CTT (L) => TTT (F)  GCG (A) => GTG (V) | 1.00  1.00  0.80  0.80 |
| *ndh*G | 166  314  385 | 56  105  129 | CAT (H) => TAT (Y)  ACA (T) => ATA (I)  CCA (P) => TCA (S) | 0.80  0.80  0.80 |
| *pet*B | 611 | 204 | CCA (P) => CTA (L) | 1.00 |
| *psa*B | 1456 | 486 | CTC (L) => TTC (F) | 1.00 |
| *rpl*2 | 593 | 198 | GCA (A) => GTA (V) | 0.86 |
| *rpl*20 | 61 | 21 | CTT (L) => TTT (F) | 0.86 |
| *rpo*B | 226  338  551  566  718  2000  2426 | 76  113  184  189  240  667  809 | CAT (H) => TAT (Y)  TCT (S) => TTT (F)  TCA (S) => TTA (L)  TCG (S) => TTG (L)  CCT (P) => TCT (S)  TCT (S) => TTT (F)  TCA (S) => TTA (L) | 1.00  1.00  1.00  1.00  1.00  1.00  0.86 |
| *rpo*C1 | 127  1556 | 43  519 | CTT (L) => TTT (F)  ACG (T) => ATG (M) | 0.86  0.86 |
| *ycf*3 | 362 | 121 | ACT (T) => ATT (I) | 1.00 |

Prediction of RNA editing sites in chloroplast genes of *A. bhotanensis*

| Gene | Nucleotide position | Amino acid position | RNA editing effect | Score |
| --- | --- | --- | --- | --- |
| *acc*D | 209  283  1075 | 70  95  359 | CCG (P) => CTG (L)  CAT (H) => TAT (Y)  CCG (P) => TCG (S) | 1.00  1.00  1.00 |
| *atp*I | 76 | 26 | CTC (L) => TTC (F) | 0.86 |
| *ccs*A | 137  514 | 46  172 | ACA (T) => ATA (I)  CTT (L) => TTT (F) | 1.00  0.86 |
| *clp*P | 313 | 105 | CTT (L) => TTT (F) | 1.00 |
| *mat*K | 988  1187 | 330  396 | CTT (L) => TTT (F)  TCA (S) => TTA (L) | 0.86  0.86 |
| *ndh*A | 137  341  1073 | 46  114  358 | GCA (A) => GTA (V)  TCA (S) => TTA (L)  TCT (S) => TTT (F) | 1.00  1.00  1.00 |
| *ndh*B | 557  692  782  1433 | 186  231  261  478 | TCA (S) => TTA (L)  TCT (S) => TTT (F)  TCA (S) => TTA (L)  CCA (P) => CTA (L) | 0.80  1.00  1.00  1.00 |
| *ndh*D | 2  674  1405 | 1  225  469 | ACG (T) => ATG (M)  TCG (S) => TTG (L)  CTT (L) => TTT (F) | 1.00  1.00  0.80 |
| *ndh*F | 13  290  586  1172 | 5  97  196  391 | CAT (H) => TAT (Y)  TCA (S) => TTA (L)  CTT (L) => TTT (F)  GCG (A) => GTG (V) | 1.00  1.00  0.80  0.80 |
| *ndh*G | 166  314  385 | 56  105  129 | CAT (H) => TAT (Y)  ACA (T) => ATA (I)  CCA (P) => TCA (S) | 0.80  0.80  0.80 |
| *pet*B | 611 | 204 | CCA (P) => CTA (L) | 1.00 |
| *psa*B | 1456 | 486 | CTC (L) => TTC (F) | 1.00 |
| *rpl*2 | 593 | 198 | GCA (A) => GTA (V) | 0.86 |
| *rpo*A | 1001 | 334 | TCA (S) => TTA (L) | 0.83 |
| *rpo*B | 226  338  551  566  718  2000  2426 | 76  113  184  189  240  667  809 | CAT (H) => TAT (Y)  TCT (S) => TTT (F)  TCA (S) => TTA (L)  TCG (S) => TTG (L)  CCT (P) => TCT (S)  TCT (S) => TTT (F)  TCA (S) => TTA (L) | 1.00  1.00  1.00  1.00  1.00  1.00  0.86 |
| *rpo*C1 | 127  1556 | 43  519 | CTT (L) => TTT (F)  ACG (T) => ATG (M) | 0.86  0.86 |
| *ycf*3 | 362 | 121 | ACT (T) => ATT (I) | 1.00 |

Prediction of RNA editing sites in chloroplast genes of *A. calycosus*

| Gene | Nucleotide position | Amino acid position | RNA editing effect | Score |
| --- | --- | --- | --- | --- |
| *acc*D | 604  895 | 202  299 | CAT (H) => TAT (Y)  CCT (P) => TCT (S) | 1.00  1.00 |
| *atp*I | 76 | 26 | CTC (L) => TTC (F) | 0.86 |
| *ccs*A | 137  514 | 46  172 | ACA (T) => ATA (I)  CTT (L) => TTT (F) | 1.00  0.86 |
| *clp*P | 313 | 105 | CTT (L) => TTT (F) | 1.00 |
| *mat*K | 988  1187 | 330  396 | CTT (L) => TTT (F)  TCA (S) => TTA (L) | 0.86  0.86 |
| *ndh*A | 137  341  1073 | 46  114  358 | GCA (A) => GTA (V)  TCA (S) => TTA (L)  TCT (S) => TTT (F) | 1.00  1.00  1.00 |
| *ndh*B | 28  746  836  1487 | 10  249  279  496 | CTC (L) => TTC (F)  TCT (S) => TTT (F)  TCA (S) => TTA (L)  CCA (P) => CTA (L) | 0.80  1.00  1.00  1.00 |
| *ndh*D | 2  674  878  1405 | 1  225  293  469 | ACG (T) => ATG (M)  TCG (S) => TTG (L)  TCA (S) => TTA (L)  CTT (L) => TTT (F) | 1.00  1.00  1.00  0.80 |
| *ndh*F | 13  290  586  1172 | 5  97  196  391 | CAT (H) => TAT (Y)  TCA (S) => TTA (L)  CTT (L) => TTT (F)  GCG (A) => GTG (V) | 1.00  1.00  0.80  0.80 |
| *ndh*G | 166  314  385 | 56  105  129 | CAT (H) => TAT (Y)  ACA (T) => ATA (I)  CCA (P) => TCA (S) | 0.80  0.80  0.80 |
| *pet*B | 611 | 204 | CCA (P) => CTA (L) | 1.00 |
| *psa*B | 1456 | 486 | CTC (L) => TTC (F) | 1.00 |
| *rpl*2 | 593 | 198 | GCA (A) => GTA (V) | 0.86 |
| *rpo*B | 226  338  551  566  718  2000  2426 | 76  113  184  189  240  667  809 | CAT (H) => TAT (Y)  TCT (S) => TTT (F)  TCA (S) => TTA (L)  TCG (S) => TTG (L)  CCT (P) => TCT (S)  TCT (S) => TTT (F)  TCA (S) => TTA (L) | 1.00  1.00  1.00  1.00  1.00  1.00  0.86 |
| *rpo*C1 | 1556 | 519 | ACG (T) => ATG (M) | 0.86 |
| *rps*2 | 607 | 203 | CCG (P) => TCG (S) | 0.86 |
| *ycf*3 | 362 | 121 | ACT (T) => ATT (I) | 1.00 |

Prediction of RNA editing sites in chloroplast genes of *A. flexuosus*

| Gene | Nucleotide position | Amino acid position | RNA editing effect | Score |
| --- | --- | --- | --- | --- |
| *acc*D | 230  301  1093 | 77  101  365 | CCG (P) => CTG (L) CAT (H) => TAT (Y)  CCG (P) => TCG (S) | 1.00  1.00  1.00 |
| *atp*I | 76 | 26 | CTC (L) => TTC (F) | 0.86 |
| *ccs*A | 137  514 | 46  172 | ACA (T) => ATA (I)  CTT (L) => TTT (F) | 1.00  0.86 |
| *clp*P | 325 | 109 | CTT (L) => TTT (F) | 1.00 |
| *mat*K | 988  1187 | 330  396 | CTT (L) => TTT (F)  TCA (S) => TTA (L) | 0.86  0.86 |
| *ndh*A | 137  341  1073 | 46  114  358 | GCA (A) => GTA (V)  TCA (S) => TTA (L)  TCT (S) => TTT (F) | 1.00  1.00  1.00 |
| *ndh*B | 28  746  836  1487 | 10  249  279  496 | CTC (L) => TTC (F)  TCT (S) => TTT (F)  TCA (S) => TTA (L)  CCA (P) => CTA (L) | 0.80  1.00  1.00  1.00 |
| *ndh*D | 2  674  878  1405 | 1  225  293  469 | ACG (T) => ATG (M)  TCG (S) => TTG (L)  TCA (S) => TTA (L)  CTT (L) => TTT (F) | 1.00  1.00  1.00  0.80 |
| *ndh*F | 13  290  586  1172 | 5  97  196  391 | CAT (H) => TAT (Y)  TCA (S) => TTA (L)  CTT (L) => TTT (F)  GCG (A) => GTG (V) | 1.00  1.00  0.80  0.80 |
| *ndh*G | 166  314  385 | 56  105  129 | CAT (H) => TAT (Y)  ACA (T) => ATA (I)  CCA (P) => TCA (S) | 0.80  0.80  0.80 |
| *pet*B | 611 | 204 | CCA (P) => CTA (L) | 1.00 |
| *psa*B | 1456 | 486 | CTC (L) => TTC (F) | 1.00 |
| *rpl*2 | 593 | 198 | GCA (A) => GTA (V) | 0.86 |
| *rpl*20 | 236 | 79 | GCA (A) => GTA (V) | 0.86 |
| *rpo*B | 226  338  551  566  718  2000  2426 | 76  113  184  189  240  667  809 | CAT (H) => TAT (Y)  TCT (S) => TTT (F)  TCA (S) => TTA (L)  TCG (S) => TTG (L)  CCT (P) => TCT (S)  TCT (S) => TTT (F)  TCA (S) => TTA (L) | 1.00  1.00  1.00  1.00  1.00  1.00  0.86 |
| *rpo*C1 | 127  1556 | 43  519 | CTT (L) => TTT (F)  ACG (T) => ATG (M) | 0.86  0.86 |
| *ycf*3 | 362 | 121 | ACT (T) => ATT (I) | 1.00 |

Prediction of RNA editing sites in chloroplast genes of *A. galactites*

| Gene | Nucleotide position | Amino acid position | RNA editing effect | Score |
| --- | --- | --- | --- | --- |
| *acc*D | 209  283  1069 | 70  95  357 | CCG (P) => CTG (L) CAT (H) => TAT (Y)  CCG (P) => TCG (S) | 1.00  1.00  1.00 |
| *atp*I | 76 | 26 | CTC (L) => TTC (F) | 0.86 |
| *ccs*A | 137  514 | 46  172 | ACA (T) => ATA (I)  CTT (L) => TTT (F) | 1.00  0.86 |
| *clp*P | 313 | 105 | CTT (L) => TTT (F) | 1.00 |
| *mat*K | 988  1187 | 330  396 | CTT (L) => TTT (F)  TCA (S) => TTA (L) | 0.86  0.86 |
| *ndh*A | 137  341  1073 | 46  114  358 | GCA (A) => GTA (V)  TCA (S) => TTA (L)  TCT (S) => TTT (F) | 1.00  1.00  1.00 |
| *ndh*B | 557  692  782  1433 | 186  231  261  478 | TCA (S) => TTA (L)  TCT (S) => TTT (F)  TCA (S) => TTA (L)  CCA (P) => CTA (L) | 0.80  1.00  1.00  1.00 |
| *ndh*D | 5  677  881  1408 | 2  226  294  470 | ACG (T) => ATG (M)  TCG (S) => TTG (L)  TCA (S) => TTA (L)  CTT (L) => TTT (F) | 1.00  1.00  1.00  0.80 |
| *ndh*F | 13  290  586  1172 | 5  97  196  391 | CAT (H) => TAT (Y)  TCA (S) => TTA (L)  CTT (L) => TTT (F)  GCG (A) => GTG (V) | 1.00  1.00  0.80  0.80 |
| *ndh*G | 166  314  385 | 56  105  129 | CAT (H) => TAT (Y)  ACA (T) => ATA (I)  CCA (P) => TCA (S) | 0.80  0.80  0.80 |
| *pet*B | 611 | 204 | CCA (P) => CTA (L) | 1.00 |
| *psa*B | 1456 | 486 | CTC (L) => TTC (F) | 1.00 |
| *rpo*B | 226  338  551  566  718  2000  2426 | 76  113  184  189  240  667  809 | CAT (H) => TAT (Y)  TCT (S) => TTT (F)  TCA (S) => TTA (L)  TCG (S) => TTG (L)  CCT (P) => TCT (S)  TCT (S) => TTT (F)  TCA (S) => TTA (L) | 1.00  1.00  1.00  1.00  1.00  1.00  0.86 |
| *rpo*C1 | 127  1556 | 43  519 | CTT (L) => TTT (F)  ACG (T) => ATG (M) | 0.86  0.86 |
| *ycf*3 | 362 | 121 | ACT (T) => ATT (I) | 1.00 |

Prediction of RNA editing sites in chloroplast genes of *A. gypsodes*

| Gene | Nucleotide position | Amino acid position | RNA editing effect | Score |
| --- | --- | --- | --- | --- |
| *acc*D | 337  871 | 113  291 | CAT (H) => TAT (Y)  CCG (P) => TCG (S) | 0.80  1.00 |
| *atp*I | 76 | 26 | CTC (L) => TTC (F) | 0.86 |
| *ccs*A | 137  514 | 46  172 | ACA (T) => ATA (I)  CTT (L) => TTT (F) | 1.00  0.86 |
| *clp*P | 128  532 | 43  178 | ACA (T) => ATA (I)  CTT (L) => TTT (F) | 0.86  1.00 |
| *mat*K | 988  1187 | 330  396 | CTT (L) => TTT (F)  TCA (S) => TTA (L) | 0.86  0.86 |
| *ndh*A | 137  341  1073 | 46  114  358 | GCA (A) => GTA (V)  TCA (S) => TTA (L)  TCT (S) => TTT (F) | 1.00  1.00  1.00 |
| *ndh*B | 28  746  836  1481 | 10  249  279  494 | CTC (L) => TTC (F) TCT (S) => TTT (F)  TCA (S) => TTA (L)  CCA (P) => CTA (L) | 0.80  1.00  1.00  1.00 |
| *ndh*D | 2  674  878  1405 | 1  225  293  469 | ACG (T) => ATG (M)  TCG (S) => TTG (L)  TCA (S) => TTA (L)  CTT (L) => TTT (F) | 1.00  1.00  1.00  0.80 |
| *ndh*F | 13  290  586  1172 | 5  97  196  391 | CAT (H) => TAT (Y)  TCA (S) => TTA (L)  CTT (L) => TTT (F)  GCG (A) => GTG (V) | 1.00  1.00  0.80  0.80 |
| *ndh*G | 166  314  385 | 56  105  129 | CAT (H) => TAT (Y)  ACA (T) => ATA (I)  CCA (P) => TCA (S) | 0.80  0.80  0.80 |
| *pet*B | 611 | 204 | CCA (P) => CTA (L) | 1.00 |
| *psa*B | 1456 | 486 | CTC (L) => TTC (F) | 1.00 |
| *rpl*2 | 593 | 198 | GCA (A) => GTA (V) | 0.86 |
| *rpo*B | 226  338  551  566  718  2000  2426 | 76  113  184  189  240  667  809 | CAT (H) => TAT (Y)  TCT (S) => TTT (F)  TCA (S) => TTA (L)  TCG (S) => TTG (L)  CCT (P) => TCT (S)  TCT (S) => TTT (F)  TCA (S) => TTA (L) | 1.00  1.00  1.00  1.00  1.00  1.00  0.86 |
| *rpo*C1 | 127  1556 | 43  519 | CTT (L) => TTT (F)  ACG (T) => ATG (M) | 0.86  0.86 |
| *ycf*3 | 362 | 121 | ACT (T) => ATT (I) | 1.00 |

Prediction of RNA editing sites in chloroplast genes of *A. iranicus*

| Gene | Nucleotide position | Amino acid position | RNA editing effect | Score |
| --- | --- | --- | --- | --- |
| *acc*D | 215  286  1072 | 72  96  358 | CCG (P) => CTG (L)  CAT (H) => TAT (Y)  CCG (P) => TCG (S) | 1.00  1.00  1.00 |
| *atp*F | 461 | 154 | ACT (T) => ATT (I) | 1.00 |
| *atp*I | 76 | 26 | CTC (L) => TTC (F) | 0.86 |
| *ccs*A | 137  514 | 46  172 | ACA (T) => ATA (I)  CTT (L) => TTT (F) | 1.00  0.86 |
| *clp*P | 187  383  722 | 63  128  241 | CTC (L) => TTC (F) CCA (P) => CTA (L)  GCT (A) => GTT (V) | 1.00  0.86  0.86 |
| *mat*K | 988  1187 | 330  396 | CTT (L) => TTT (F)  TCA (S) => TTA (L) | 0.86  0.86 |
| *ndh*A | 137  341 | 46  114 | GCA (A) => GTA (V)  TCA (S) => TTA (L) | 1.00  1.00 |
| *ndh*B | 557  692  878  986  1340  1414  1418  1451  2012 | 186  231  293  329  447  472  473  484  671 | TCA (S) => TTA (L) TCT (S) => TTT (F)  TCA (S) => TTA (L)  TCG (S) => TTG (L)  CCG (P) => CTG (L)  CTC (L) => TTC (F)  CCA (P) => CTA (L)  CCA (P) => CTA (L)  ACC (T) => ATC (I) | 0.80  1.00  1.00  1.00  1.00  1.00  1.00  1.00  1.00 |
| *ndh*D | 2  674  878  1405 | 1  225  293  469 | ACG (T) => ATG (M)  TCG (S) => TTG (L)  TCA (S) => TTA (L)  CTT (L) => TTT (F) | 1.00  1.00  1.00  0.80 |
| *ndh*F | 13  241  290 | 5  81  97 | CAT (H) => TAT (Y)  CTT (L) => TTT (F)  TCA (S) => TTA (L) | 1.00  1.00  1.00 |
| *ndh*G | 166  314  385 | 56  105  129 | CAT (H) => TAT (Y)  ACA (T) => ATA (I)  CCA (P) => TCA (S) | 0.80  0.80  0.80 |
| *pet*B | 1418 | 473 | CCA (P) => CTA (L) | 1.00 |
| *pet*D | 1039  1099  1166 | 347  367  389 | CTT (L) => TTT (F)  CTT (L) => TTT (F)  CCT (P) => CTT (L) | 1.00  1.00  1.00 |
| *psa*B | 1456 | 486 | CTC (L) => TTC (F) | 1.00 |
| *rpl*2 | 593 | 198 | GCA (A) => GTA (V) | 0.86 |
| *rpo*B | 226  338  551  566  718  2000  2426 | 76  113  184  189  240  667  809 | CAT (H) => TAT (Y)  TCT (S) => TTT (F)  TCA (S) => TTA (L)  TCG (S) => TTG (L)  CCT (P) => TCT (S)  TCT (S) => TTT (F)  TCA (S) => TTA (L) | 1.00  1.00  1.00  1.00  1.00  1.00  0.86 |
| *rpo*C1 | 127  2276 | 43  759 | CTT (L) => TTT (F)  ACG (T) => ATG (M) | 0.86  0.86 |

Prediction of RNA editing sites in chloroplast genes of *A. laxmannii*

| Gene | Nucleotide position | Amino acid position | RNA editing effect | Score |
| --- | --- | --- | --- | --- |
| *acc*D | 209  283  1069 | 70  95  357 | CCG (P) => CTG (L) CAT (H) => TAT (Y)  CCG (P) => TCG (S) | 1.00  1.00  1.00 |
| *atp*I | 76 | 26 | CTC (L) => TTC (F) | 0.86 |
| *ccs*A | 137  514 | 46  172 | ACA (T) => ATA (I)  CTT (L) => TTT (F) | 1.00  0.86 |
| *clp*P | 313 | 105 | CTT (L) => TTT (F) | 1.00 |
| *mat*K | 988  1187 | 330  396 | CTT (L) => TTT (F)  TCA (S) => TTA (L) | 0.86  0.86 |
| *ndh*A | 137  341  1073 | 46  114  358 | GCA (A) => GTA (V)  TCA (S) => TTA (L)  TCT (S) => TTT (F) | 1.00  1.00  1.00 |
| *ndh*B | 557  692  782  1433 | 186  231  261  478 | TCA (S) => TTA (L)  TCT (S) => TTT (F)  TCA (S) => TTA (L)  CCA (P) => CTA (L) | 0.80  1.00  1.00  1.00 |
| *ndh*D | 5  677  881  1408 | 2  226  294  470 | ACG (T) => ATG (M)  TCG (S) => TTG (L)  TCA (S) => TTA (L)  CTT (L) => TTT (F) | 1.00  1.00  1.00  0.80 |
| *ndh*F | 13  290  586  1172 | 5  97  196  391 | CAT (H) => TAT (Y)  TCA (S) => TTA (L)  CTT (L) => TTT (F)  GCG (A) => GTG (V) | 1.00  1.00  0.80  0.80 |
| *ndh*G | 166  314  385 | 56  105  129 | CAT (H) => TAT (Y)  ACA (T) => ATA (I)  CCA (P) => TCA (S) | 0.80  0.80  0.80 |
| *pet*B | 611 | 204 | CCA (P) => CTA (L) | 1.00 |
| *psa*B | 1456 | 486 | CTC (L) => TTC (F) | 1.00 |
| *rpo*B | 226  338  551  566  718  2000  2426 | 76  113  184  189  240  667  809 | CAT (H) => TAT (Y)  TCT (S) => TTT (F)  TCA (S) => TTA (L)  TCG (S) => TTG (L)  CCT (P) => TCT (S)  TCT (S) => TTT (F)  TCA (S) => TTA (L) | 1.00  1.00  1.00  1.00  1.00  1.00  0.86 |
| *rpo*C1 | 127  1556 | 43  519 | CTT (L) => TTT (F)  ACG (T) => ATG (M) | 0.86  0.86 |
| *ycf*3 | 362 | 121 | ACT (T) => ATT (I) | 1.00 |

Prediction of RNA editing sites in chloroplast genes of *A. macropelmatus*

| Gene | Nucleotide position | Amino acid position | RNA editing effect | Score |
| --- | --- | --- | --- | --- |
| *acc*D | 158  215  286 | 53  72  96 | TCT (S) => TTT (F) CCG (P) => CTG (L)  CAT (H) => TAT (Y) | 1.00  1.00  1.00 |
| *atp*F | 986 | 329 | TCG (S) => TTG (L) | 1.00 |
| *atp*I | 76 | 26 | CTC (L) => TTC (F) | 0.86 |
| *ccs*A | 137  514 | 46  172 | ACA (T) => ATA (I)  CTT (L) => TTT (F) | 1.00  0.86 |
| *mat*K | 988  1187 | 330  396 | CTT (L) => TTT (F)  TCA (S) => TTA (L) | 0.86  0.86 |
| *ndh*A | 137  341  2318 | 46  114  773 | GCA (A) => GTA (V)  TCA (S) => TTA (L)  TCT (S) => TTT (F) | 1.00  1.00  1.00 |
| *ndh*B | 557  692  878  986  1136  1315  1829  2117 | 186  231  293  329  379  439  610  706 | TCA (S) => TTA (L)  TCT (S) => TTT (F)  TCA (S) => TTA (L)  TCG (S) => TTG (L)  ACC (T) => ATC (I)  CTT (L) => TTT (F)  CCT (P) => CTT (L)  ACC (T) => ATC (I) | 0.80  1.00  1.00  1.00  1.00  1.00  1.00  1.00 |
| *ndh*D | 2  674  878  1405 | 1  225  293  469 | ACG (T) => ATG (M)  TCG (S) => TTG (L)  TCA (S) => TTA (L)  CTT (L) => TTT (F) | 1.00  1.00  1.00  0.80 |
| *ndh*F | 13  241  290  586  1172 | 5  81  97  196  391 | CAT (H) => TAT (Y)  CTT (L) => TTT (F)  TCA (S) => TTA (L)  CTT (L) => TTT (F)  GCG (A) => GTG (V) | 1.00  1.00  1.00  0.80  0.80 |
| *ndh*G | 166  314  385 | 56  105  129 | CAT (H) => TAT (Y)  ACA (T) => ATA (I)  CCA (P) => TCA (S) | 0.80  0.80  0.80 |
| *pet*B | 307 | 103 | CAT (H) => TAT (Y) | 1.00 |
| *psa*B | 1456 | 486 | CTC (L) => TTC (F) | 1.00 |
| *rpo*B | 338  551  566  718  2000  2426 | 113  184  189  240  667  809 | TCT (S) => TTT (F)  TCA (S) => TTA (L)  TCG (S) => TTG (L)  CCT (P) => TCT (S)  TCT (S) => TTT (F)  TCA (S) => TTA (L) | 1.00  1.00  1.00  1.00  1.00  0.86 |
| *rpo*C1 | 127  2210 | 43  737 | CTT (L) => TTT (F)  ACG (T) => ATG (M) | 0.86  0.86 |
| *rps*2 | 607 | 203 | CCG (P) => TCG (S) | 0.86 |
| *ycf*3 | 724  1232 | 242  411 | CAT (H) => TAT (Y)  CCT (P) => CTT (L) | 1.00  1.00 |

Prediction of RNA editing sites in chloroplast genes of *A. membranaceus*

| Gene | Nucleotide position | Amino acid position | RNA editing effect | Score |
| --- | --- | --- | --- | --- |
| *acc*D | 209  280  1078 | 70  94  360 | CCG (P) => CTG (L)  CAT (H) => TAT (Y) CCG (P) => TCG (S) | 1.00  1.00  1.00 |
| *atp*I | 76 | 26 | CTC (L) => TTC (F) | 0.86 |
| *ccs*A | 137  514 | 46  172 | ACA (T) => ATA (I)  CTT (L) => TTT (F) | 1.00  0.86 |
| *clp*P | 313 | 105 | CTT (L) => TTT (F) | 1.00 |
| *mat*K | 994  1193 | 332  398 | CTT (L) => TTT (F)  TCA (S) => TTA (L) | 0.86  0.86 |
| *ndh*A | 137  341  1073 | 46  114  358 | GCA (A) => GTA (V)  TCA (S) => TTA (L)  TCT (S) => TTT (F) | 1.00  1.00  1.00 |
| *ndh*B | 557  692  782  1201  1433 | 186  231  261  401  478 | TCA (S) => TTA (L)  TCT (S) => TTT (F)  TCA (S) => TTA (L)  CAT (H) => TAT (Y)  CCA (P) => CTA (L) | 0.80  1.00  1.00  1.00  1.00 |
| *ndh*D | 5  677  881  1408 | 2  226  294  470 | ACG (T) => ATG (M)  TCG (S) => TTG (L)  TCA (S) => TTA (L)  CTT (L) => TTT (F) | 1.00  1.00  1.00  0.80 |
| *ndh*F | 13  241  290  586  1172 | 5  81  97  196  391 | CAT (H) => TAT (Y)  CTT (L) => TTT (F)  TCA (S) => TTA (L)  CTT (L) => TTT (F)  GCG (A) => GTG (V) | 1.00  1.00  1.00  0.80  0.80 |
| *ndh*G | 166  314  385 | 56  105  129 | CAT (H) => TAT (Y)  ACA (T) => ATA (I)  CCA (P) => TCA (S) | 0.80  0.80  0.80 |
| *pet*B | 611 | 204 | CCA (P) => CTA (L) | 1.00 |
| *psa*B | 1456 | 486 | CTC (L) => TTC (F) | 1.00 |
| *rpo*B | 338  551  566  718  2000  2426 | 113  184  189  240  667  809 | TCT (S) => TTT (F)  TCA (S) => TTA (L)  TCG (S) => TTG (L)  CCT (P) => TCT (S)  TCT (S) => TTT (F)  TCA (S) => TTA (L) | 1.00  1.00  1.00  1.00  1.00  0.86 |
| *rpo*C1 | 127  1556 | 43  519 | CTT (L) => TTT (F)  ACG (T) => ATG (M) | 0.86  0.86 |
| *rps*2 | 248 | 83 | TCA (S) => TTA (L) | 1.00 |
| *ycf*3 | 362 | 121 | ACT (T) => ATT (I) | 1.00 |

Prediction of RNA editing sites in chloroplast genes of *A. mesoleios*

| Gene | Nucleotide position | Amino acid position | RNA editing effect | Score |
| --- | --- | --- | --- | --- |
| *acc*D | 209  280  1072 | 70  94  358 | CCG (P) => CTG (L)  CAT (H) => TAT (Y) CCG (P) => TCG (S) | 1.00  1.00  1.00 |
| *atp*I | 76 | 26 | CTC (L) => TTC (F) | 0.86 |
| *ccs*A | 137  514 | 46  172 | ACA (T) => ATA (I)  CTT (L) => TTT (F) | 1.00  0.86 |
| *clp*P | 313  1099 | 105  367 | CTT (L) => TTT (F)  CAT (H) => TAT (Y) | 1.00  1.00 |
| *mat*K | 988  1187 | 330  396 | CTT (L) => TTT (F)  TCA (S) => TTA (L) | 0.86  0.86 |
| *ndh*A | 137  341  623  1157  1166  1180  1589 | 46  114  208  386  389  394  530 | GCA (A) => GTA (V)  TCA (S) => TTA (L)  ACT (T) => ATT (I)  CCC (P) => CTC (L)  CCG (P) => CTG (L)  CTT (L) => TTT (F)  ACA (T) => ATA (I) | 1.00  1.00  1.00  1.00  1.00  1.00  1.00 |
| *ndh*B | 692  878  1144  1340  1414  1418 | 231  293  382  447  472  473 | TCT (S) => TTT (F)  TCA (S) => TTA (L)  CCG (P) => TCG (S) CCG (P) => CTG (L)  CTC (L) => TTC (F)  CCA (P) => CTA (L) | 1.00  1.00  1.00  1.00  1.00  1.00 |
| *ndh*D | 2  674  878  1405 | 1  225  293  469 | ACG (T) => ATG (M)  TCG (S) => TTG (L)  TCA (S) => TTA (L)  CTT (L) => TTT (F) | 1.00  1.00  1.00  0.80 |
| *ndh*F | 13  290  586  1172 | 5  97  196  391 | CAT (H) => TAT (Y)  TCA (S) => TTA (L)  CTT (L) => TTT (F)  GCG (A) => GTG (V) | 1.00  1.00  0.80  0.80 |
| *ndh*G | 166  314  385 | 56  105  129 | CAT (H) => TAT (Y)  ACA (T) => ATA (I)  CCA (P) => TCA (S) | 0.80  0.80  0.80 |
| *pet*B | 307 | 103 | CAT (H) => TAT (Y) | 1.00 |
| *pet*D | 1048  1108  1175 | 350  370  392 | CTT (L) => TTT (F)  CTT (L) => TTT (F)  CCT (P) => CTT (L) | 1.00  1.00  1.00 |
| *psa*B | 1456 | 486 | CTC (L) => TTC (F) | 1.00 |
| *rpl*2 | 569 | 190 | ACC (T) => ATC (I) | 1.00 |
| *rpo*B | 226  338  551  566  718  2000  2426 | 76  113  184  189  240  667  809 | CAT (H) => TAT (Y)  TCT (S) => TTT (F)  TCA (S) => TTA (L)  TCG (S) => TTG (L)  CCT (P) => TCT (S)  TCT (S) => TTT (F)  TCA (S) => TTA (L) | 1.00  1.00  1.00  1.00  1.00  1.00  0.86 |
| *rpo*C1 | 127  2273 | 43  758 | CTT (L) => TTT (F)  ACG (T) => ATG (M) | 0.86  0.86 |
| *ycf*3 | 721  1814 | 241  605 | CAT (H) => TAT (Y)  ACT (T) => ATT (I) | 1.00  1.00 |

Prediction of RNA editing sites in chloroplast genes of *A. mollissimus*

| Gene | Nucleotide position | Amino acid position | RNA editing effect | Score |
| --- | --- | --- | --- | --- |
| *atp*B | 650 | 217 | ACG (T) => ATG (M) | 1.00 |
| *atp*I | 76 | 26 | CTC (L) => TTC (F) | 0.86 |
| *ccs*A | 137  514 | 46  172 | ACA (T) => ATA (I)  CTT (L) => TTT (F) | 1.00  0.86 |
| *mat*K | 988  1187 | 330  396 | CTT (L) => TTT (F)  TCA (S) => TTA (L) | 0.86  0.86 |
| *ndh*A | 137  341  1073 | 46  114  358 | GCA (A) => GTA (V)  TCA (S) => TTA (L)  TCT (S) => TTT (F) | 1.00  1.00  1.00 |
| *ndh*B | 28  746  836  1487 | 10  249  279  496 | CTC (L) => TTC (F)  TCT (S) => TTT (F)  TCA (S) => TTA (L)  CCA (P) => CTA (L) | 1.00  1.00  1.00  1.00 |
| *ndh*D | 2  674  878  1405 | 1  225  293  469 | ACG (T) => ATG (M)  TCG (S) => TTG (L)  TCA (S) => TTA (L)  CTT (L) => TTT (F) | 1.00  1.00  1.00  0.80 |
| *ndh*F | 13  290  586  1172 | 5  97  196  391 | CAT (H) => TAT (Y)  TCA (S) => TTA (L)  CTT (L) => TTT (F)  GCG (A) => GTG (V) | 1.00  1.00  0.80  0.80 |
| *ndh*G | 166  314  385 | 56  105  129 | CAT (H) => TAT (Y)  ACA (T) => ATA (I)  CCA (P) => TCA (S) | 0.80  0.80  0.80 |
| *pet*B | 611 | 204 | CCA (P) => CTA (L) | 1.00 |
| *psa*B | 1456 | 486 | CTC (L) => TTC (F) | 1.00 |
| *rpl*2 | 593 | 198 | GCA (A) => GTA (V) | 0.86 |
| *rpo*B | 226  338  551  566  718  2000  2426 | 76  113  184  189  240  667  809 | CAT (H) => TAT (Y)  TCT (S) => TTT (F)  TCA (S) => TTA (L)  TCG (S) => TTG (L)  CCT (P) => TCT (S)  TCT (S) => TTT (F)  TCA (S) => TTA (L) | 1.00  1.00  1.00  1.00  1.00  1.00  0.86 |
| *rpo*C1 | 127  1556 | 43  519 | CTT (L) => TTT (F)  ACG (T) => ATG (M) | 0.86  0.86 |
| *ycf*3 | 362 | 121 | ACT (T) => ATT (I) | 1.00 |

Prediction of RNA editing sites in chloroplast genes of *A. mongholicus*

| Gene | Nucleotide position | Amino acid position | RNA editing effect | Score |
| --- | --- | --- | --- | --- |
| *acc*D | 209  280  1078 | 70  94  360 | CCG (P) => CTG (L)  CAT (H) => TAT (Y)  CCG (P) => TCG (S) | 1.00  1.00  1.00 |
| *atp*I | 76 | 26 | CTC (L) => TTC (F) | 0.86 |
| *ccs*A | 137  514 | 46  172 | ACA (T) => ATA (I)  CTT (L) => TTT (F) | 1.00  0.86 |
| *clp*P | 313 | 105 | CTT (L) => TTT (F) | 1.00 |
| *mat*K | 994  1193 | 332  398 | CTT (L) => TTT (F)  TCA (S) => TTA (L) | 0.86  0.86 |
| *ndh*A | 137  341  1073 | 46  114  358 | GCA (A) => GTA (V)  TCA (S) => TTA (L)  TCT (S) => TTT (F) | 1.00  1.00  1.00 |
| *ndh*B | 557  692  782  1201  1433 | 186  231  261  401  478 | TCA (S) => TTA (L) TCT (S) => TTT (F)  TCA (S) => TTA (L)  CAT (H) => TAT (Y)  CCA (P) => CTA (L) | 0.80  1.00  1.00  1.00  1.00 |
| *ndh*D | 5  677  881  1408 | 2  226  294  470 | ACG (T) => ATG (M)  TCG (S) => TTG (L)  TCA (S) => TTA (L)  CTT (L) => TTT (F) | 1.00  1.00  1.00  0.80 |
| *ndh*F | 13  241  290  586  1172 | 5  81  97  196  391 | CAT (H) => TAT (Y)  CTT (L) => TTT (F)  TCA (S) => TTA (L)  CTT (L) => TTT (F)  GCG (A) => GTG (V) | 1.00  1.00  0.80  0.80  0.80 |
| *ndh*G | 166  314  385 | 56  105  129 | CAT (H) => TAT (Y)  ACA (T) => ATA (I)  CCA (P) => TCA (S) | 0.80  0.80  0.80 |
| *pet*B | 611 | 204 | CCA (P) => CTA (L) | 1.00 |
| *psa*B | 1456 | 486 | CTC (L) => TTC (F) | 1.00 |
| *rpo*B | 338  551  566  718  2000  2426 | 113  184  189  240  667  809 | TCT (S) => TTT (F)  TCA (S) => TTA (L)  TCG (S) => TTG (L)  CCT (P) => TCT (S)  TCT (S) => TTT (F)  TCA (S) => TTA (L) | 1.00  1.00  1.00  1.00  1.00  0.86 |
| *rpo*C1 | 127  1556 | 43  519 | CTT (L) => TTT (F)  ACG (T) => ATG (M) | 0.86  0.86 |
| *rps*2 | 248 | 83 | TCA (S) => TTA (L) | 1.00 |
| *ycf*3 | 377  410 | 126  137 | TCT (S) => TTT (F)  GCT (A) => GTT (V) | 1.00  0.86 |

Prediction of RNA editing sites in chloroplast genes of *A. nakaianus*

| Gene | Nucleotide position | Amino acid position | RNA editing effect | Score |
| --- | --- | --- | --- | --- |
| *acc*D | 209  280  1078 | 70  94  360 | CCG (P) => CTG (L)  CAT (H) => TAT (Y)  CCG (P) => TCG (S) | 1.00  1.00  1.00 |
| *atp*I | 76 | 26 | CTC (L) => TTC (F) | 0.86 |
| *ccs*A | 137  514 | 46  172 | ACA (T) => ATA (I)  CTT (L) => TTT (F) | 1.00  0.86 |
| *clp*P | 313 | 105 | CTT (L) => TTT (F) | 1.00 |
| *mat*K | 994  1193 | 332  398 | CTT (L) => TTT (F)  TCA (S) => TTA (L) | 0.86  0.86 |
| *ndh*A | 137  341  1073 | 46  114  358 | GCA (A) => GTA (V)  TCA (S) => TTA (L)  TCT (S) => TTT (F) | 1.00  1.00  1.00 |
| *ndh*B | 557  692  782  1201  1433 | 186  231  261  401  478 | TCA (S) => TTA (L) TCT (S) => TTT (F)  TCA (S) => TTA (L)  CAT (H) => TAT (Y)  CCA (P) => CTA (L) | 0.80  1.00  1.00  1.00  1.00 |
| *ndh*D | 5  677  881  1408 | 2  226  294  470 | ACG (T) => ATG (M)  TCG (S) => TTG (L)  TCA (S) => TTA (L)  CTT (L) => TTT (F) | 1.00  1.00  1.00  0.80 |
| *ndh*F | 13  241  290  586  1172 | 5  81  97  196  391 | CAT (H) => TAT (Y)  CTT (L) => TTT (F)  TCA (S) => TTA (L)  CTT (L) => TTT (F)  GCG (A) => GTG (V) | 1.00  1.00  1.00  0.80  0.80 |
| *ndh*G | 166  314  385 | 56  105  129 | CAT (H) => TAT (Y)  ACA (T) => ATA (I)  CCA (P) => TCA (S) | 0.80  0.80  0.80 |
| *pet*B | 611 | 204 | CCA (P) => CTA (L) | 1.00 |
| *psa*B | 1456 | 486 | CTC (L) => TTC (F) | 1.00 |
| *rpo*B | 338  551  566  718  2000  2426 | 113  184  189  240  667  809 | TCT (S) => TTT (F)  TCA (S) => TTA (L)  TCG (S) => TTG (L)  CCT (P) => TCT (S)  TCT (S) => TTT (F)  TCA (S) => TTA (L) | 1.00  1.00  1.00  1.00  1.00  0.86 |
| *rpo*C1 | 127  1556 | 43  519 | CTT (L) => TTT (F)  ACG (T) => ATG (M) | 0.86  0.86 |
| *rps*2 | 248 | 83 | TCA (S) => TTA (L) | 1.00 |
| *ycf*3 | 362 | 121 | ACT (T) => ATT (I) | 1.00 |

Prediction of RNA editing sites in chloroplast genes of *A. neglectus*

| Gene | Nucleotide position | Amino acid position | RNA editing effect | Score |
| --- | --- | --- | --- | --- |
| *acc*D | 221  292 | 74  98 | CCG (P) => CTG (L)  CAT (H) => TAT (Y) | 1.00  1.00 |
| *atp*I | 76 | 26 | CTC (L) => TTC (F) | 0.86 |
| *ccs*A | 137  514 | 46  172 | ACA (T) => ATA (I)  CTT (L) => TTT (F) | 1.00  0.86 |
| *mat*K | 988  1187 | 330  396 | CTT (L) => TTT (F)  TCA (S) => TTA (L) | 0.86  0.86 |
| *ndh*A | 137  341  1073 | 46  114  358 | GCA (A) => GTA (V)  TCA (S) => TTA (L)  TCT (S) => TTT (F) | 1.00  1.00  1.00 |
| *ndh*B | 28  746  836  1487 | 10  249  279  496 | CTC (L) => TTC (F) TCT (S) => TTT (F)  TCA (S) => TTA (L)  CCA (P) => CTA (L) | 1.00  1.00  1.00  1.00 |
| *ndh*D | 2  674  878  1405 | 1  225  293  469 | ACG (T) => ATG (M)  TCG (S) => TTG (L)  TCA (S) => TTA (L)  CTT (L) => TTT (F) | 1.00  1.00  1.00  0.80 |
| *ndh*F | 13  290  586  1172 | 5  97  196  391 | CAT (H) => TAT (Y)  TCA (S) => TTA (L)  CTT (L) => TTT (F)  GCG (A) => GTG (V) | 1.00  1.00  0.80  0.80 |
| *ndh*G | 166  314  385 | 56  105  129 | CAT (H) => TAT (Y)  ACA (T) => ATA (I)  CCA (P) => TCA (S) | 0.80  0.80  0.80 |
| *pet*B | 611 | 204 | CCA (P) => CTA (L) | 1.00 |
| *psa*B | 1456 | 486 | CTC (L) => TTC (F) | 1.00 |
| *rpl*2 | 593 | 198 | GCA (A) => GTA (V) | 0.86 |
| *rpo*B | 226  338  551  566  718  2000  2426 | 76  113  184  189  240  667  809 | CAT (H) => TAT (Y)  TCT (S) => TTT (F)  TCA (S) => TTA (L)  TCG (S) => TTG (L)  CCT (P) => TCT (S)  TCT (S) => TTT (F)  TCA (S) => TTA (L) | 1.00  1.00  1.00  1.00  1.00  1.00  0.86 |
| *rpo*C1 | 127  1556 | 43  519 | CTT (L) => TTT (F)  ACG (T) => ATG (M) | 0.86  0.86 |
| *ycf*3 | 362 | 121 | ACT (T) => ATT (I) | 1.00 |

Prediction of RNA editing sites in chloroplast genes of *A. nuttallianus*

| Gene | Nucleotide position | Amino acid position | RNA editing effect | Score |
| --- | --- | --- | --- | --- |
| *acc*D | 209  280  833  1150  1186 | 70  94  278  384  396 | TCG (S) => TTG (L) CAT (H) => TAT (Y)  GCC (A) => GTC (V)  CAT (H) => TAT (Y)  CCA (P) => TCA (S) | 1.00  1.00  0.80  0.80  1.00 |
| *atp*I | 76 | 26 | CTC (L) => TTC (F) | 0.86 |
| *ccs*A | 137  514 | 46  172 | ACA (T) => ATA (I)  CTT (L) => TTT (F) | 1.00  0.86 |
| *clp*P | 313 | 105 | CTT (L) => TTT (F) | 1.00 |
| *mat*K | 830  988  1187 | 277  330  396 | GCT (A) => GTT (V)  CTT (L) => TTT (F)  TCA (S) => TTA (L) | 1.00  0.86  0.86 |
| *ndh*A | 137  341  1073 | 46  114  358 | GCA (A) => GTA (V)  TCA (S) => TTA (L)  TCT (S) => TTT (F) | 1.00  1.00  1.00 |
| *ndh*B | 28  746  836  1487 | 10  249  279  496 | CTC (L) => TTC (F) TCT (S) => TTT (F)  TCA (S) => TTA (L)  CCA (P) => CTA (L) | 1.00  1.00  1.00  1.00 |
| *ndh*D | 2  674  878  1405 | 1  225  293  469 | ACG (T) => ATG (M)  TCG (S) => TTG (L)  TCA (S) => TTA (L)  CTT (L) => TTT (F) | 1.00  1.00  1.00  0.80 |
| *ndh*F | 13  290  586  1202 | 5  97  196  401 | CAT (H) => TAT (Y)  TCA (S) => TTA (L)  CTT (L) => TTT (F)  GCG (A) => GTG (V) | 1.00  1.00  0.80  0.80 |
| *ndh*G | 166  314  385 | 56  105  129 | CAT (H) => TAT (Y)  ACA (T) => ATA (I)  CCA (P) => TCA (S) | 0.80  0.80  0.80 |
| *pet*B | 611 | 204 | CCA (P) => CTA (L) | 1.00 |
| *psa*B | 1456 | 486 | CTC (L) => TTC (F) | 1.00 |
| *rpl*2 | 593 | 198 | GCA (A) => GTA (V) | 0.86 |
| *rpo*B | 226  338  551  566  718  2000  2426 | 76  113  184  189  240  667  809 | CAT (H) => TAT (Y)  TCT (S) => TTT (F)  TCA (S) => TTA (L)  TCG (S) => TTG (L)  CCT (P) => TCT (S)  TCT (S) => TTT (F)  TCA (S) => TTA (L) | 1.00  1.00  1.00  1.00  1.00  1.00  0.86 |
| *rpo*C1 | 127  1556 | 43  519 | CTT (L) => TTT (F)  ACG (T) => ATG (M) | 0.86  0.86 |
| *ycf*3 | 362 | 121 | ACT (T) => ATT (I) | 1.00 |

Prediction of RNA editing sites in chloroplast genes of *A. odoratus*

| Gene | Nucleotide position | Amino acid position | RNA editing effect | Score |
| --- | --- | --- | --- | --- |
| *acc*D | 209  283  1075 | 70  95  359 | CCG (P) => CTG (L) CAT (H) => TAT (Y)  CCG (P) => TCG (S) | 1.00  1.00  1.00 |
| *atp*I | 76 | 26 | CTC (L) => TTC (F) | 0.86 |
| *ccs*A | 137  514 | 46  172 | ACA (T) => ATA (I)  CTT (L) => TTT (F) | 1.00  0.86 |
| *clp*P | 313 | 105 | CTT (L) => TTT (F) | 1.00 |
| *mat*K | 988  1187 | 330  396 | CTT (L) => TTT (F)  TCA (S) => TTA (L) | 0.86  0.86 |
| *ndh*A | 137  341  1073 | 46  114  358 | GCA (A) => GTA (V)  TCA (S) => TTA (L)  TCT (S) => TTT (F) | 1.00  1.00  1.00 |
| *ndh*B | 557  692  782  1433 | 186  231  261  478 | TCA (S) => TTA (L)  TCT (S) => TTT (F)  TCA (S) => TTA (L)  CCA (P) => CTA (L) | 0.80  1.00  1.00  1.00 |
| *ndh*D | 2  674  1405 | 1  225  469 | ACG (T) => ATG (M)  TCG (S) => TTG (L)  CTT (L) => TTT (F) | 1.00  1.00  0.80 |
| *ndh*F | 13  290  586  1172 | 5  97  196  391 | CAT (H) => TAT (Y)  TCA (S) => TTA (L)  CTT (L) => TTT (F)  GCG (A) => GTG (V) | 1.00  1.00  0.80  0.80 |
| *ndh*G | 166  314  385 | 56  105  129 | CAT (H) => TAT (Y)  ACA (T) => ATA (I)  CCA (P) => TCA (S) | 0.80  0.80  0.80 |
| *pet*B | 611 | 204 | CCA (P) => CTA (L) | 1.00 |
| *psa*B | 1456 | 486 | CTC (L) => TTC (F) | 1.00 |
| *rpl*2 | 596 | 199 | GCA (A) => GTA (V) | 0.86 |
| *rpo*B | 226  338  551  566  718  2000  2426 | 76  113  184  189  240  667  809 | CAT (H) => TAT (Y)  TCT (S) => TTT (F)  TCA (S) => TTA (L)  TCG (S) => TTG (L)  CCT (P) => TCT (S)  TCT (S) => TTT (F)  TCA (S) => TTA (L) | 1.00  1.00  1.00  1.00  1.00  1.00  0.86 |
| *rpo*C1 | 127  1556 | 43  519 | CTT (L) => TTT (F)  ACG (T) => ATG (M) | 0.86  0.86 |
| *ycf*3 | 362 | 121 | ACT (T) => ATT (I) | 1.00 |

Prediction of RNA editing sites in chloroplast genes of *A. pectinatus*

| Gene | Nucleotide position | Amino acid position | RNA editing effect | Score |
| --- | --- | --- | --- | --- |
| *acc*D | 209  280  1228 | 70  94  410 | CCG (P) => CTG (L) CAT (H) => TAT (Y)  CCG (P) => TCG (S) | 1.00  1.00  1.00 |
| *atp*B | 506 | 169 | ACC (T) => ATC (I) | 1.00 |
| *atp*I | 76 | 26 | CTC (L) => TTC (F) | 0.86 |
| *ccs*A | 137  514 | 46  172 | ACA (T) => ATA (I)  CTT (L) => TTT (F) | 1.00  0.86 |
| *mat*K | 988  1187 | 330  396 | CTT (L) => TTT (F)  TCA (S) => TTA (L) | 0.86  0.86 |
| *ndh*A | 137  341  1073 | 46  114  358 | GCA (A) => GTA (V)  TCA (S) => TTA (L)  TCT (S) => TTT (F) | 1.00  1.00  1.00 |
| *ndh*B | 28  746  836  1487 | 10  249  279  496 | CTC (L) => TTC (F) TCT (S) => TTT (F)  TCA (S) => TTA (L)  CCA (P) => CTA (L) | 1.00  1.00  1.00  1.00 |
| *ndh*D | 2  674  878  1405 | 1  225  293  469 | ACG (T) => ATG (M)  TCG (S) => TTG (L)  TCA (S) => TTA (L)  CTT (L) => TTT (F) | 1.00  1.00  1.00  0.80 |
| *ndh*F | 13  290  586  1172 | 5  97  196  391 | CAT (H) => TAT (Y)  TCA (S) => TTA (L)  CTT (L) => TTT (F)  GCG (A) => GTG (V) | 1.00  1.00  0.80  0.80 |
| *ndh*G | 166  314  385 | 56  105  129 | CAT (H) => TAT (Y)  ACA (T) => ATA (I)  CCA (P) => TCA (S) | 0.80  0.80  0.80 |
| *pet*B | 611 | 204 | CCA (P) => CTA (L) | 1.00 |
| *psa*B | 1456 | 486 | CTC (L) => TTC (F) | 1.00 |
| *rpl*2 | 593 | 198 | GCA (A) => GTA (V) | 0.86 |
| *rpo*B | 226  338  551  566  718  2000  2426 | 76  113  184  189  240  667  809 | CAT (H) => TAT (Y)  TCT (S) => TTT (F)  TCA (S) => TTA (L)  TCG (S) => TTG (L)  CCT (P) => TCT (S)  TCT (S) => TTT (F)  TCA (S) => TTA (L) | 1.00  1.00  1.00  1.00  1.00  1.00  0.86 |
| *rpo*C1 | 127  1556 | 43  519 | CTT (L) => TTT (F)  ACG (T) => ATG (M) | 0.86  0.86 |
| *ycf*3 | 362 | 121 | ACT (T) => ATT (I) | 1.00 |

Prediction of RNA editing sites in chloroplast genes of *A. scaberrimus*

| Gene | Nucleotide position | Amino acid position | RNA editing effect | Score |
| --- | --- | --- | --- | --- |
| *acc*D | 209  283  1069 | 70  95  357 | CCG (P) => CTG (L) CAT (H) => TAT (Y)  CCG (P) => TCG (S) | 1.00  1.00  1.00 |
| *atp*I | 76 | 26 | CTC (L) => TTC (F) | 0.86 |
| *ccs*A | 137  514 | 46  172 | ACA (T) => ATA (I)  CTT (L) => TTT (F) | 1.00  0.86 |
| *clp*P | 313 | 105 | CTT (L) => TTT (F) | 1.00 |
| *mat*K | 988  1187 | 330  396 | CTT (L) => TTT (F)  TCA (S) => TTA (L) | 0.86  0.86 |
| *ndh*A | 137  341  1073 | 46  114  358 | GCA (A) => GTA (V)  TCA (S) => TTA (L)  TCT (S) => TTT (F) | 1.00  1.00  1.00 |
| *ndh*B | 557  692  782  1433 | 186  231  261  478 | TCA (S) => TTA (L)  TCT (S) => TTT (F)  TCA (S) => TTA (L)  CCA (P) => CTA (L) | 0.80  1.00  1.00  1.00 |
| *ndh*D | 5  677  881  1408 | 2  226  294  470 | ACG (T) => ATG (M)  TCG (S) => TTG (L)  TCA (S) => TTA (L)  CTT (L) => TTT (F) | 1.00  1.00  1.00  0.80 |
| *ndh*F | 13  290  586  1172 | 5  97  196  391 | CAT (H) => TAT (Y)  TCA (S) => TTA (L)  CTT (L) => TTT (F)  GCG (A) => GTG (V) | 1.00  1.00  0.80  0.80 |
| *ndh*G | 202  350  421 | 68  117  141 | CAT (H) => TAT (Y)  ACA (T) => ATA (I)  CCA (P) => TCA (S) | 0.80  0.80  0.80 |
| *pet*B | 611 | 204 | CCA (P) => CTA (L) | 1.00 |
| *psa*B | 1456 | 486 | CTC (L) => TTC (F) | 1.00 |
| *rpo*B | 226  338  551  566  718  2000  2426 | 76  113  184  189  240  667  809 | CAT (H) => TAT (Y)  TCT (S) => TTT (F)  TCA (S) => TTA (L)  TCG (S) => TTG (L)  CCT (P) => TCT (S)  TCT (S) => TTT (F)  TCA (S) => TTA (L) | 1.00  1.00  1.00  1.00  1.00  1.00  0.86 |
| *rpo*C1 | 130  1559 | 44  520 | CTT (L) => TTT (F)  ACG (T) => ATG (M) | 0.86  0.86 |
| *ycf*3 | 362 | 121 | ACT (T) => ATT (I) | 1.00 |

Prediction of RNA editing sites in chloroplast genes of *A. strictus*

| Gene | Nucleotide position | Amino acid position | RNA editing effect | Score |
| --- | --- | --- | --- | --- |
| *acc*D | 209  283  1075 | 70  95  359 | CCG (P) => CTG (L) CAT (H) => TAT (Y)  CCG (P) => TCG (S) | 1.00  1.00  1.00 |
| *atp*I | 76 | 26 | CTC (L) => TTC (F) | 0.86 |
| *ccs*A | 137 | 46 | ACA (T) => ATA (I) | 1.00 |
| *clp*P | 313 | 105 | CTT (L) => TTT (F) | 1.00 |
| *mat*K | 988  1187 | 330  396 | CTT (L) => TTT (F)  TCA (S) => TTA (L) | 0.86  0.86 |
| *ndh*A | 137  341  1073 | 46  114  358 | GCA (A) => GTA (V)  TCA (S) => TTA (L)  TCT (S) => TTT (F) | 1.00  1.00  1.00 |
| *ndh*B | 557  692  782  1433 | 186  231  261  478 | TCA (S) => TTA (L)  TCT (S) => TTT (F)  TCA (S) => TTA (L)  CCA (P) => CTA (L) | 0.80  1.00  1.00  1.00 |
| *ndh*D | 5  677  881  1408 | 2  226  294  470 | ACG (T) => ATG (M)  TCG (S) => TTG (L)  TCA (S) => TTA (L)  CTT (L) => TTT (F) | 1.00  1.00  1.00  0.80 |
| *ndh*F | 13  290  586  1172 | 5  97  196  391 | CAT (H) => TAT (Y)  TCA (S) => TTA (L)  CTT (L) => TTT (F)  GCG (A) => GTG (V) | 1.00  1.00  0.80  0.80 |
| *ndh*G | 145  166  314  385 | 49  56  105  129 | CTC (L) => TTC (F)  CAT (H) => TAT (Y)  ACA (T) => ATA (I)  CCA (P) => TCA (S) | 0.80  0.80  0.80  0.80 |
| *pet*B | 611 | 204 | CCA (P) => CTA (L) | 1.00 |
| *psa*B | 1456 | 486 | CTC (L) => TTC (F) | 1.00 |
| *rpo*B | 226  338  551  566  718  2000  2426 | 76  113  184  189  240  667  809 | CAT (H) => TAT (Y)  TCT (S) => TTT (F)  TCA (S) => TTA (L)  TCG (S) => TTG (L)  CCT (P) => TCT (S)  TCT (S) => TTT (F)  TCA (S) => TTA (L) | 1.00  1.00  1.00  1.00  1.00  1.00  0.86 |
| *rpo*C1 | 127  1556 | 43  519 | CTT (L) => TTT (F)  ACG (T) => ATG (M) | 0.86  0.86 |
| *ycf*3 | 362 | 121 | ACT (T) => ATT (I) | 1.00 |
