# Supplementary material for: Characterization and comparative analysis of the complete plastid genomes of four Astragalus species
Source: PLoS One. 2023 May 23;18(5):e0286083. doi: 10.1371/journal.pone.0286083 (PMC10204964; doi:10.1371/journal.pone.0286083)
Supplement: S1 Fig — Genome regions are color coded as protein coding, rRNA coding, tRNA coding, or conserved noncoding sequences. The vertical scale indicates the percentage identity, ranging from 50% to 100%. (PDF) [file pone.0286083.s009.pdf]

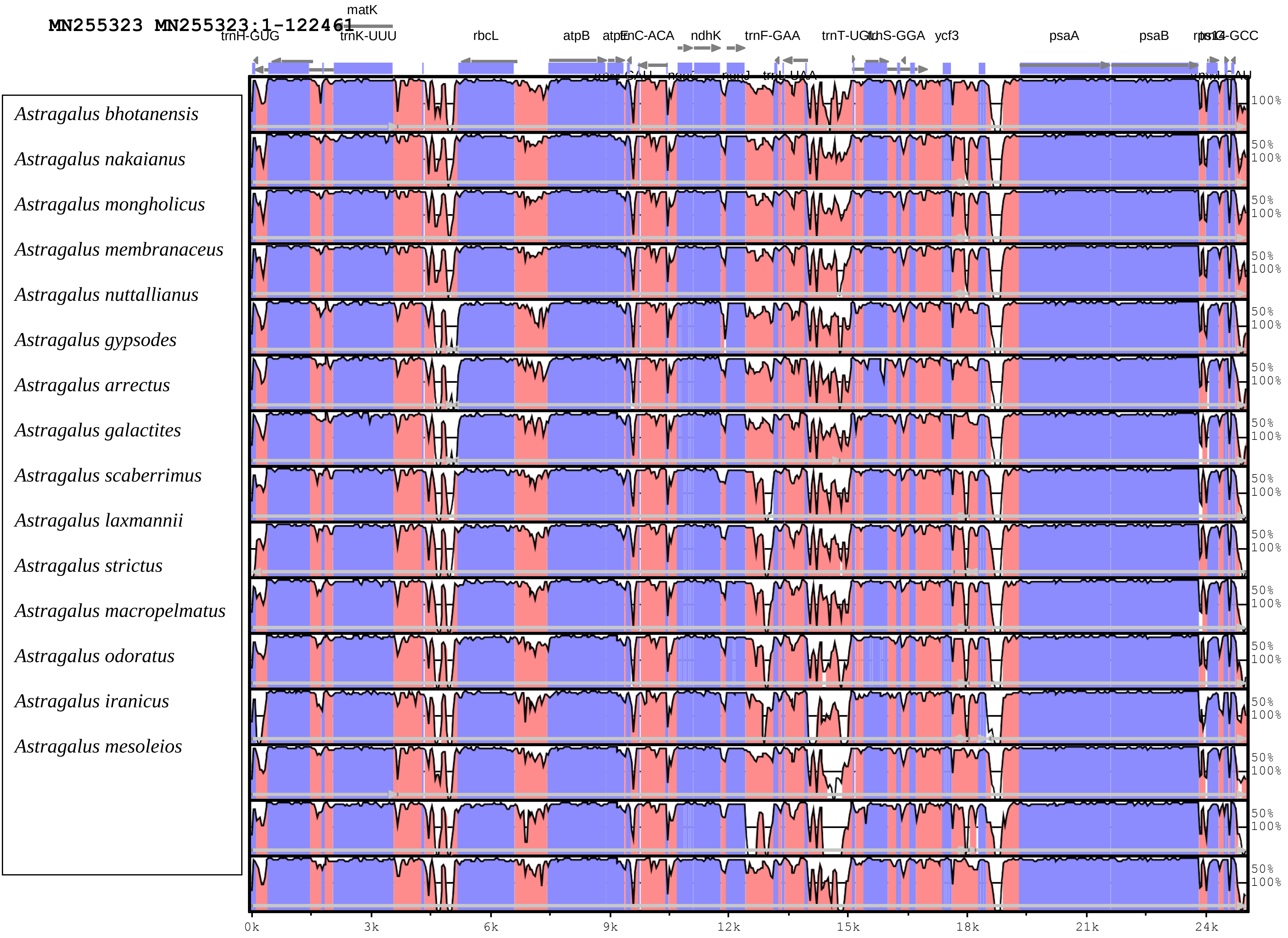
MN255323 MN255323:1-122461
matK
trnH-GUG
trnK-UUU
rbcL
atpB
ndhK
trnF-GAA
ycf3
psaA
psaB
Astragalus bhotanensis
Astragalus nakaianus
Astragalus mongholicus
Astragalus membranaceus
Astragalus nuttallianus
Astragalus gypsodes
Astragalus arrectus
Astragalus galactites
Astragalus scaberrimus
Astragalus laxmannii
Astragalus strictus
Astragalus macropelmatus
Astragalus odoratus
Astragalus iranicus
Astragalus mesoleios
100%
50%
0k
3k
6k
9k
12k
15k
18k
21k
24k

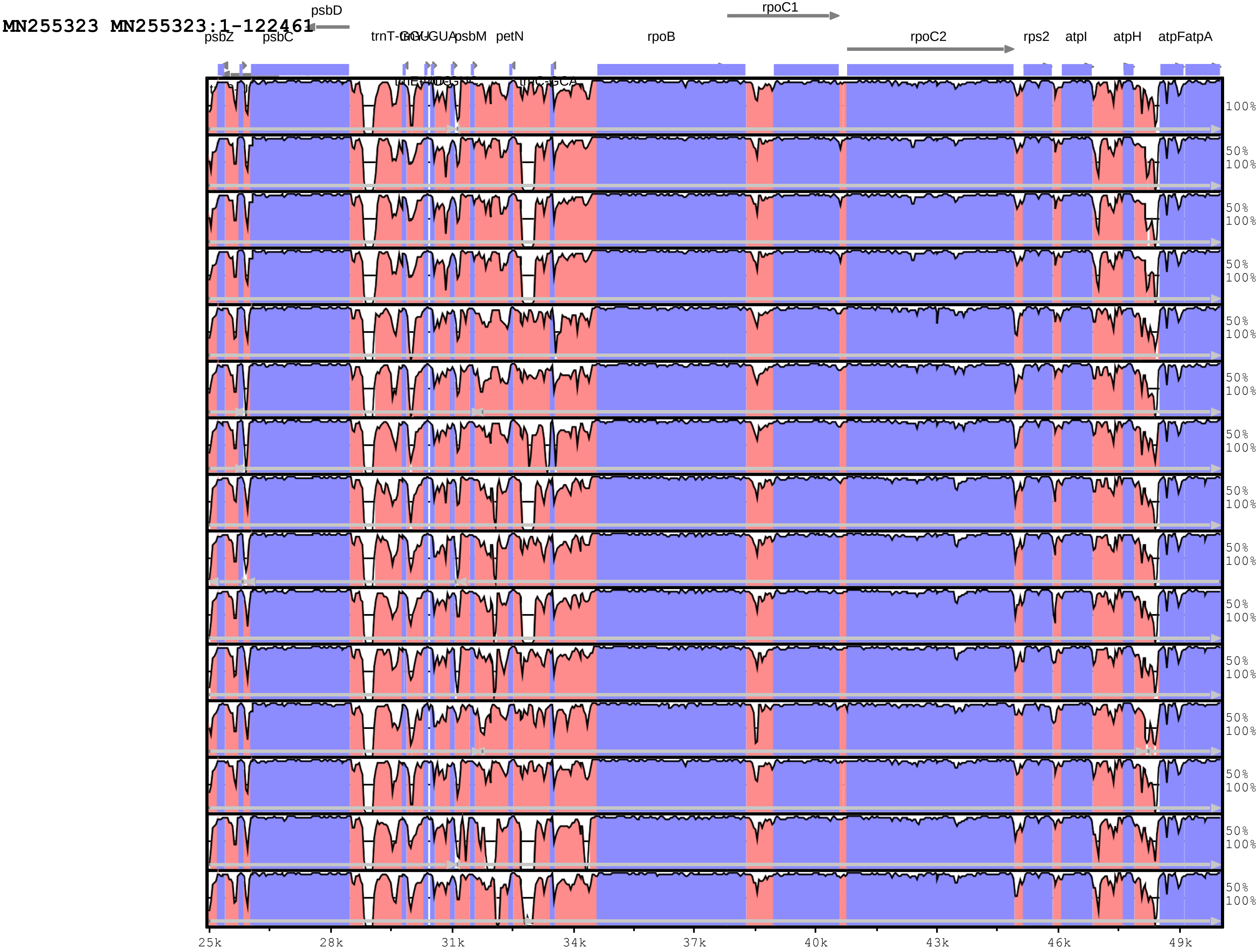

MN255323 MN255323:1-122461
psbD
rpoC1
psbZ
psbC
psbM
petN
rpoB
rpoC2
rps2
atpI
atpH
atpFatpA
100%
50%
25k
28k
31k
34k
37k
40k
43k
46k
49k

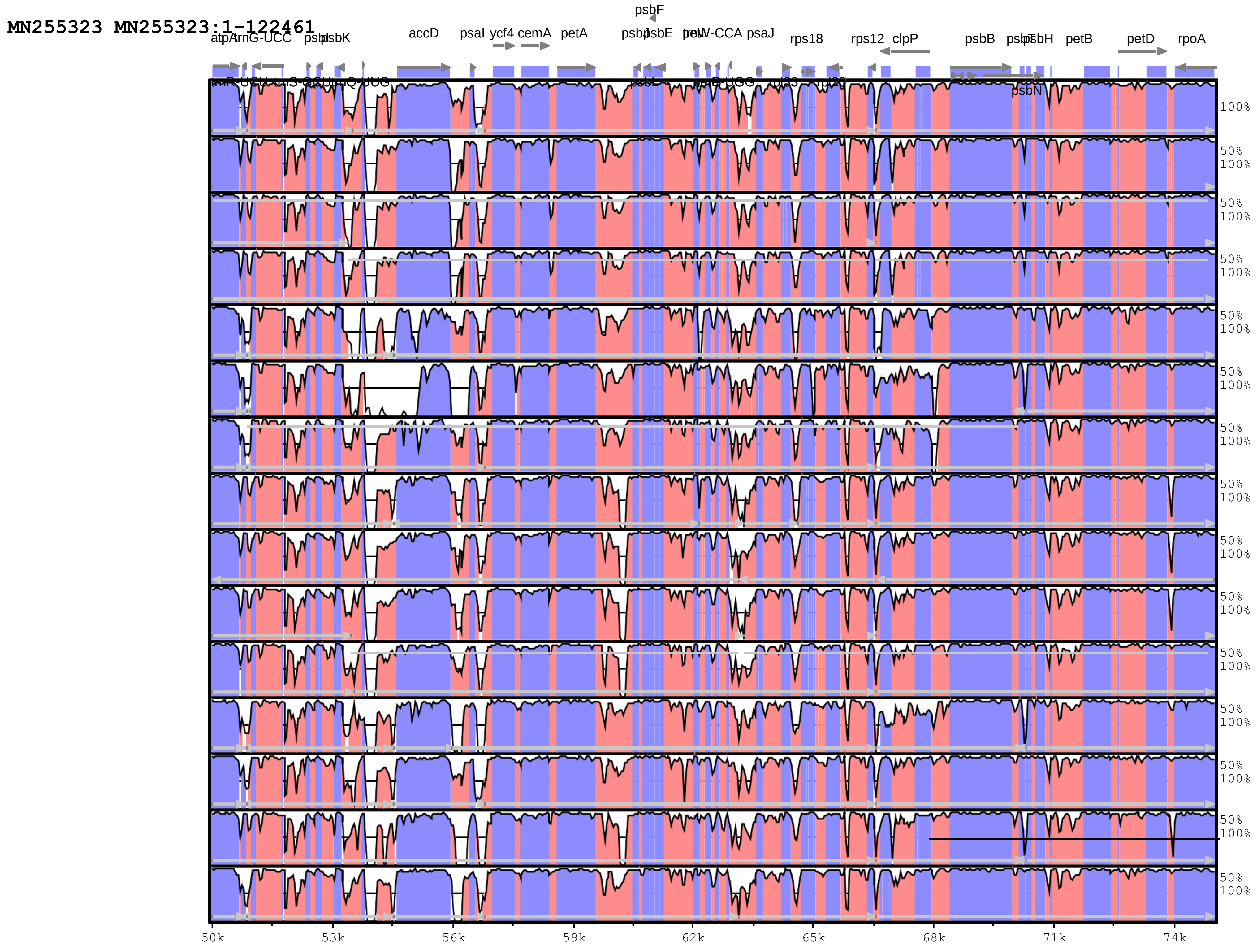
MN255323 MN255323:1-122461
atpA
trnG-UCC
psbI
psbK
accD
psaI
ycf4
cemA
petA
psbF
psbJ
psbE
petL
trnW-CCA
psaJ
rps18
rps12
clpP
psbB
psbT
psbH
petB
petD
rpoA
trnR-UCU
trnS-GCU
trnQ-UUG
psbL
trnP-UGG
rpl33
rpl20
psbN
50%
100%
50k
53k
56k
59k
62k
65k
68k
71k
74k

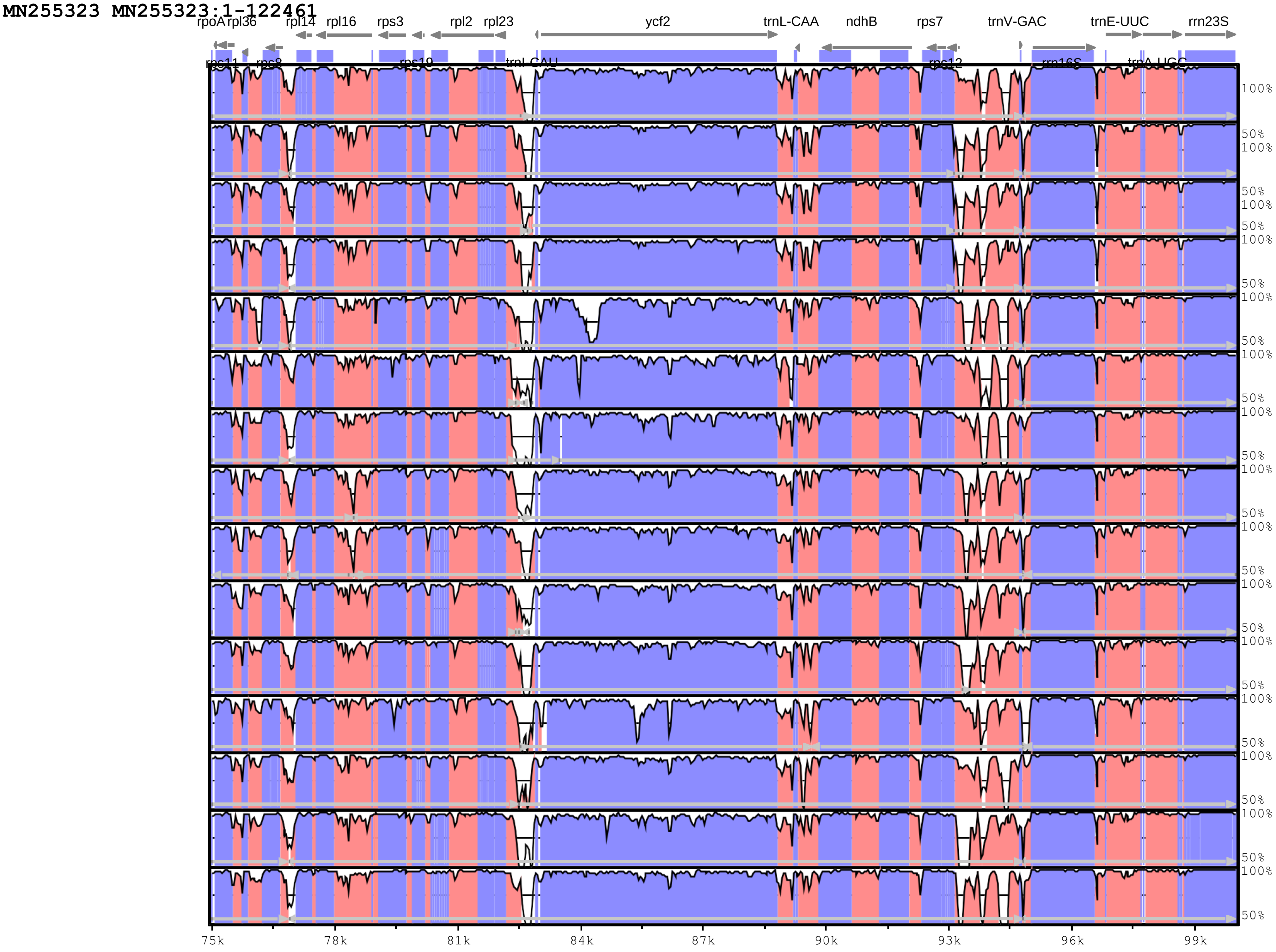
MN255323 MN255323:1-122461
rpoA
rpl36
rpl14
rpl16
rps3
rpl2
rpl23
ycf2
trnL-CAA
ndhB
rps7
trnV-GAC
trnE-UUC
rrn23S
rps11
rps8
rps19
trnI-CAU
rps12
rrn16S
trnA-UGC
100%
50%
75k
78k
81k
84k
87k
90k
93k
96k
99k

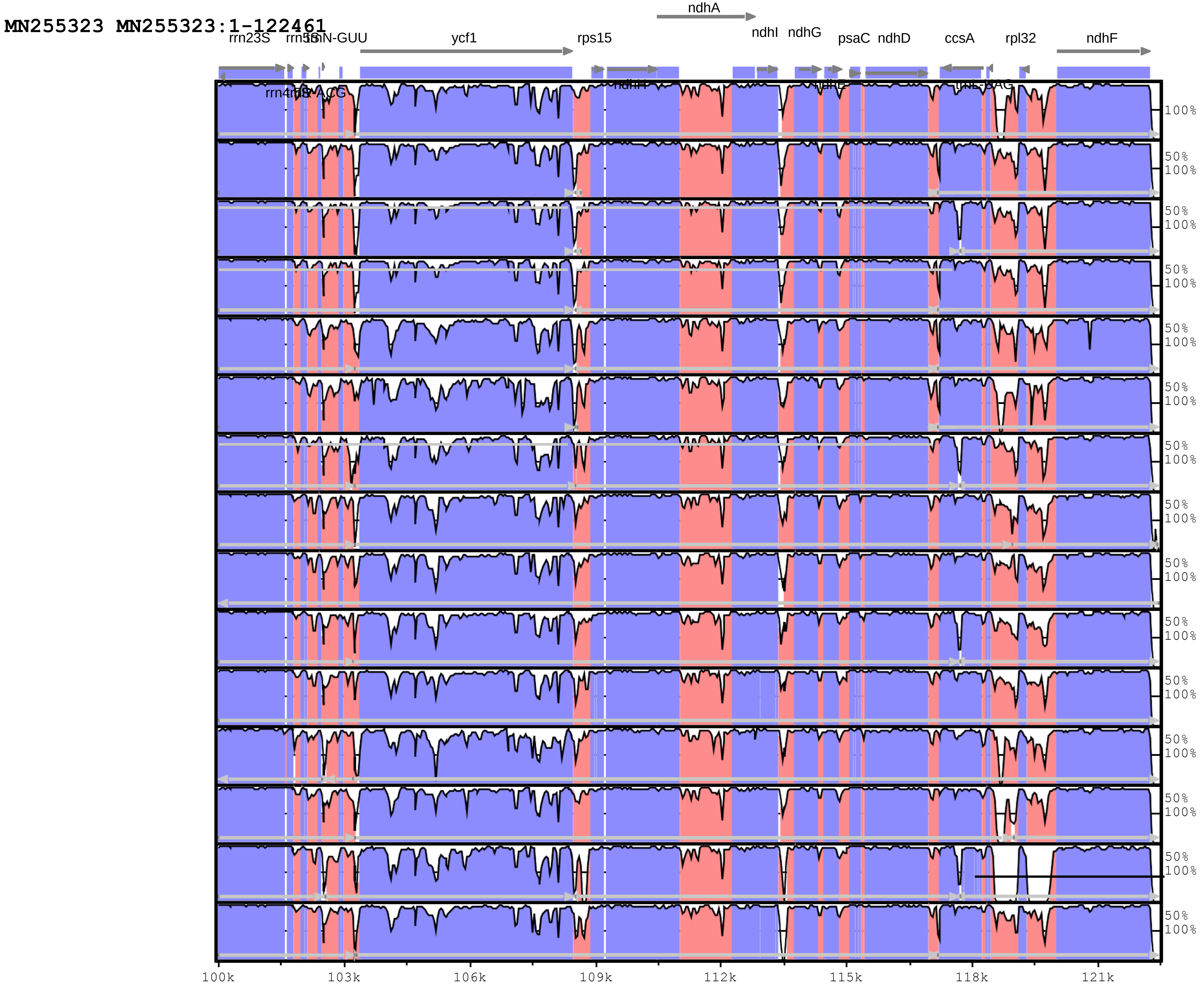

MN255323 MN255323:1-122461
ndhA
rrn23S
rrn5S
trnN-GUU
ycf1
rps15
ndhI
ndhG
psaC
ndhD
ccsA
rpl32
ndhF
ndhH
ndhE
50%
100%
100k
103k
106k
109k
112k
115k
118k
121k

5
0
%

100k 103k 106k 109k 112k 115k 118k 121k
